# On the Analyses of Medical Images Using Traditional Machine Learning Techniques and Convolutional Neural Networks

**DOI:** 10.1007/s11831-023-09899-9

**Published:** 2023-04-04

**Authors:** Saeed Iqbal, Adnan N. Qureshi, Jianqiang Li, Tariq Mahmood

**Affiliations:** 1 Department of Computer Science, Faculty of Information Technology & Computer Science, University of Central Punjab, Lahore, Punjab 54000 Pakistan; 2 Faculty of Information Technology, Beijing University of Technology, Beijing, 100124 Beijing China; 3 Beijing Engineering Research Center for IoT Software and Systems, Beijing University of Technology, Beijing, 100124 Beijing China; 4 Artificial Intelligence and Data Analytics (AIDA) Lab, College of Computer & Information Sciences (CCIS), Prince Sultan University, Riyadh, 11586 Kingdom of Saudi Arabia

## Abstract

Convolutional neural network (CNN) has shown dissuasive accomplishment on different areas especially Object Detection, Segmentation, Reconstruction (2D and 3D), Information Retrieval, Medical Image Registration, Multi-lingual translation, Local language Processing, Anomaly Detection on video and Speech Recognition. CNN is a special type of Neural Network, which has compelling and effective learning ability to learn features at several steps during augmentation of the data. Recently, different interesting and inspiring ideas of Deep Learning (DL) such as different activation functions, hyperparameter optimization, regularization, momentum and loss functions has improved the performance, operation and execution of CNN Different internal architecture innovation of CNN and different representational style of CNN has significantly improved the performance. This survey focuses on internal taxonomy of deep learning, different models of vonvolutional neural network, especially depth and width of models and in addition CNN components, applications and current challenges of deep learning.

## Introduction

For many disorders, medical imaging is a crucial screening method. In 1895, Roentgen revealed that X-rays could be utilized to examine into the internal organs without causing any harm. Shortly after, X-ray radiology evolved into the earliest method for diagnosing diseases. Since then, a variety of imaging techniques have been created, with Computed Tomography (CT) scanning, Positron Emission Tomography (PET), ultrasonography and Magnetic Resonance Imaging (MRI) being some of the most widely utilized. Additionally, increasingly intricate scanning techniques have been created. At numerous points in the healthcare system, encompassing diagnosis, characterization, grading, clinical outcome evaluation, tracking of tumor recurrence, as well as directing intervention treatments, surgeries and radiosurgery, image information is vital to judgment and finalizing decision.

Minimal two-dimensional (2D) imagegraphs are used for a specific individual instance, but several are used for 3D imaging and vast numbers are used for 4D interactive imaging. The quantity of image data that has to be evaluated is significantly increased by the use of multi-modality imaging. It is challenging for medical practitioners and radiologists and doctors to sustain operational efficiencies while employing all the diagnostic data at their disposal to increase reliability and individual treatment as a result of the rising burden. The promise and necessity of creating computerized tools to aid medical practitioners and radiologists in image interpretation and detection have been recognized as a significant field of study and advancement in medical imaging in light of current developments in computer vision and computational approaches.

Beginning in the 1960 s, attempts were made to use machines to autonomously interpret healthcare images [1–3]. Numerous research showed that using a machine to analyze clinical data was feasible, but the research received little focus, most likely due to the lack of accessibility to slightly elevated digitized visual information and computing power. In the 1980 s, [4] at the University of Chicago’s Kurt Rossmann Laboratory started systematically developing machine learning and image analysis methods for medical data with the intention of creating Computer-Aided Diagnosis (CAD) as a better solution to support radiologists in visual explanation [5]. In order to recognize microcalcifications on mammography, [6] created a CAD platform and carried out the initial spectator ability research that showed how well CAD improved breast radiologists’ capability to recognize microcalcifications [7]. In 1998, the Food and Drug Administration (FDA) authorized the use of the initial professional CAD system as a backup for diagnostic radiography. Over the recent decades, one of the main areas of study and innovation in diagnostic imaging has been CAD and computer-assisted image recognition.

Multiple medical disorders in both youngsters and people can now be diagnosed and treated more accurately thanks to medical imaging. Medical imaging processes come in a variety of forms, or modalities, and each one employs a unique set of tools and methods. Ionizing radiation is used by radiography, particularly Computed Tomography (CT), mammography and fluoroscopy to provide images of the human body. The risk of acquiring disease throughout the course of one’s lifetime may increase if an individual is exposed to ionising radiation, a type of radiation with enough intensity to possibly harm DNA.

**Fig. 1:**
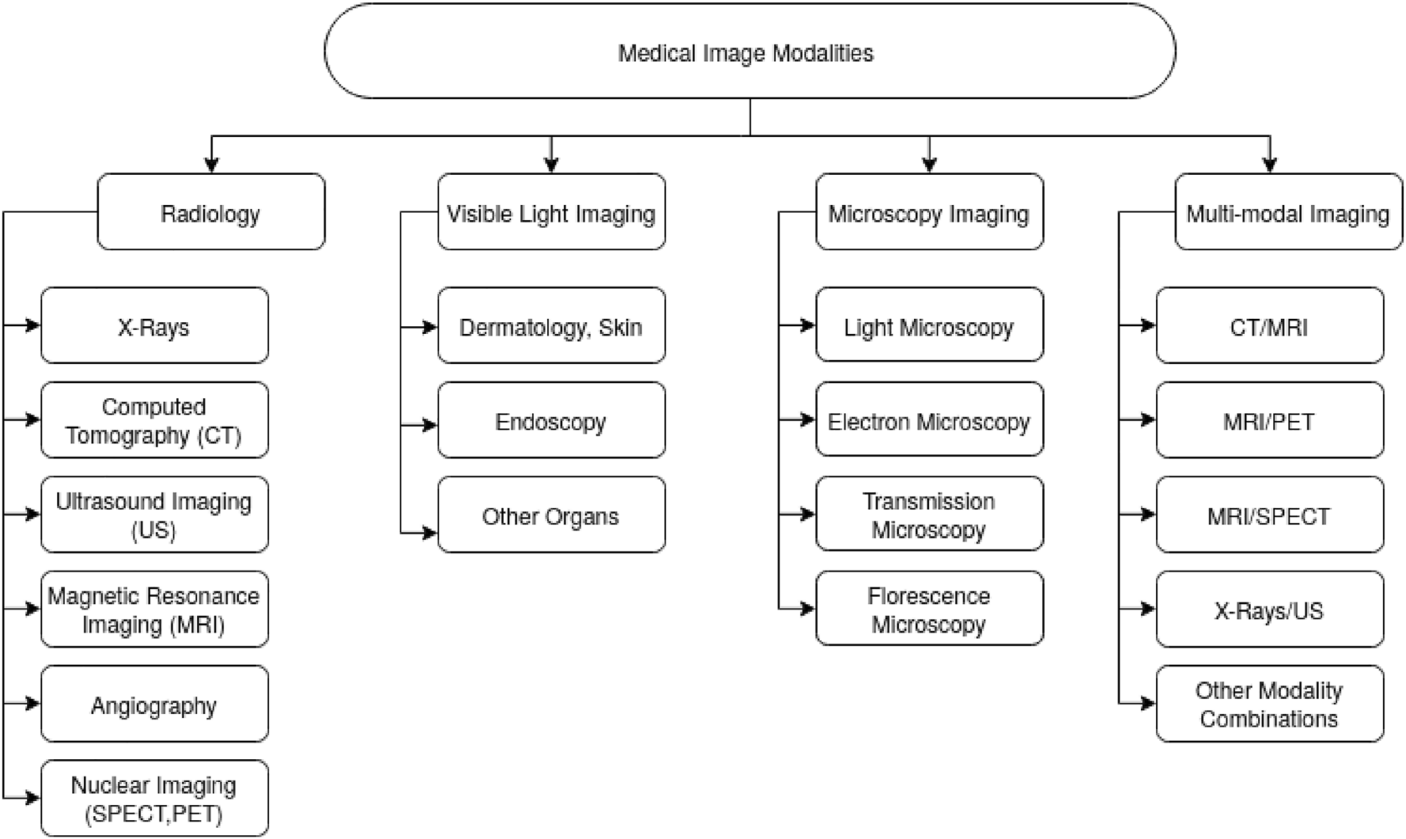
Medical imaging modalities classification

The remaining paper is organized as follows. Section 1 describes the history of artificial intelligence and usage in CAD. Section 2 explain the medical imaging modalities. In Sect. 3, we explain the development of Computer Aided Diagnosis (CAD) and usage and Sect. 4 elaborate the selection criteria of Literature review. In Sect. 5, we explain the deep learning in depth and further we identify the major CNN model in Sect. 6. In Sect. 7, we explain the major development in medical image analyses using deep learning and Sect. 8 elaborate the anatomical structure of medical images. Further, in Sect. 9, we explain the deep learning model development for medical image analysis and Sect. 10 depict the basic tool used in deep learning. Section 11 summarized the extensive survey.

## Medical Imaging Modalities

Each imaging technique in the healthcare profession has particular data and features. As illustrated in Table 1 and Fig. 1, the various electromagnetic (EM) scanning techniques utilized for monitoring and diagnosing various disorders of the individual anatomy span the whole spectrum. Each scanning technique uses a distinct frequency and wavelength and exhibits various qualities [8]. EM waves are dispersed, mirrored, or received by an item whenever they come into contact with it. The magnetic field produced by Magnetic Resonance Imaging causes the body’s natural protons to coordinate. When a radio-frequency signal is delivered to the individual, protons are triggered and begin to swirl at an angle to the magnetic force. The greater region of 3 × 1016 to 3 × 1019 Hz of Computed Tomography and X-ray imaging is extremely radiation and detrimental to public health. Gamma rays are used in nuclear imaging techniques like Positron Emission Tomography and Single Photon Emission Computed Tomography to diagnose biological functions in a person’s tissues. The frequency of gamma rays is larger than 1019 Hz, and their wavelength is fewer than 10 picometers. Magnetic Resonance Image and Ultrasound are governed by the non-ionization concept, whereas X-rays, Single Photon Emission Computed Tomography, Computed Tomography, and Positron Emission Tomography are dependent on the ionization theory [9].

**Table 1:** The most popular and prominent medical imaging modalities are compared

Modalities	Invasive/non-invasive	Details
X-Rays	Non-invasive	The primary method of medical scanning was radiography, which was made possible by Sir Wilhelm Roentgen’s discovery of radiographs on November 8, 1895. The first radiography representation of the human anatomy was also created by Roentgen. Radiology, which is another name for radiology, established the discipline and given rise directly to radiologists, healthcare practitioners with a focus on analyzing medical images. Anatomical diagnoses of the biological anatomy are made using X-rays. Data from X-rays make it simple to identify healthy, irregular, and shattered bones [10]
Computed tomography	Non-Invasive	Midway through the 1970 s, computed tomography (CT) became widely used in hospitals and was the first medical screening tool that machines recognized. By rotating the X-beam tube all over person and directing X-beams across the person at different locations, the CT image is produced. In order to obtain data on transmissions presentation, at most one straight identifier group positions itself in before of the X-beam emitter. A machine combines the numerous data points amassed in this way to create a tomographic representation of the individual. The word ‘tomography’ refers to a precise image of a slice (tomo). The transmission breakthrough known as CT can produce images of various cell regions within the person’s body. Although CT scans may not reveal a lot of information regarding fatty tissue, they do reveal the cranium, bone formation, significant anomalies, infarction, haemorrhage, and tumors in the brain [11, 12]
Magnetic resonance image	Non-invasive	MRI scans provide excellent information regarding blood circulation and delicate and diseased neural processes. Although MRI can detect minor lesions and strokes, it cannot detect brainwave activity or tumor cells communication [11, 12]. The magnetic flux that MRI Imaging systems employ has a magnitude that ranges from 10,000 to 60,000 roughly that of the earth’s magnetic field17. The majority of magnetic resonance imaging employ the highly expressed hydrogen nucleus, or protons, in biological materials for their nuclear magnetic resonance features (about 1018 protons per cubic millimeter of tissue). There is a magnetic charge in particles. Protons typically accept electrons from radio signals with a resonant frequencies of 63 MHZ while positioned in a 1.5 Tesla (T) magnetic flux. 18. The individual is positioned in a magnetic flux during magnetic resonance imaging, and a transmitter produces radio waves
Ultrasound	Non-invasive	The wavelength bands of sound energy used by the US extend from around 20 kHz to many GHz. In comparison to X-rays and CT scans, it is less expensive and better. Both operational and anatomical internal components are covered by the US [13–15]. Whenever a paperweight hits the ground, the impact sends forth pressure waves-also known as sound-that may be perceived from a range. Using specific intensity (“ultra”) sonic, mechanical power can be employed to create images of the participant’s body. Short sound waves are produced when the ultrasonic transmitter through is body interaction with the cell being imaged. Echoes are created when sound frequencies move through the person’s cells and are mirrored by its physical structure. The transmitter then receives the sound radiation that was bounced, and it captures the sound stream that was returned. Pulse echo imaging is the name for the way that ultrasound technology operates in this manner [16]
Positron emission tomography	Invasive	PET scans show neural movement as well as glucose metabolism used as brain power. Using various hues, they also provide details about brain tumor cells [11, 12]
Single photon emission computed tomography	Invasive	By examining blood circulation, SPECT images reveal the section of the brain that is most engaged. The use of SPECT can identify conditions such as dementia, epilepsy, choked blood arteries, etc [11, 12]. Gamma rays are used in the nuclear medicine tomographic imaging technology known as single-photon emission computed tomography. While using a gamma sensor, it is extremely comparable to traditional nuclear medicine planar imaging and can produce full 3D data

## Computer Aided Diagnosis

Despite an increase in CAD development, relatively several CAD solutions are commonly employed in clinical settings. One of the main causes might be that CAD solutions created using traditional machine learning techniques may not have attained the excellent productivity needed to satisfy radiologist and medical practitioners demands for increasing both diagnosis reliability and operational effectiveness. Given the development of deep learning in numerous computer vision and Artificial Intelligence (AI) based systems over the previous few decades, including text and signal processing, face identification, driverless cars, board games and go, there are unrealistic hopes that deep learning will lead to an innovation in CAD effectiveness and mass adoption of deep-learning-based CAD or AI for a variety of activities in the individual treatment plan. The passion has inspired a large number of deep learning projects and articles in CAD. In this section, we’ll talk about a number of problems and obstacles that have arisen while trying to design CAD for diagnostic imaging that is founded on deep learning, as well as the things that should be taken into account when doing so.

The development of CAD based applications uses techniques for conventional machine learning. Image processing techniques were utilized in the traditional machine learning methodology to CAD in diagnostic imaging to identify abnormalities of cancer and discriminate between several categories of features, such as healthy or aberrant, cancerous or mild, on the images. Depending on subject matter expertise, CAD designers create image analysis and separation of features algorithms to describe the image features that may differentiate between the numerous states. The competence of the theoretical conceptions or practical image processing approaches that are meant to convert the image features to quantitative data, as well as the subject experience of the CAD designers, are frequently factors that affect how well the feature classifiers perform. A method employs the extracted features as incoming response variable and a forecast strategy is created by varying the weights of the different characteristics based on numerical characteristics of a series of training instances to determine the likelihood that a image corresponds to a particular categories. Although if they have observed a plethora of cases from the user community, the individual programmer might not be capable of converting the complicated illness trends into a limited amount of feature descriptors using the traditional machine learning technique. The hand-engineered characteristics could also have trouble standing up to the public’s wide range of ordinary and atypical behaviors. The generated CAD system frequently performs poorly in terms of classifier or generalization, leading to a large percentage of wrongful convictions at great sensitivity or conversely.

In several domains, deep learning has become the cutting-edge machine learning technique. Deep learning is a kind of representation learning technique that employs a sophisticated multi-layer neural network topology autonomously trains data interpretations by abstracting the raw data into several layers. Deep convolutional neural networks (DCNN) represent the most widely utilised deep learning systems for sequence identification applications in images. By continuously modifying its parameters through training algorithm, DCNN may be taught to autonomously retrieve pertinent features from the training instances for a specific job. CNN model does not demand explicitly generated features as input because feature representations are discovered during training. The DCNN features are anticipated to outperform hand-engineered features in terms of selectivity and in-variance if adequately trained using a sizable training set that is representative of the population of interest. Deep learning will rapidly examine dozens or hundreds of examples that even individual specialists would not be capable of seeing and memories in their lifetimes since the training procedure is mechanized. As provided as the training dataset is big and diversified sufficient for it to evaluate, deep learning can consequently be highly resilient to the vast scope of changes in characteristics across distinct groups to be discriminated [17]. Machine learning algorithms are well-known to discover and learn the relationship between data and explore to retrieve hidden information from the data. Machine learning have influential techniques and methods that can learn from previous actions. The machine learning algorithms observe and interact with environment to improve the efficacy of objective functions.

Image recognition, tracking and identification is an essential research area in machine learning that is used in a wide range of technologies such as gesture identification, driver-less cars, medical assessment such as medical image processing, tumor cell categorization, imagegrammetry and so on. Object spotting is the task of locating many objects in images and recognizing their locations and categorization. Object identification is regarded to be one of the challenging tasks in the area of machine sensing since the appearance of objects varies substantially based on a range of factors [18, 19].

All machine learning technologies and associated artificial intelligence (AI) models, medical evidence and image analysis may offer the highest opportunity for creating a significant, long-term impact on individual experiences in a relatively short period of time [17]. Image searching, production, computer vision and image-based visualization are all components of the software evaluation and interpretation of medical images [20]. In numerous dimensions, medical image analysis has evolved to encompass image preprocessing techniques, pattern matching, data mining especially image based dataset and deep learning [21]. Deep learning is a popular way for determining the correctness of the future situation. This offered up additional possibilities for interpretation of medical images. Computational intelligence approaches in healthcare handle a diverse range of concerns, from early diagnosis to infection tracking to individualized therapy recommendations. A vast amount of material is now available to clinicians via multiple media platforms such as mri scans, genetic sequencing and pathological imaging [22]. To convert each of this input into valuable advice, the particularly related utilized for patient data include X-ray, Ultrasound, Positron Emission Tomography (PET), Magnetic Resonance Imaging (MRI) and Computed Tomography (CT) [23, 24]. Deep learning is the process of discovering trends in complex objects by utilizing neural network models comprised of multiple convolution layers (comprising several nodes) of artificial neurons [25]. An artificial neuron is a sort of cell that act like a real brain, accepts numerous input images, does a computation and then delivers the optimal outcome [26–28]. This straightforward approach uses a nonlinear activation function before a linear input pattern matching form [26]. Numerous often deployed nonlinear activation functions of a system include the sigmoid transition, ReLU and their variations and tanh (hyperbolic tangent) [29–31].

In recent years, advances in health science and image processing have been made thanks to deep learning algorithms [26, 32–34]. Deep learning techniques using CNNs and computational techniques, in especially, execute better when analyzing large amounts of data, and this has received a lot of study interest. Recent studies imply that the application of deep-learning-based computer-aided identification in medical practice can dramatically decrease the amount of time needed to analyze films and increase diagnosing effectiveness [35, 36].

For healthcare system, numerous research have explored CADe/CADx technology [37–39]. Studies that have already been published examined CADe/CADx methods for medical images using deep learning algorithms [40, 41]. All of these investigations, meanwhile, are missing explanations of the imaging procedures diagnostic procedures and supporting documentation used in the different engineering solutions. Additionally, released assessments must offer a much more thorough assessment of contemporary literature. As a result, we thoroughly review the most recent state-of-the-art implementations of cutting-edge CADe/CADx ideas that address medical images using CNN deep learning algorithms and computational techniques. Due to the dearth of papers using additional models from deep learning in CADe/CADx, we did not include them. We also creatively divide the four phases in the conventional CADe/CADx of medical images into two phases by gaining knowledge from the diagnostic imaging methodology in order to straightforwardly relate their pivotal technology superior properties, taking into account that the primary technology components of these research findings lie in various medical steps. By discussing the implementation, benefits, and drawbacks of CNN in the identification of medical images, as well as potential approaches for investigators to address these challenges, we may indicate the path of future study in this area and potentially other healthcare domains.

## Literature Review Selection Criteria

For this analysis benchmark, there were four steps: (1) keyword search queries in the IEEE Xplore, ACM Digital Library, Scopus, Google Scholar, Science Direct, PubMed, and Web of Science libraries and databases; (2) gathering relevant things and eliminating multiple copies; (3) choosing the benchmark of use; only the lung nodule detection technology based on CT image deep learning was maintained; and (4) evaluating detection systems utilizing the established metrics. The database search terms and logical expressions that we used are listed below: ‘deep learning’ or ‘deep convolutional neural network’ or ‘convolutional neural network’ or ‘CNN’ or ‘DCNN’ and ‘healthcare’ or ‘health-care’ or ‘health care’ or ‘medical’ or ‘clinical’ or ‘image’ or ‘images’ or ‘brain’ or ‘brain injury’ or ‘head’ or ‘head injury’ or ‘skin’ or ‘breast’ or ‘chest’ or ‘pulmonary’ or ‘lung’ or ‘lungs’, and ‘nodule’ or ‘nodules’ or ‘tumor’ or ‘tumors’ or ‘cancer’ and ‘detection’ or ‘detect’ or ‘detected’ or ‘detecting’ or ‘computer-aided detection (CADe)’or ‘computer-aided diagnosis (CADx)’ or ‘CAD’ or ‘CADx’ or ‘CAD’ or ‘CADe’ and ‘histology’ or ‘histopathology’ or ‘histopathological’ or ‘X-ray’ or ‘Xray’ or ‘CXR’ or ‘MRI’ or ‘Magnetic Resonance Imaging’ or ‘Computed Tomography Scan’ or ‘CT Scan’ or ‘CT-Scan’ or ‘Computed Tomography’ or ‘CT’.

## Deep Learning Methodologies

Deep learning-based medical image segmentation is a popular topic in image classification, registration, segmentation and tumor detection research and has great use in the medical field. Deep learning technology can improve computer-aided diagnosis accuracy and efficacy while also easing resource constraints in healthcare, decreasing doctor stress, and reducing reliance on expert knowledge. An overview of some of the most well-known deep learning frameworks is provided below.

The purpose of this part is to formally introduce and define the deep learning ideas, methods, and frameworks that we discovered in the articles on medical image interpretation that were analyzed for this study.

### Neural Network

The fact that neural networks are generic function capable of approximating, or that they are able estimate any mathematical expression to any degree of precision, is a crucial characteristic of neural networks. In other sense, if any process-whether biological or not-can be conceptualized as a function of a collection of variables, then that behavior may be simulated to any unlimited amount of precision, constrained only by the magnitude or complication of the system. Although the aforementioned concept of general approximation is not theoretically precise, it does illustrate one factor that has contributed to the long-standing curiosity in neural networks. This assurance, however, does not offer a mechanism to identify the neural network model’s ideal characteristics that will yield the closest estimation for a particular dataset. Additionally, there is no assurance that the system will deliver precise forecasts for fresh data. All artificial neural models’ underlying components are synthetic neurons. A mathematical expression that specifically translates sources into outcomes is all that constitutes an artificial neuron. Any amount of input numbers are accepted by a single artificial neuron, which then processes them using a particular mathematical expression to produce an outcome [42].

A network system which is used in machine learning is known as Neural Network. It took inspiration from human brain and works similar to human brain. The network architecture of Neural Network is made up of Artificial Neurons. It is a network that has weights on it, you can adjust those weights so that it can learn from it. A neural network has a number of layers which groups the number of neurons together. Each of them has its own function. Network’s complexity depends on the number of layers. That is why the Neural Network is also known as multi-layer perceptron. There are three types of neural network layers. (1) Input Layer, (2) Hidden Layer and (3) Output Layer. Each of them has its own specific purpose. These layers are made up of nodes and each of them has its own domain of knowledge. Neural Networks are highly efficient because they can learn very quickly.

### Multi-layer Perceptrons

An neural network model’s another very basic structure is layer upon layer of densely integrated and interconnected neurons. In this structure, a set quantity of “input neurons” stand in for the input feature values that are determined from the records and transmitted to the sub net, and each linkage among a couple of neurons stands in for one learnable weight parameter. Artificial neural learning refers to the process of maximizing these components, which are the primary variables that may be changed in a neural network [43–45].

The network’s ultimate results are represented by a quantity of output nodes at the opposite end of the network. When properly set up, a network of this kind may be used to create hierarchical, sophisticated judgments regarding the input since each neuron in a particular layer obtains information from every neuron in the layer below. The earliest networks helpful for bioinformatics implementation were layers of neurons arranged in this straightforward manner; these layers are sometimes referred to as “multilayer perceptrons.”

### Feed Forward Neural Networks

The earliest and most fundamental model of a neuron is the perceptron. A group of inputs are taken, added, and then an activation function is applied before sending the results to the output layer. A fundamental class of neural networks is called a feed forward neural network (FFNN). The intermediate levels are buried, with the input layer at the top and the output layer at the bottom. There is no feedback in the entire network as the signal propagates unidirectional from the input layer to the output layer [46–48].

Multilayer perceptrons is another name for Feed Forward Neural Network depicted in Eq. 1.

1 $$\begin{aligned} y_{l} = \sum _{i=0}^{n}(W_{i} * X_{i} ) + b \end{aligned}$$

### Recurrent Neural Networks (RNNs)

When processing data with time series characteristics, RNN excels. It can also help with data analysis and mining for information about time series features and semantic change. The weight of the single unit that makes up the RNN layer is shared. Each training sample example will only go through one unit in the state of the various time series, after which its weight will be changed continuously [49–51].

#### Long-Short Term Memory (LSTM)

The vanishing/exploding gradient issue is solved with gates and an explicitly designated memory cell in Long-Short Term Memory (LSTM) networks. The main driving force behind these is electronics, not biology. Input, output, and forget gates, together with a memory cell, are all present in every neuron. These gates’ function is to safeguard information by controlling the flow of it or blocking it [52–54]. The Eq. 2 show the LSTM’s feedforward calculation procedure in more detail.

2 $$\begin{aligned} \begin{aligned}\begin{aligned} {\textbf{I}}_t&= \sigma ({\textbf{X}}_t {\textbf{W}}_{xi} + {\textbf{H}}_{t-1} {\textbf{W}}_{hi} + {\textbf{b}}_i),\\ {\textbf{F}}_t&= \sigma ({\textbf{X}}_t {\textbf{W}}_{xf} + {\textbf{H}}_{t-1} {\textbf{W}}_{hf} + {\textbf{b}}_f),\\ {\textbf{O}}_t&= \sigma ({\textbf{X}}_t {\textbf{W}}_{xo} + {\textbf{H}}_{t-1} {\textbf{W}}_{ho} + {\textbf{b}}_o),\\ \tilde{{\textbf{C}}}_t&= \text {tanh}({\textbf{X}}_t {\textbf{W}}_{xc} + {\textbf{H}}_{t-1} {\textbf{W}}_{hc} + {\textbf{b}}_c), \end{aligned}\end{aligned} \end{aligned}$$

Among them, $W_{hi}$, $W_{hf}$, $W_{ho}$, $W_{hi}$ represent the matrix parameters related to the input and 3 Gates, and then $b_{i}$, $b_{f}$, $b_{o}$, $b_{c}$ represent the bias parameters related to the input and the three Gates, represents the Sigmoid function, and finally, 0 represents the same position of the two vectors Elements are multiplied together.

#### Gated Recurrent Units (GRU)

Although the gradient problem in RNN can be greatly reduced by LSTM, a single LSTM unit has four times as many parameters as a single RNN unit due to the addition of three extra Gates. Greater parameters necessitate more computing power. Although it has fewer parameters than LSTM because it lacks an output gate, GRU is similar to LSTM with forget gate [55].

A very powerful LSTM neural network variant is the GRU. GRU makes the structure shallower and computationally less expensive while maintaining the LSTM effect. It is also the most used type of neural network at the moment because it has a simpler structure and better results than an LSTM network. Due to the fact that GRU is a variation of LSTM, it can alleviate the lengthy reliance issue in RNN [56]. The GRU feedforward calculation is displayed in Eq. 3.

3 $$\begin{aligned} \begin{aligned}\begin{aligned} {\textbf{R}}_t = \sigma ({\textbf{X}}_t {\textbf{W}}_{xr} + {\textbf{H}}_{t-1} {\textbf{W}}_{hr} + {\textbf{b}}_r),\\ {\textbf{Z}}_t = \sigma ({\textbf{X}}_t {\textbf{W}}_{xz} + {\textbf{H}}_{t-1} {\textbf{W}}_{hz} + {\textbf{b}}_z), \end{aligned}\end{aligned} \end{aligned}$$

${\textbf{W}}_{xr}, {\textbf{W}}_{xz} \in {\mathbb {R}}^{d \times h}$ represent the input training parameters and ${\textbf{W}}_{hr}, {\textbf{W}}_{hz} \in {\mathbb {R}}^{h \times h}$ are bias parameters, and the two Multiply elements of the same position of the vector, respectively.

#### Bidirectional Recurrent Neural Networks

Bidirectional Long Short-Term Memory Networks (BiLSTM), Bidirectional Gated Recurrent Units (BiGRU), and Bidirectional Recurrent Neural Networks (BiRNN) all resemble their unidirectional counterparts in appearance. On the other hand, conventional RNNs cannot process data for the future and can only process input in one direction. The forward, backward, and output Eq. 4 indicate how these bidirectional networks can extract complete temporal information at time t by combining past and future data, improving the model’s performance on sequence issues [57, 58].

4 $$\begin{aligned} \begin{aligned}\begin{aligned} \overrightarrow{{\textbf{H}}}_t&= \phi ({\textbf{X}}_t {\textbf{W}}_{xh}^{(f)} + \overrightarrow{{\textbf{H}}}_{t-1} {\textbf{W}}_{hh}^{(f)} + {\textbf{b}}_h^{(f)}),\\ \overleftarrow{{\textbf{H}}}_t&= \phi ({\textbf{X}}_t {\textbf{W}}_{xh}^{(b)} + \overleftarrow{{\textbf{H}}}_{t+1} {\textbf{W}}_{hh}^{(b)} + {\textbf{b}}_h^{(b)}),\\ {\textbf{O}}_t&= {\textbf{H}}_t {\textbf{W}}_{hq} + {\textbf{b}}_q. \end{aligned}\end{aligned} \end{aligned}$$

### Unsupervised Models

Unsupervised learning research’s major objective is to pre-train a deep learning model (also known as a “discriminator” or “encoder”) that will be utilized for many other challenges. The encoder characteristics must be broad sufficient to be applied to classification techniques, such as training on ImageNet and producing outcomes that are as near to supervised models as feasible [59].

As of right now, supervised models consistently outperform unsupervised trained models. This is to ensure that the model can more effectively incorporate the dataset’s features thanks to the supervision. However, if the model is subsequently extended to other activities, monitoring may likewise become less effective. Unsupervised training is hoped to be able to offer more universal characteristics for learning to complete any work in this respect [60, 61].

#### Autoencoders (AEs)

In that they are an adaptation of FFNNs rather than a fundamentally unique design, autoencoders (AEs) are similar to FFNNs in this regard. The only thing to keep in mind is that the number of input characteristics (number of neurons) in the input layer and the output layer should match (the number of neurons). Check to see if the input and output are both equal. The basic idea behind autoencoders is to automatically compress data rather than encrypt it, hence the name. The entire network has hidden layers that are thinner than the input and output layers and is structured like an hourglass [62, 63]. The Variational Autoencoders (VAEs) is depicted in equation ??.

5 $${\partial(q)} = E_{zq({z}\vert{x})}(\lg p({x}\vert{z})) - KL(q({z}\vert{x})||p(z))$$

A unique network called Sparse Autoencoders (SAEs) adds sparsity to the network. Sparse Autoencoder wants to use the original data to create a low-dimensional representation. The preparation of the image typically involves sparse automatic coding to decrease the size of the data and retrieve possibly useful information.

#### Boltzmann Machine (BM)

Unbalanced connections make up a Boltzmann machine. In terms of graph theory, it is a full graph. Any device is linked. Neurons and other components will make the decision to switch on or off. Initially, BM was solely employed to refer to models that contained just binary variables. The limited Boltzmann machine primarily adds “restriction” as compared to the Boltzmann machine. To make the complete graph a bipartite graph is the alleged restriction. In in addition to other things, restricted Boltzmann machines may be employed to automatically learn (hidden state outcomes are features), develop deep belief networks, and decrease complexity (fewer hidden layers) [64, 65].

#### Deep Belief Network (DBN)

Multi-layer Restricted Bolt Machine (RBM) based neural networks are called Deep Belief Networks (DBN). It can be categorized as either a discriminative or generative model. To put on weight, the training strategy adopts the unsupervised hungry layer-by-layer approach. Deep Belief Network learning has been finished layer by layer. After being employed to estimate the hidden layer in each level, the array of objects is then utilised as the data vector for the following (higher) layer. Numerous Restricted BMs must be “connected in series” to form a DBN, with the outcome of one BM serving as the intake of the following and the hidden layer of the first BM serving as the feature map of the second. During the learning phase, it is essential to thoroughly train the Restricted BM of the former layer before learning the Restricted BM of the current layer to the previous layer. Hua et al.[66, 67].

#### Generative Adversarial Network (GAN)

Generative Adversarial Networks (GANs) are essentially a training mode and not a final network structure. The fundamental tenet of Generative Adversarial Network is that the discriminator and generator work in tandem. The discriminator must attempt to differentiate among real samples (such as real pictures) and fake samples produced by the generator, or “Fake images.” The ideal competitive environment is one where both sides continuously improve, where the capability to differentiate between them becomes increasingly stronger, and where the capacity to produce deception becomes greater as well. The outcome of the opposition is unimportant. What matters most in the conclusion is the generator’s capacity to produce instances that are sufficiently equivalent to the real instances, resemble the sample data in the training set, and have a dispersion that is substantially close to that of the training examples distribution. Xin et al. [68, 69]. Define the objective function that GAN must optimize, which is represented by the expression in Eq. 6.

6 $$\begin{aligned} \min _D \{ - y \log D({\textbf{x}}) - (1-y)\log (1-D({\textbf{x}})) \}, \end{aligned}$$

### Convolutional Neural Networks (CNNs)

An uncommon variety of neural network is called a convolutional neural network (CNN) or a deep convolutional neural network (DCNN). They can be used for other types of input, such as audio, though image processing is where they are most frequently used. Convolutional neural networks (CNNs/ConvNets) use neurons with biases and weights that can be learned. The classification score is generated after each neuron computes the dot product using the data it has been given. By incorporating specific characteristics into the network structure using CNN, we can improve the effectiveness of the feedforward function and pass parameters [70]. People who share reduce many variables. Equation 7 explain the input and output capabilities of the CNN as well as the network’s overall non linearity and cost function are depicted in Eqs. 8 and 9 respectively.

7 $$\begin{aligned}{} & {} x_{ij}^\ell = \sum _{a=0}^{m-1} \sum _{b=0}^{m-1} \omega _{ab} y_{(i+a)(j+b)}^{\ell - 1} \end{aligned}$$

8 $$\begin{aligned}{} & {} y_{ij}^\ell = \sigma (x_{ij}^\ell ). \end{aligned}$$

9 $$\begin{aligned}{} & {} \frac{\partial {E}}{{\omega _{ab}}} = \sum _{i=0}^{N-m}\sum _{j=0}^{N-m} \frac{\partial {E}}{\partial {x_{ij}^\ell }} \frac{\partial {x_{ij}^\ell }}{\partial {\omega _{ab}}} = \sum _{i=0}^{N-m}\sum _{j=0}^{N-m} \frac{\partial {E}}{{x_{ij}^\ell }} y_{(i+a)(j+b)}^{\ell -1} \end{aligned}$$

### Convolutional Layer

Convolutional Layer is a first layer of Convolutional Neural Network. This layer consists of sets of Filters or Kernel. Their job is to use a Convolutional operation to the input and passing the result to the succeeding layer. The filter takes a subset of the input data. The territorial relationship between pixels by learning image options using tiny squares of input data ensures by this layer. The Convolutional layer’s vector is the final output. This layer performs linear multiplications with the goal of extracting the input image’s high-level characteristics as a convolution operational activity. Due to the linear nature of the convolutional process at this layer, the final size is also produced by layering the activation maps of all filters on the depth dimension. Similar to an old neural network, linear operation primarily entails the multiplication of weights with the input [71, 72].

### Deconvolution

Deconvolution also known as transposed convolution is a mathematical operation by which the effect of convolution reverses. It is exactly the multivariate Convolutional function’s inverse. For example, giving input to the Convolutional layer and getting output then give the same output to deconvolutional layer can get you to the same input you given first. Deconvolution is just to reverse the input back to large size [73, 74].

### Dilated Convolution (Atrous Convolution)

A type of convolution by which the kernel inflates by inserting holes between the kernel elements. Another parameter to the Convolutional layers has been introduced by dilated convolution called the dilation rate. Additional parameter of dilation rate as indicates that how much the kernel is expended. It’s targeted to increase the size of reception field by avoiding the increase in parameter sizes. There are normally spaces inserted between the elements of kernel [75].

### Striding

Striding defines the step size of the kernel while sliding through the image. Stride of one defines that the kernel slides through the image pixel by pixel. Stride of two defines that the kernel slides through image by moving 2 pixels per step i.e., skipping of 1 pixel. Stride ($\ge 2$) can be used for down-sampling an image [76].

### Padding

How the border of an image is handled is defined by padding. Spatial output dimensions kept equal to the input image by padded convolution, by padding 0 around the input boundaries (if necessary). On the other side, without adding 0 around the input boundaries, unpadded convolution only perform convolution on the pixels of the input image [77]. The size of output is smaller than the input size. For an input image, size i, kernel size k, padding p and stride s, from convolution the output image has size o:

10 $$\begin{aligned} o = lower\_bound ((i + 2p -- k)/s) + 1 \end{aligned}$$

### Pooling Layer

A new layer added after the Convolutional layer is a pooling layer. Specially, after the Convolutional layer applies a nonlinearity to the feature maps output. The inclusion of pooling layer right after the Convolutional layer is a usual pattern used in the ordering of layers in a convolutional neural network and can be repeated once or more then once in a given model. The pooling layer separately operates upon each feature map so that it can create a new set of pooled feature maps of same number. Pooling involves selection of pooling operation just like a filter going to be applied on feature maps. Size of pooling filter or operation is smaller in size than the size of the feature map. Mainly, it is always 2 × 2 pixels applied with a 2 pixels stride. It means that pooling layer always going to reduce the size by a factor of 2 of each feature map [78]. Two of the common functions used in pooling operation are given below:

1. *Average Pooling* A convolutional neural network’s layers repeatedly apply learnt filters to input images to produce feature maps that list the features present in the image. For each region on the feature map, determine the average value. In order to construct a down-sampled (pooled) feature map, the average value for regions of a feature map is calculated using the average pooling method. It is frequently applied following a Convolutional layer. It provides a tiny bit of translation in-variance, which means that changing the image’s size slightly has little impact on the numbers of the majority of pooled outcomes. Max Pooling collects higher obvious characteristics like edges, although it collects characteristics more uniformly.
2. *Maximum Pooling* A feature map is down-sampled (pooled) by using the Max Pooling pooling procedure, which determines the highest value for regions of the feature map. It is frequently used following a Convolutional layer. It adds a tiny bit of translation in-variance, which means that changing the image’s size slightly has little impact on the numbers of the majority of pooled outcomes [79].

### Fully Connected Layer

Fully connected layer is the very important component of the neural networks. It is very successful in the field of recognizing and classifying the images. Fully connected layer is the classic neural network architecture. Fully connected layers are those layers where all the inputs from one layer is connected to every activation unit of the next layer. In this layer, all the input units have a separable weight to each output unit. There are two of fully connected layer one is for input and other is for output. The fully connected input layer flattens the input and change it into the vector for the input of next stage. Then the next stage analyzes it and apply weight to project the right label. Then the fully connected output provides the expected labels to each label [80].

### Activation Functions

Mathematical equations that determine the output of a neural network are known as activation functions. The function is attached with each and every neuron in the network. It determines whether it should be fired (activated) or not based on whether the input of each neuron is relevant for the model’s prediction. Activation functions are also help normalize the output of each neuron to a specific range between -1 and 1 or between 1 and 0. Activation functions must be computationally efficient because for each data sample they are calculated across thousands or millions of neurons. Modern day neural networks use a backpropagation technique to train the model which places increased computational strain on activation function and its derivative function [81].

### Batch Normalization

Batch normalization is also known as batch norm. It’s a layer which allows each and every layer of network to do learning more independently. Its use is to normalize the previous layers output. The input layer is scaled by the activation in normalization. By using batch normalization, learning becomes more efficient. Moreover, to avoid over-fitting of a model it can be used as a regularization. To standardize the input or the outputs, the layer is added to the sequential model. It can be used at some several points in between layers of model. It is placed right after defining sequential model and after the convolution layers and pooling layers [82].

### Dropout

The dropout term relates to “dropping out units” in a neural network. The thing dropping a unit out means removing the unit temporarily from the network and all the connections whether incoming or outgoing all are removed. To prevent neural networks from over-fitting the Dropout method is used. In simple way dropout ignores the units during the time period of training of the system [83].

### Softmax

SoftMax is a function, it is additionally referred as soft argmax or normalized exponential function. This function which is used as the activation function in output layer of the neural network models that predict the multinomial probability distribution. It is a function that changes the K real value into the vector of K real value whose sum is 1. The input value can be anything like positive, negative less than or greater than one, but this function changes the value to 0 and 1, for the probability. If the input is greater than this function will change the value in large probability or if the input is lesser than the function will change the value in small probability, but the value will always lie between 0 or 1 [83].

### Optimizer

Optimizer is the techniques or algorithms which are used to change the attributes of the neural networks. They are used to solve optimization issues by minimizing the function. Optimizer is the one which is used to reduce the losses and to provide us with accurate results [84]. Some strategies are used to initialize the weight and with the period of time it is updated by using the following equation:

11 $$\begin{aligned} W_{new} = W_{old} - lr * (\bigtriangledown W.L)W_{old} \end{aligned}$$

This equation is used to update the weight and to get the accurate results.

### Momentum

Neural network momentum is a simple technique or method which improves accuracy and training speed both. Momentum helps in flattening the variations if there is continuous change in the direction of the gradient. The momentum value is used to avoid the situation of getting stuck in local minima. It is the value which is between 0 and 1. If the value of momentum is greater than the learning is kept smaller. The larger value of the momentum is also considered as that the convergence will occur rapidly. The small value of the momentum cannot avoid local minima and can also result in the delay of systems training [85].

### Learning Rate

Learning rate are the hyper-parameters in the configuration of the neural networks. It used in the training of neural networks that has small positive value. It controls the adaptation of the model to the problem. Learning rate controls how much change in the model is required in response to estimate the error when the values of weights are updated. It is very challenging to choose learning rate because its values are too small which may result in a lengthy training process [86].

## CNN Model Zoo

### LeNet

LeNet architecture is very compact and simplified. LeNet was introduced by Yann LeCunn in 1989 [87]. It is simplified to such an extent that it can be trained on CPU if you do not have any kind of GPU support but if you have any kind of GPU support for your computer, a lot faster results could be achieved. LeNet has many versions which are LeNet-5, LeNet-4, LeNet-1, Boosted LeNet-4. LeNet is used for handwritten words recognition. Mostly it was used with the applications related to MNIST dataset. It consists upon basic CNN parts like pooling layers, Convolutional layers and fully connected layers [88]. (Figs. 2, 3, 4, 5, 6, 7, 8, 9, 10, 11, 12, 13, 14, 15, 16, 17, 18, 19, 20, 21, 22, 23 and 24)

**Fig. 2:**
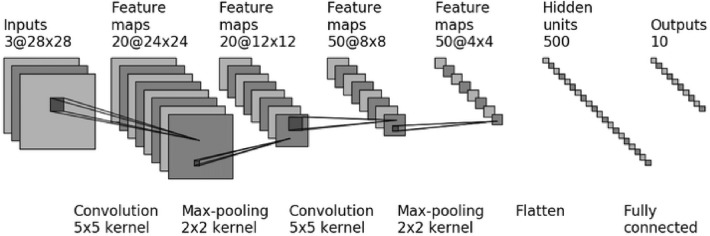
LeNet is a classic convolutional neural network employing the use of convolutions, pooling and fully connected layers. It was used for the handwritten digit recognition task with the MNIST dataset(dataset of 60,000 small square 28 × 28 pixel gray-scale images of handwritten single digits between 0 and 9) [87]

LeNet-5 CNN architecture is made up of 7 layers. The layer composition consists of 3 Convolutional layers, 2 subsampling layers and 2 fully connected layers.

- The first layer is the input layer. In this layer you have a simple digit range from 0-9. It is a 32 × 32 grayscale image and each grayscale image have a digit range from 0-9. You need to classify what digit image has in it. The first you used a Convolutional technique in which you are using a filter size of 5 × 5 and stride is 1. The next layer is of size 28 × 28 × 6 where 6 is the no. of filters you’ve used.
- Next the subsampling technique in which you used a avg pooling where the filter size is 2 × 2 and stride is of 2. The next layer is of size 14 × 14 × 6 where 6 is the no. of filters you’ve used.
- Further they employed Convolutional with 5× 5 filter and stride is of 1. The next layer is of size 10 × 10 × 16 where 16 is the no. of filters you’ve used.
- They applied average pooling where the filter size is 2 × 2 and stride is of 2. The next layer is of size 5 × 5 × 16 where 16 is the no. of filters you’ve used. Here 5 × 5 × 16 means 400 neurons. Number of layers are 16 and each layer have 25 neurons in it.
- For dense connection, they employ fully connection each 400 neurons to first hidden layer having 180 neurons which have 180 × 400 connections.
- These 180 neurons are fully connected to the next hidden layer having 84 neurons size of 84 × 180 connections.
- Finally the output layer will be of 10 × 10 which is fully connected to the previous layer size of 10 × 84 connections.

The main disadvantage of LeNet-5 is at time padding technique is not used to it takes some extra effort to retain the size of the image.

### AlexNet

AlexNet CNN architecture is more complex as compared to the LeNet architecture. AlexNet was introduced by Alex Krizhevsky. In 2012, AlexNet competed in ImageNet Large Scale Visual Recognition Challenge. AlexNet won the competition and was at par then the opponent. AlexNet was able to get 15.3% top-5 error which was 10.8% lower then the opponent. AlexNet requires more depth in its architecture as compare to LeNet. AlexNet consist of pooling layers, Convolutional layers and fully connected hidden layer [89].

**Fig. 3:**
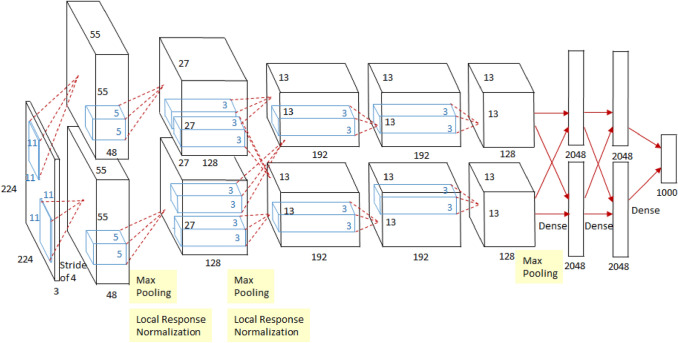
AlexNet is a CNN architecture and its basic building blocks are max pooling and dense layer. It has imageNet classification with the deep learning techniques. This architecture will be use in pre trained model for the object detection in computer vision [89]

AlexNet CNN architecture is made up of 8 layers. The first 5 are Convolutional layers, some of them followed by 3 max pooling layer and last 3 are fully connected layers. It also uses ReLU Nonlinearity, multiple GPU’s (on two parallel GPU’s) and overlapping pooling.

- The first layer is the input layer. It is a 224 × 224 × 3 RGB image. The first you used a Convolutional technique in which you are using a filter size of 11 × 11 and stride is 4 and pool size of 2. The next layer is of size 55 × 55 × 96 where 96 is the no. of filters you’ve used.
- Next the pooling technique in which you used a max pooling where the filter size is 3 × 3 and stride is of 2. The next layer is of size 27 × 27 × 96 where 96 is the no. of filters you’ve used.
- They use same Convolutional with 5 × 5 × 96 filter. The next layer is of size 27 × 27 × 256 which means there are total of 256 filters and each filter have 5 × 5 × 96 filters in it.
- Furthermore, they employ max pooling where the filter size is 3 × 3 and stride is of 2. The next layer is of size 13 × 13 × 296 where 256 is the no. of filters you’ve used.
- They apply same Convolutional with 3 × 3 × 256 filter. The next layer is of size 13 × 13 × 384 which means there are total of 384 filters and each filter have 3 × 3 × 256 filters in it.
- They engage same Convolutional with 3 × 3 × 384 filter. The next layer is of size 13 × 13 × 354 which means there are total of 384 filters and each filter have 3 × 3 × 384 filters in it.
- Next the same Convolutional with 13 × 13 × 384 filter is used. The next layer is of size 13 × 13 × 256 which means there are total of 256 filters and each filter have 3 × 3 × 384 filters in it.
- They enforce max pooling where the filter size is 3 x 3 and stride is of 2. The next layer is of size 6 × 6 × 256. Here 6 × 6 × 256 means 9216 neurons. Number of layers are 256 and each layer have 36 neurons in it.
- For dense connection, they employ fully connect each 9216 neurons to first hidden layer having 4096 neurons which have 4096 × 9216 connections.
- These 4096 neurons are fully connected to the next hidden layer having 4096 neurons size of 4096 × 4096 connections.
- Finally the output layer will be of 1000 neurons which is fully connected to the previous layer size of 100 × 4096 connections.

The main difference between LeNet-5 and AlexNet is that it uses ReLU activation function that will output the input directly if it is positive, otherwise, it will output zero.

### ZfNet

ZfNet CNN is an improvement over the AlexNet. ZfNet was introduced in 2013 in ILSVRC (ImageNet Large Scale Visual Recognition Challenge). In ZfNet the size of Filter is reduced and Convolutional strides are also reduced. The design of ZfNet came across the motivation of visualizing intermediate feature layers and classifiers operation [90].

**Fig. 4:**
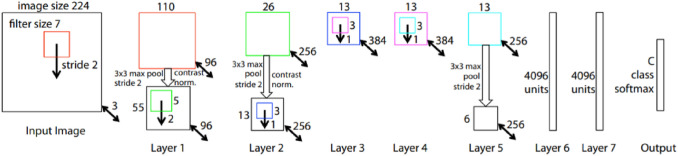
ZfNet is a classic convolutional neural network. The design was motivated by visualizing intermediate feature layers and the operation of the classifier [90]

- The first layer is the input layer. It is a 224 × 224 × 3 RGB image. The first you used a Convolutional technique in which you are using a filter size of 7 × 7  and stride is 2 and pool size of 3. The next layer is of size 55 × 55 × 96 where 96 is the no. of filters you’ve used.
- Used the pooling technique in which you used a max pooling where the filter size is 3 × 3 and stride is of 2. The next layer is of size 27 × 27 × 96 where 96 is the no. of filters you’ve used.
- Next they employ Convolutional with 5 × 5 × 96 filter. The next layer is of size 7 × 7 × 256 which means there are total of 256 filters and each filter have 5 × 5 × 256 filters in it.
- Further, they apply max pooling where the filter size is 3 × 3 and stride is of 2. The next layer is of size 13 × 13 × 256 where 256 is the no. of filters you’ve used.
- They used interactively used same Convolutional with 3 × 3 × 256 filter. The next layer is of size 13 × 13 × 384 which means there are total of 384 filters and each filter have 3 × 3 × 256 filters in it.
- They employ same Convolutional with 3 × 3 × 384 filter. The next layer is of size 13 × 13 × 348 which means there are total of 384 filters and each filter have 3 × 3 × 384 filters in it.
- Further, they employ same Convolutional with 3 × 3 × 384 filter. The next layer is of size 13 × 13 × 256 which means there are total of 256 filters and each filter have 3 × 3 × 384 filters in it.
- They apply max pooling where the filter size is 3x3 and stride is of 2. The next layer is of size 6 × 6 × 256. Here 6 × 6 × 256 means 9216 neurons. Number of layers are 256 and each layer have 36 neurons in it.
- For dense connection, they employ fully connect each 9216 neurons to first hidden layer having 4096 neurons which have 4096 × 9216 connections.
- These 4096 neurons are fully connected to the next hidden layer having 4096 neurons size of 4096 × 4096 connections.
- Finally the output layer will be of 1000 neurons which is fully connected to the previous layer size of 1000 × 9216 connections.

### VGG

The convolutional neural network architecture called the VGG framework, or VGGNet, that covers 16 layers is also known as VGG16. It was developed by A. Zisserman and K. Simonyan from the University of Oxford. The study article titled “Very Deep Convolutional Networks for Large-Scale Image Recognition” contains the framework that these authors released. In ImageNet, the VGG16 model outperforms top-5 accuracy results of about 92.7%. A resource called ImageNet has over 14 million photos that fall into about 1000 categories. It was also among the most well-liked models presented at ILSVRC-2014 [91].

**Fig. 5:**
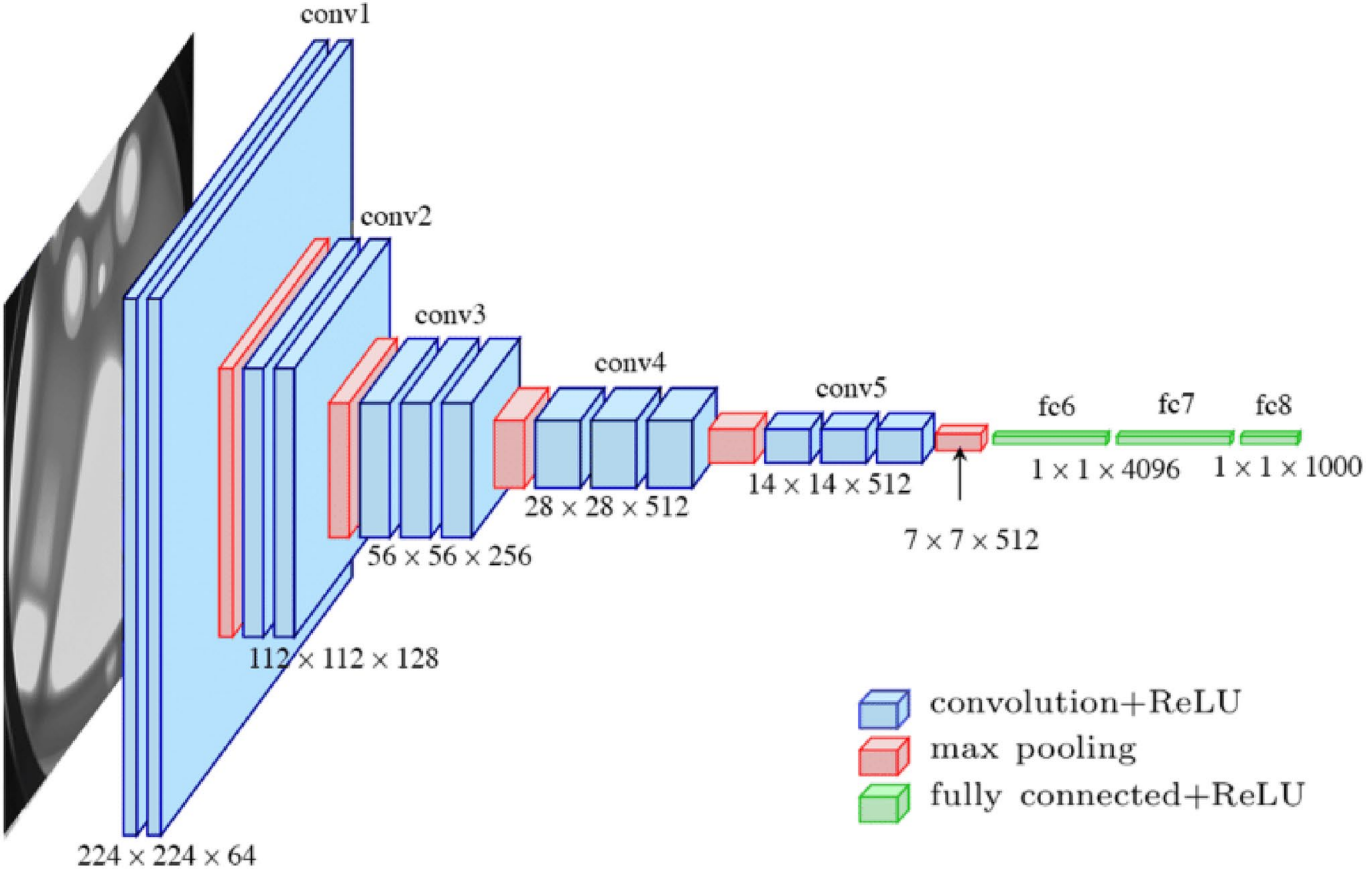
The convolution neural network receives a fixed-size RGB image with a dimension of 224 by 224. The primary preprocessing it performs is removing out of each pixel the average RGB numbers calculated from the training sample. The image is then sent via a series of convolutional (Conv.) layers that contain filters with a tiny 3 × 3 input patch, the smallest number necessary to retain the concepts of up/down, left/right and center portion [91]

VGGNet architecture is made up of 16 layers. The layer composition consists of 13 Convolutional layers, 5 pooling layer and 3 fully connected layers.

- The first layer is the input layer. It is a 224 × 224 × 3 RGB image. The first you used a Convolutional technique in which you are using a filter size of 3 × 3  and stride is 1 and same padding size. You Convolutional this layer 2 times with the same filter size. The next layer is of size 224 × 224 × 64 where 64 is the no. of filters you’ve used.
- Further, they employ the pooling technique in which you used a max pooling where the filter size is 2 × 2 and stride is of 2. The next layer is of size 112 × 112 × 64 where 64 is the no. of filters you’ve used.
- Regularly, they use the same Convolutional filter size of 3 × 3 and stride is 1 and same padding size. Again Convolutional this layer 2 times. The next layer is of size 112 × 112 × 128 which means there are total of 128 filters.
- Repeatedly they use max pooling where the filter size is 2 × 2 and stride is of 2. The next layer is of size 56 × 56 × 128 where 128 is the no. of filters you’ve used.
- They continuously employ the same Convolutional filter size of 3 × 3 and stride is 1 and same padding size. But Convolutional this layer 3 times. The next layer is of size 56 × 56 × 256 which means there are total of 256 filters.
- They use max pooling where the filter size is 2 × 2 and stride is of 2. The next layer is of size 28 × 28 × 256 where 256 is the no. of filters you’ve used.
- Repeatedly use same Convolutional filter size of 3 × 3 and stride is 1 and same padding size. Again Convolutional this layer 3 times. The next layer is of size 28 × 28 × 512 which means there are total of 512 filters.
- Regularly apply max pooling where the filter size is 2 × 2 and stride is of 2. The next layer is of size 14 × 14 × 512 where 512 is the no. of filters you’ve used.
- They employ the same Convolutional filter size of 3 × 3 and stride is 1 and same padding size. Again Convolutional this layer 3 times. The next layer is of size 14 × 14 × 512 which means there are total of 512 filters.
- Use max pooling where the filter size is 2x2 and stride is of 2. The next layer is of size 7 × 7  × 512 where 512 is the no. of filters you’ve used. Here 7 × 7  × 512 means 25088 neurons. Number of layers are 512 and each layer have 49 neurons in it.
- For dense connection, fully connect each 25088 neurons to first hidden layer having 4096 neurons which have 4096 × 25088 connections.
- These 4096 neurons are fully connected to the next hidden layer having 4096 neurons size of 4096 × 4096 connections.
- Finally the output layer will be of 1000 neurons which is fully connected to the previous layer size of 1000 × 4096 connections.

### GoogleNet

GoogleNet was introduced in 2014 by a team at google. GoogleNet was the 2014 winner of ILSVRC (ImageNet Large Scale Visual Recognition Challenge). GoogleNet achieve top-5 error rate of 6.67% which was very close of the error rate of human level. GoogleNet used the convolutional neural network inspired by the LeNet CNN. GoogleNet implemented Inception module in it. GoogleNet used RMSprop, image distortions and batch normalization [92].

**Fig. 6:**
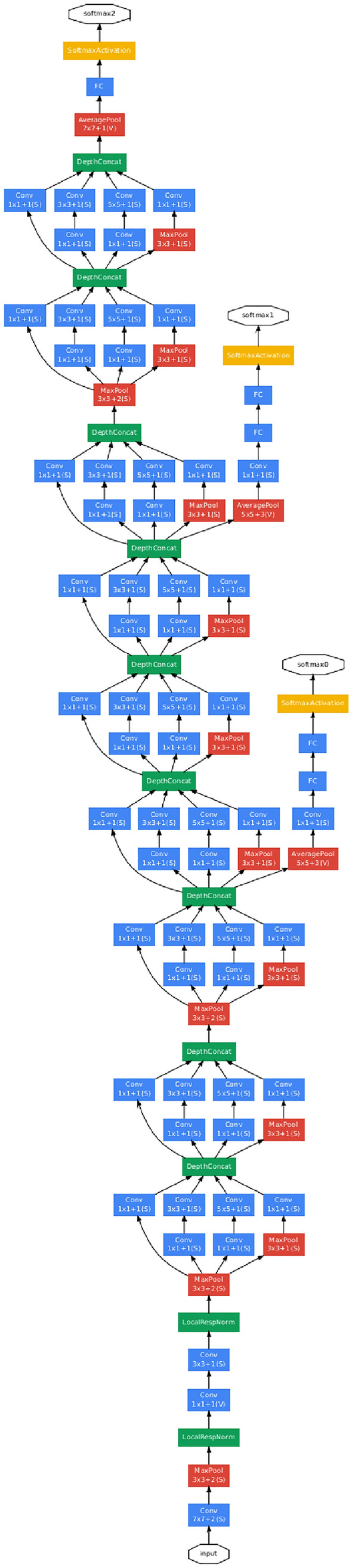
GoogLeNet is a deep CNN and it has a 22-layer architecture and researchers at Googles motivated by visualizing intermediate feature layers and the operation of the classifier [92]

The GoogLeNet architecture consists of 22 layers (27 layers including pooling layers) and part of these layers are a total of 9 inception modules.

- The first layer is the input layer. It is a 224 × 224 × 3 RGB image. The first you used a convolutional technique in which you are using a filter size of 7 × 7 and stride is 2. The next layer is of size 112 × 112 × 64 where 64 is the no. of filters you’ve used.
- Further, they employ the pooling technique in which you used a max pooling where the filter size is 3 × 3 and stride is of 2. The next layer is of size 56 × 56 × 54 where 64 is the no. of filters you’ve used.
- They apply Convolutional with 3 × 3 filter and stride 1. The next layer is of size 56 × 56 × 192 where 192 is the no. of filters you’ve used.
- Frequently apply max pooling where the filter size is 3 × 3 and stride is of 2. The next layer is of size 28 × 28 × 192 where 192 is the no. of filters you’ve used.
- The inception technique is used in which you perform 3 filters (1 × 1),(3 × 3),(5 × 5) and then the max pooling. The next layer will be of size 28 × 28 × 256.
- Regularly they employ the inception technique, the next layer will be of size 28 × 28 × 480.
- They apply max pooling where the filter size is 3 x 3 and stride is of 2. The next layer is of size 14 × 14 × 480 where 480 is the no. of filters you’ve used.
- The inception technique is used 5 times and you have the next layer of size 14 × 14 × 832.
- Further, they apply max pooling where the filter size is 3 × 3 and stride is of 2. The next layer is of size 7 × 7 × 832 where 832 is the no. of filters you’ve used.
- Do the inception 2 ×  times and you found the next layer size of 7 × 7 × 1024.
- Regularly, they apply average pooling with the filter size of 7 × 7 and stride 1. You have the next layer of size 1 × 1 × 1024 and drop the 40% and you have finally 1 × 1 × 100 output.

GoogleNet has the benefit of training more quickly than VGG. Pre-trained GoogleNets are less in size than VGGs. GoogleNet does have a volume of only 96MB, but a VGG model might have > 500 MBs.

### ResNet

ReNet is an application of Keras. ResNet is a short form of Residual Network. It was introduced in 2015 and it changed the research community forever by ground breaking results. There are many version of ResNet and each version is different from other and serve a different purpose. The different version of ResNet are ResNet-18, ResNet-34, ResNet-50, ResNet-101, ResNet-110, ResNet-152, ResNet-164, ResNet-1202 [93, 94].

**Fig. 7:**
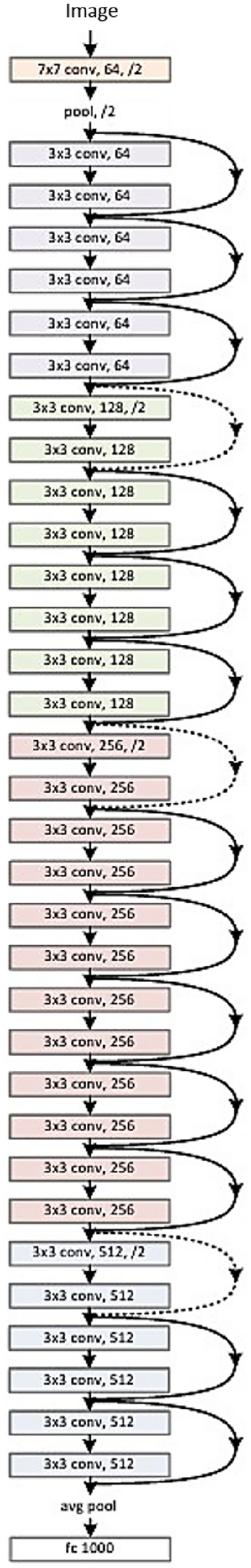
The really quite initial point we can see in the accompanying graphic is that there is a direct link that bypasses various model levels. The core of leftover blocks is a link known as a “skip connection.” This skip connection causes the outcome to differ. Input ’X is calculated by the layers weight in the absence of the skip connection, and then a bias factor is added [94]

ResNet based on two intuitions:

1. Error rate shouldn’t decline as we add more layers and go deeper.
2. To reconcile the expected with the real, continue to training the residuals.

These are the functions of a Residual Network.

**Fig. 8:**
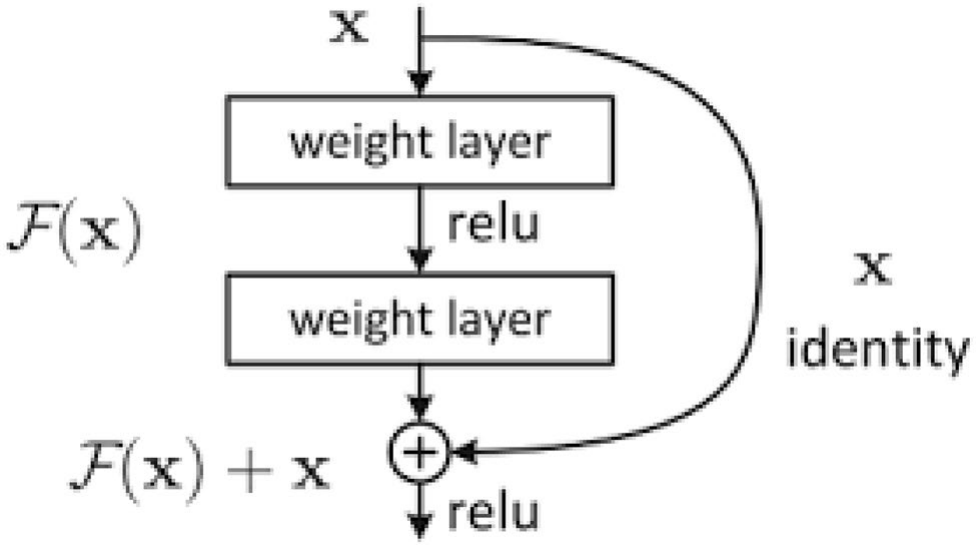
Residual Block [94]

12 $$\begin{aligned} y= & {} F(x, W_{i}) + x \end{aligned}$$

13 $$\begin{aligned} y= & {} F(x, W_{i}) + W_{sx} \end{aligned}$$

These two are the equations used where x & y are input and output vectors.

### Highway Networks

Highway networks are used to increase the depth of the neural network and it also does networks optimization. Highway networks uses gating functions approach to regulate information flow. Highway networks are inspired by Long-Short Term Memory recurrent neural networks (LTSM). Highway networks are mostly used in speech recognition tasks and sequence labeling [95].

**Fig. 9:**
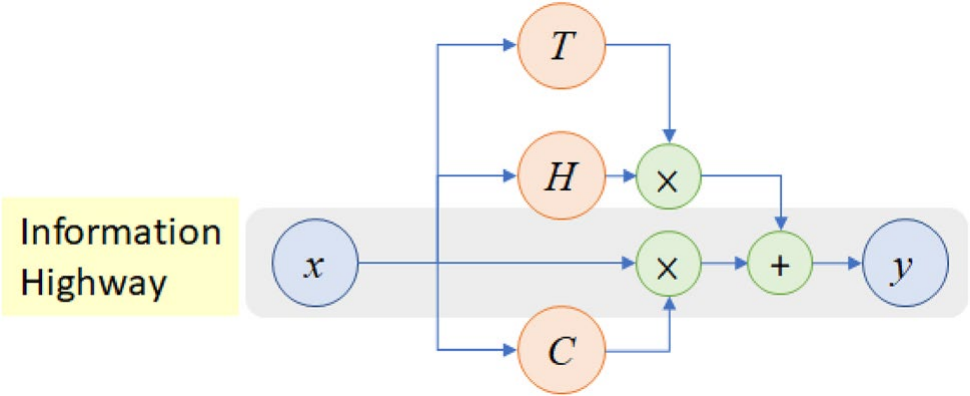
It enables direct communication between neurons in various levels. Data transmission is regulated by a gating technique. Data can go through various levels of neural networks thanks to gating processes [95]

In highway network, two non-linear transforms **T** and **C** are introduced:

14 $$\begin{aligned} y = H(x, W_{H}) \cdot T(x,W_{T}) + x \cdot C(x,W_{C}) \end{aligned}$$

where T is the Transform Gate and C is the Carry Gate. In particular, *C = 1 - T*,

15 $$\begin{aligned} y = H(x, W_{H}) \cdot T(x,W_{T}) + x \cdot (1 - T(x, W_{T})) \end{aligned}$$

We can have below conditions for particular T values:

16 $$\begin{aligned} {\left\{ \begin{array}{ll} x, &{} \text { if } T(x,W_{T}) = 0, \\ H(x, W_{H}), &{} \text { if } T(x, W_{T})=1 \end{array}\right. } \end{aligned}$$

When $\hbox {T}=0$, we pass the input as output directly which creates an information highway. That’s why it is called Highway Network. When $\hbox {T}=1$, we use the non-linear activated transformed input as output.

### DenseNet

DenseNet is used in object recognition. DenseNet has proven better than the ResNet because it outperforms ResNet in object Recognition. The architecture of DenseNet and ResNet are almost similar but there is a slight change that plays an important part in outperforming ResNet. DenseNet uses concatenation between layers while ResNet uses additive method. DenseNet requires GPU support because it uses concatenation [96].

**Fig. 10:**
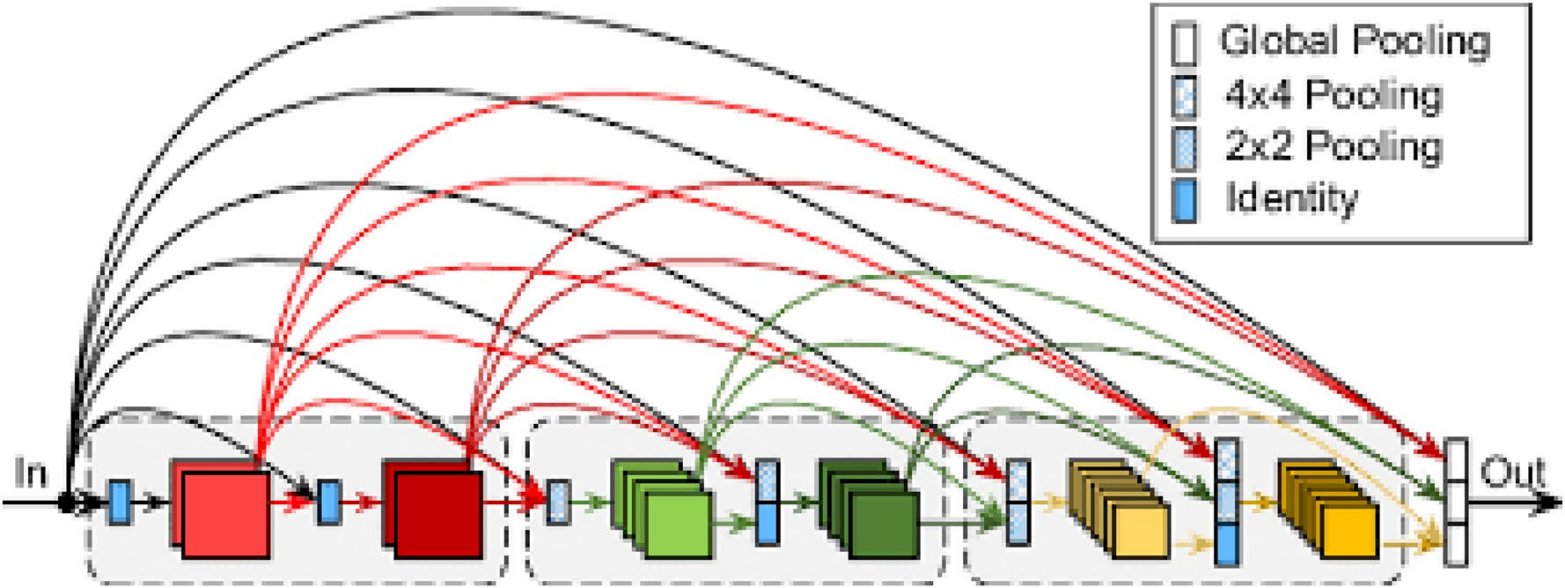
ResNet and DenseNet are relatively similar, however there are several key distinctions. While DenseNet combine the outcome of the prior level to the next level, it employs the addition technique(+) that combines the previous layer with the subsequent layer [96]

Its main advantage is that it doesn’t allow data to vanish from the input layer to the output layer.

### Wide ResNet

Wide ResNet is a modified version of ResNet. Wide ResNet is called wide Residual Network because there is increase in feature map size per each layer. WRN architecture is quite identical to the ResNet architecture but there is increase in the feature map size per layer it means that there is increase in the number of channels created in per convolutional layer [97].

**Fig. 11:**
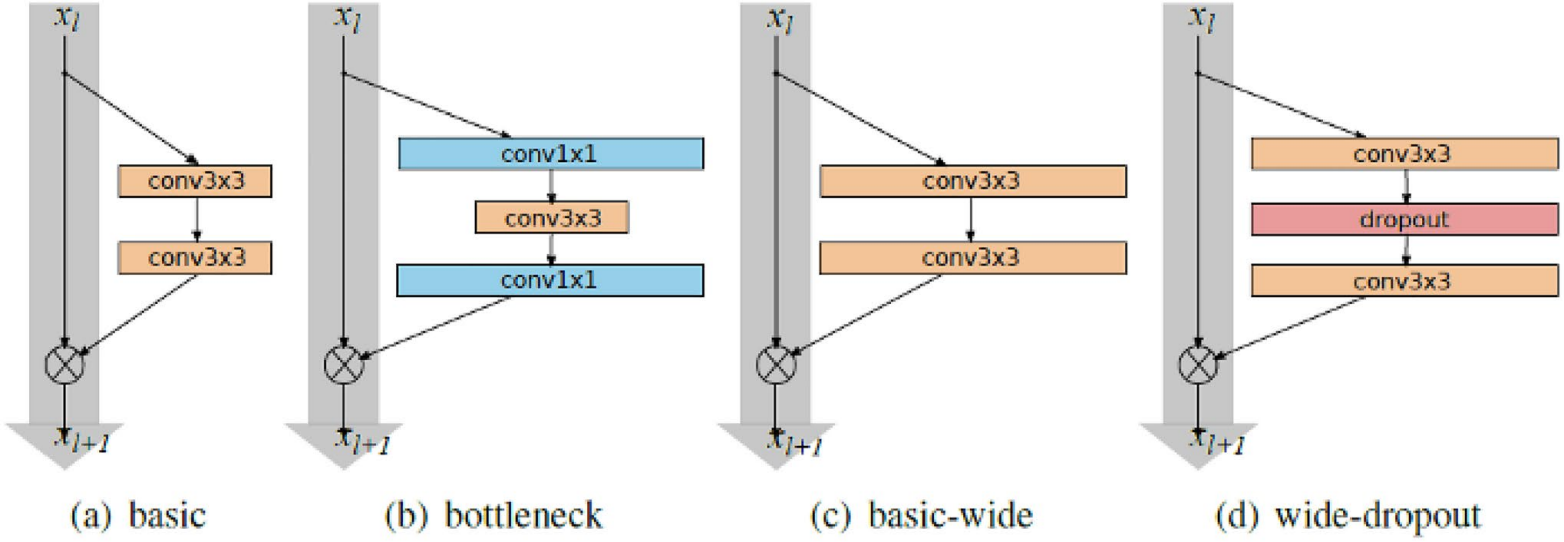
It has same accuracy as ResNet but number of layers is reduced and training time is shorter. WRN-22-8 outperform ResNet-1001 by 0.92% on CIFAR-10 and 3.46% on CIFAR-100. WRN-40-4 is 8 times faster than ResNet-1001 [97]

In WideResNet, order changed to BN-RELU-CONV. It add more convolutional layers per block and Increases filter size in convolutional layers. It has widening factor k and network includes 40 layers with $\hbox {k}=2$ times wider than the original would in WRN-40-2 and 4 times in WRN-40-4.

### Pyramidal Net

It has two approaches (1) Bottom Up Pathway is the feedforward calculation of the spine Convolutional Net. It is characterized that one pyramid level is for each stage. (2)Top down pathway and Lateral Connection—The higher goal highlights are up inspected spatially coarser, yet semantically more grounded, include maps from higher pyramid levels. All the more explicitly, the spatial goal is up examined by a factor of 2 utilizing the closest neighbor for straightforwardness [98].

**Fig. 12:**
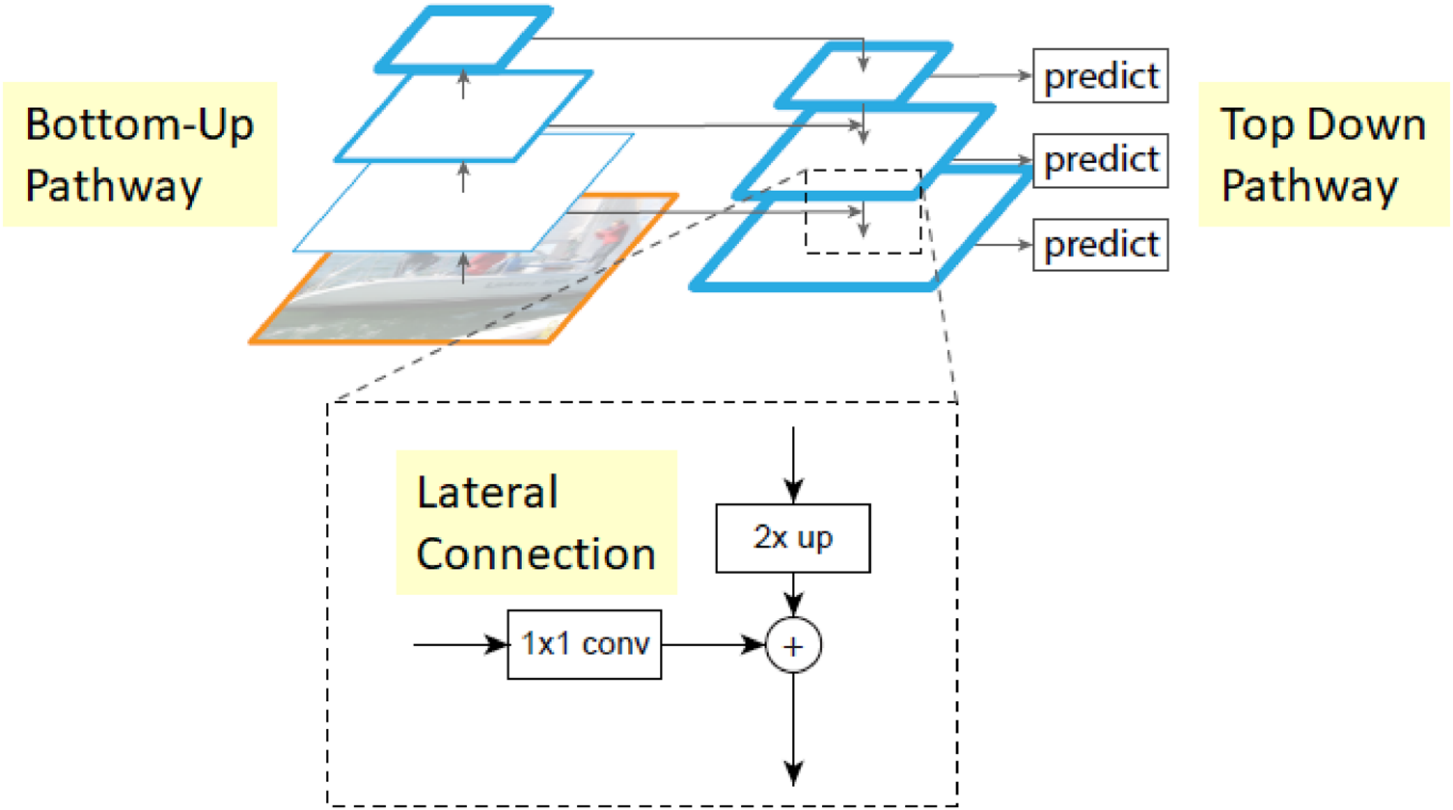
Pyramidal Net: [98] Two popular approaches top down and bottom-up are used in pyramidal network of CNN

Specifically, the element maps from base up pathway goes through 1 × 1 convolutions to lessen the channel measurements and the element maps from the base up pathway and the top-down pathway are converged by component astute expansion. Finally, a 3 × 3 convolution is annexed on each combined guide to create the last component map, which is to diminish the associating impact of up inspecting. This last arrangement of highlight maps is called P2, P3, P4, P5, relating to C2, C3, C4, C5 that are separately of similar spatial sizes.

### Inception

Inception is a convolutional neural network (CNN) that is used for object detection and image analysis. Contrarily, the Inception structure was intricate (heavily engineered). It employed several strategies to increase speed and accuracy of execution. Numerous variations of the network were produced as a result of its ongoing development. Deep neural networks are costly to compute. The researchers reduce the cost by including an additional 1 × 1 convolution w preceding 3 × 3 and 5 × 5 convolution layers. This reduces the amount of input vectors. Contrary to what would seem logical, 1 × 1 convolutions are significantly more affordable than 5 × 5 convolution layers, and the fewer input streams also aid. But keep in mind that the 1 × 1 convolution is added after the max pooling layer, not before [99].

- *Inception V1* It performs convolution on the input with 3 different size i.e. (1 × 1, 3 × 3, 5 × 5).
- It also performed max pooling.
- The output will be concatenated and send to the next inception module.
- *Inception V2*
- It performs convolutional on the input with 2 different size i.e. (1 × 1, 3 × 3) as the major change is that 5 × 5 replaced to two 3 × 3 convolution.
- This decrease computational time and thus increase computational speed.
- 3 × 3 convolutional is 2.78 lesser than 5 × 5.
- It also converts $\hbox {n}\times \hbox {n}$ factorization to 1xn and nx1.
- It is 33% cheaper than the nxn factorization.
- *Inception V3* It is similar to V2 with the following changes:
- RMSprop optimizer.
- Batch normalization in the fully connected layer.
- 7 × 7 factorized conv.
- Label Smoothing Regularization.

**Fig. 13:**
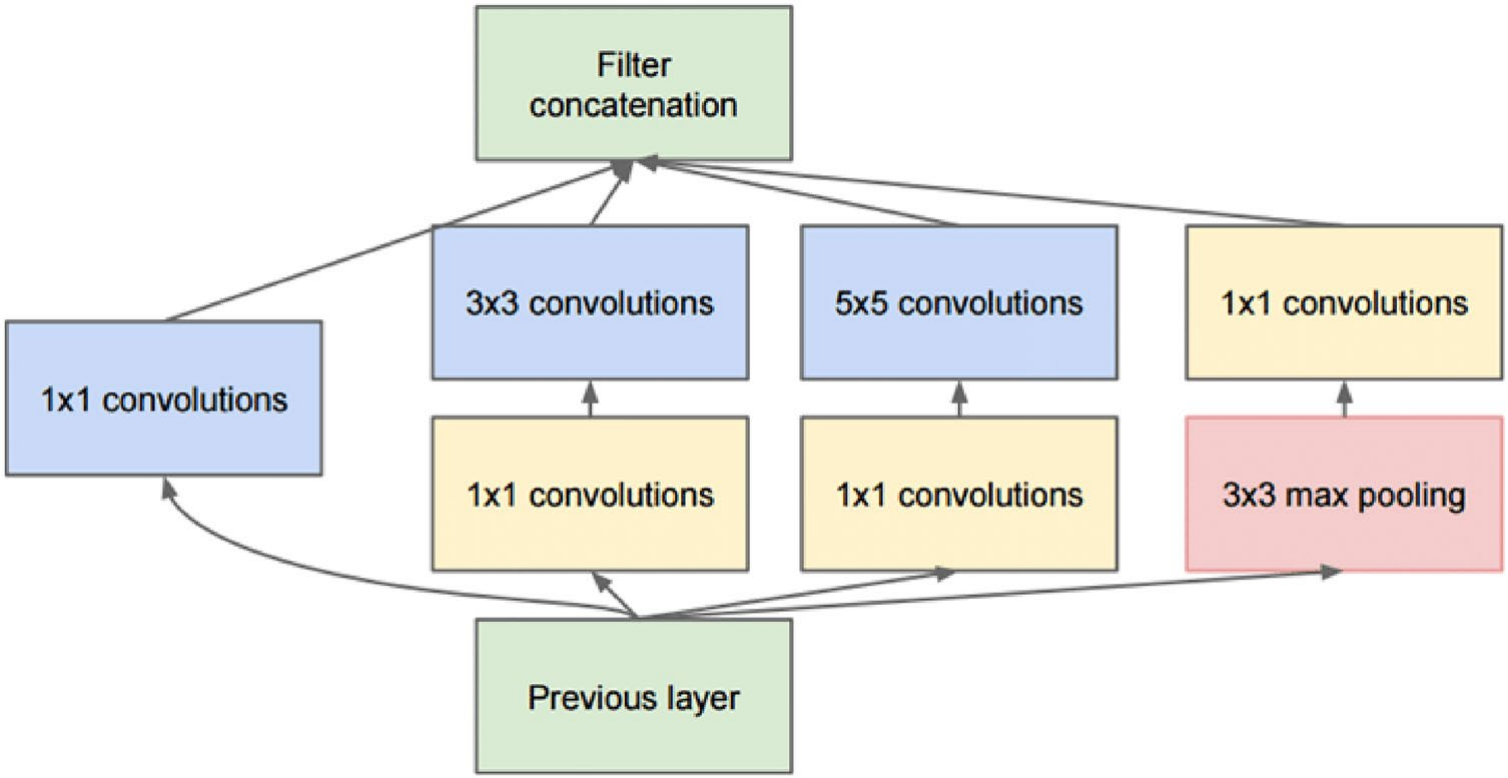
Inception V1 [99]

**Fig. 14:**
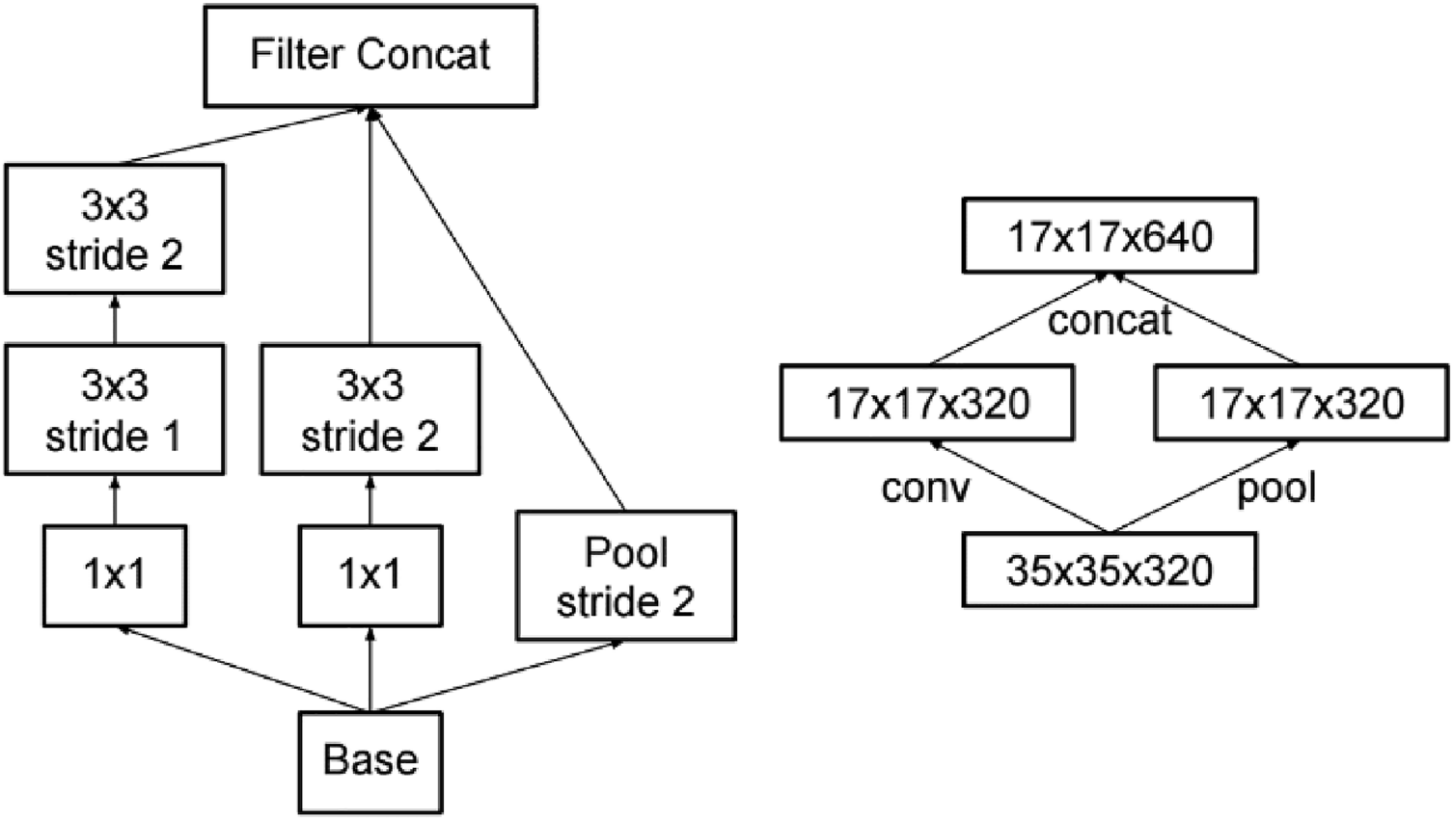
Inception V2 [100]

**Fig. 15:**
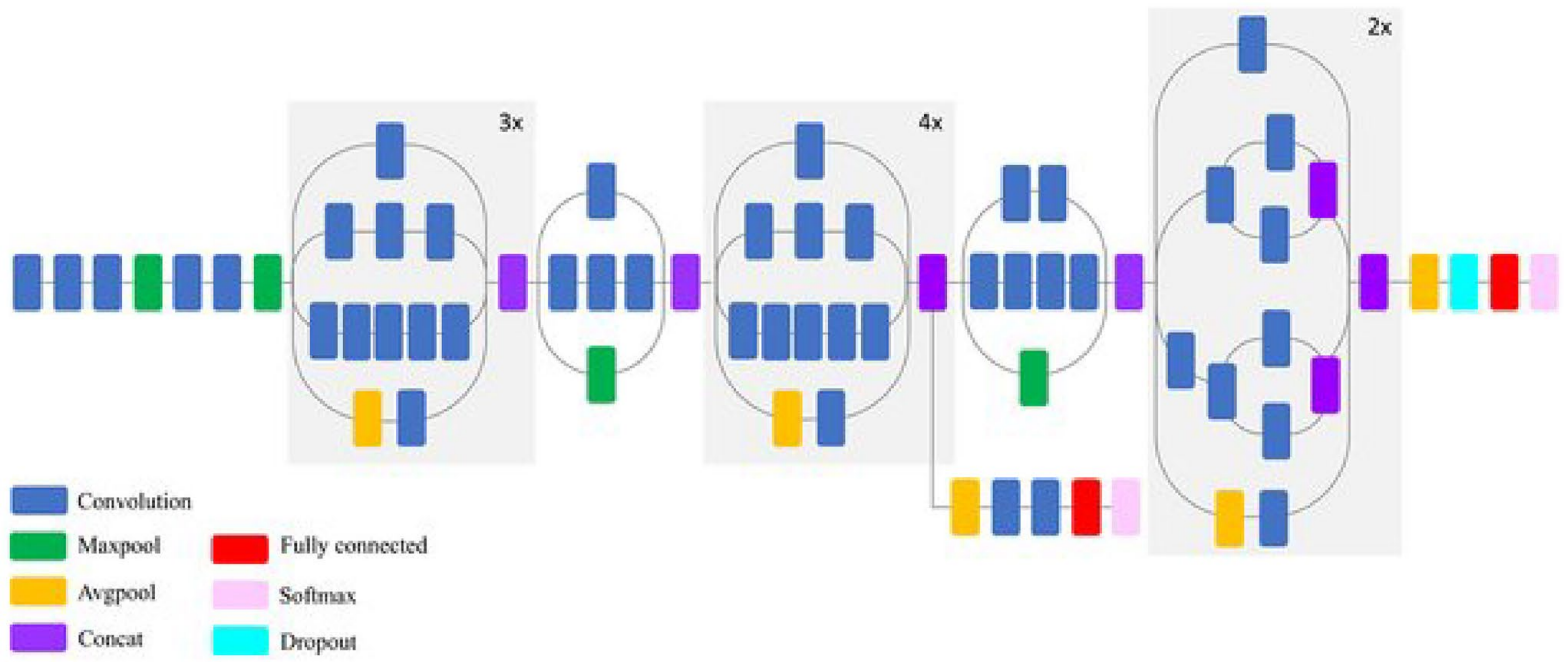
Inception V3 [101]

### Xception

Xception is a convolutional neural network that was introduced by Francois Chollet. Xecption net is the further extension of Inception neural network. Its architecture is similar to the architecture of the inception model but it is further extended by using depth wise separable convolutions. In Xception network the model parameters are used efficiently and that’s why it outperforms inception model. The channel-wise $\hbox {n}times\hbox {n}$ spatial convolution is known as depth-wise convolution. If there are five channels in the aforementioned diagram, we will get five $\hbox {n}times\hbox {n}$ spatial convolution. The 1*x*1 convolution used to adjust the scale via pointwise convolution [102].

**Fig. 16:**
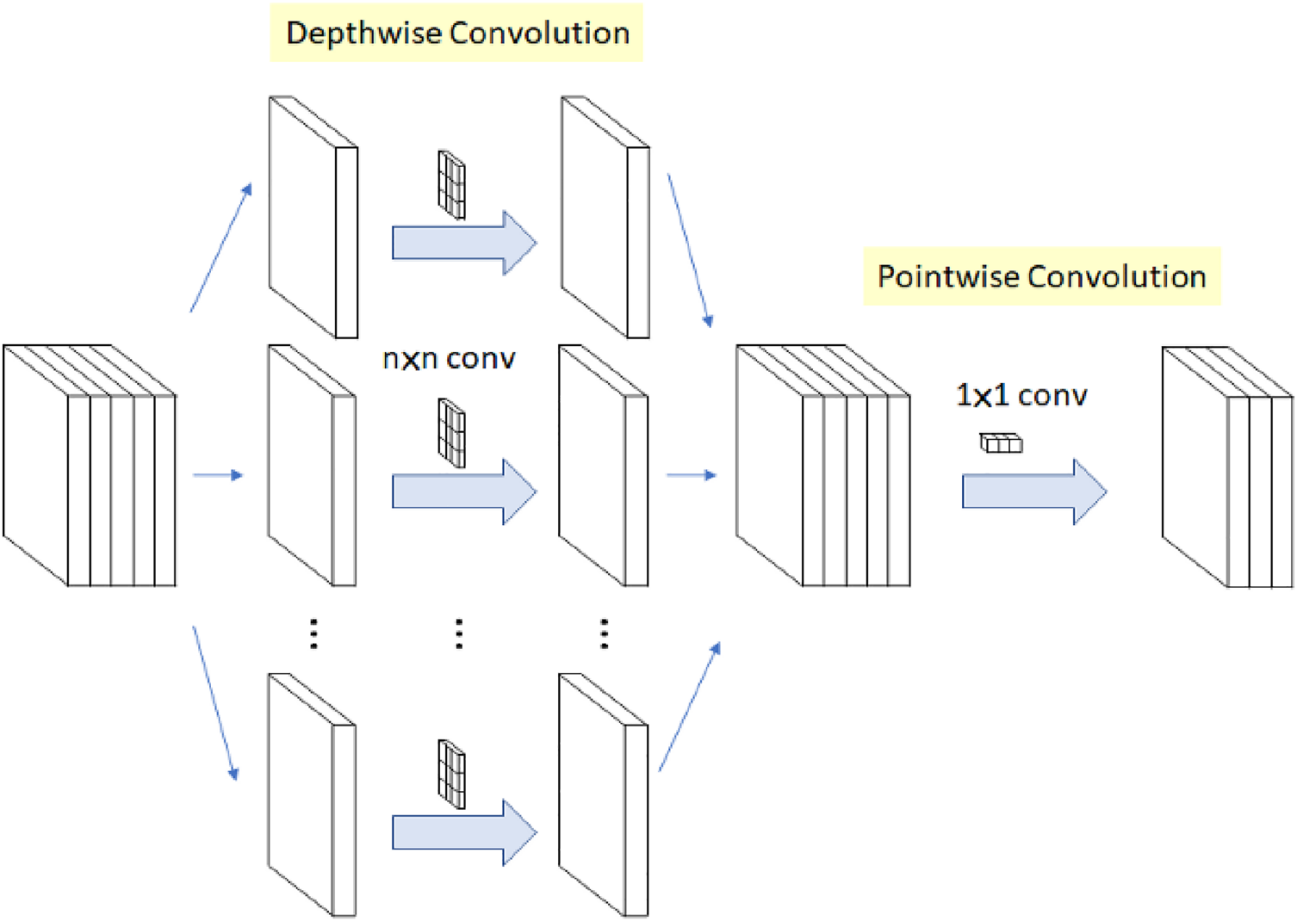
Xception is a convolutional neural network architecture that relies solely on depth wise separable convolution layers [102]

Although Xception uses the same amount of parameter estimation as Inception V3, it does so more effectively. This framework, known as Xception, considerably surpasses Inception V3 on a bigger image classification sample that consists of 350 million images and 17,000 classes, outperforming Inception V3 on the ImageNet dataset (about which Inception V3 was built). The model is substantially lightweight and has fewer components, which is its major benefit.

### ResNeXt

ResNeXt is designed for efficient image classification. The architecture of ResNeXt consist of stacks of topology blocks. Width and Filter size is also shared which are also known as hyper-parameters. It uses cardinality which refers to the size of the set of the transformations. It is an important part in the addition to dimensions [103].

**Fig. 17:**
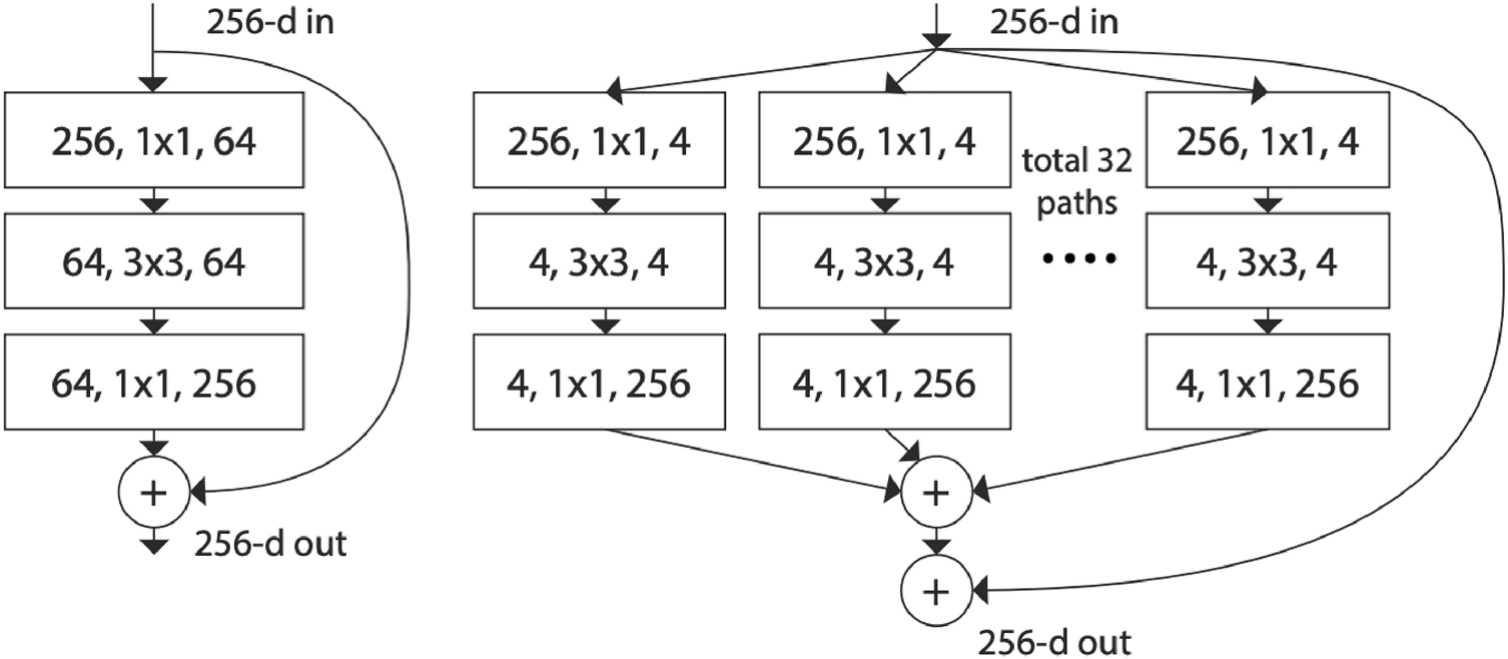
A straightforward, extremely modular design network framework called ResNeXt is used to classify images. Recurring a building block that combines a group of transformations with the identical structure forms the foundation of our network [103]

Comparable to an Inception Module, it collects a collection of transformations using a “split-transform-merge” technique. Our straightforward approach yields a homogenous, multi-branch framework with a minimal amount of hyper-parameters. In contrast to the measurements of depth and breadth, this technique also reveals a new aspect, which we refer to as “cardinality” (the magnitude of the collection of transformations).

### Squeeze Net

Squeeze Net is a deep convolutional neural network that was introduced in 2016. Squeeze Net was introduced over Caffe framework but later on it has been formed on different networks. It is a CNN model that uses different design strategies to minimize the parameters. Smaller network so less communication across serves within training phase. Due to the lesser so its require minimum bandwidth to export a new model from the cloud to an autonomous car [104]. More flexible to deploy on FPGAs and other types of hardware with limited memory. It uses the following strategy for uniqueness:

- Replace filters (replace the 3 × 3 filters with 1 × 1)
- Decreases the number of input (decreases the number of input channels to 3 × 3 filters if layer has 3 × 3 filters so total parameters in layer is (number of input channels)  ×  (number of filters)  ×  (3 × 3)
- Down Sampling (Down sample late in the networks do convolutional layers have large activation function maps.

When we use Fire modules so we set 1 × 1 to be less than ($\hbox {e1}\times 1+\hbox {e}3 \times 3$), so the squeeze layer helps to limit the number of input channels to the 3 × 3 filters.

**Fig. 18:**
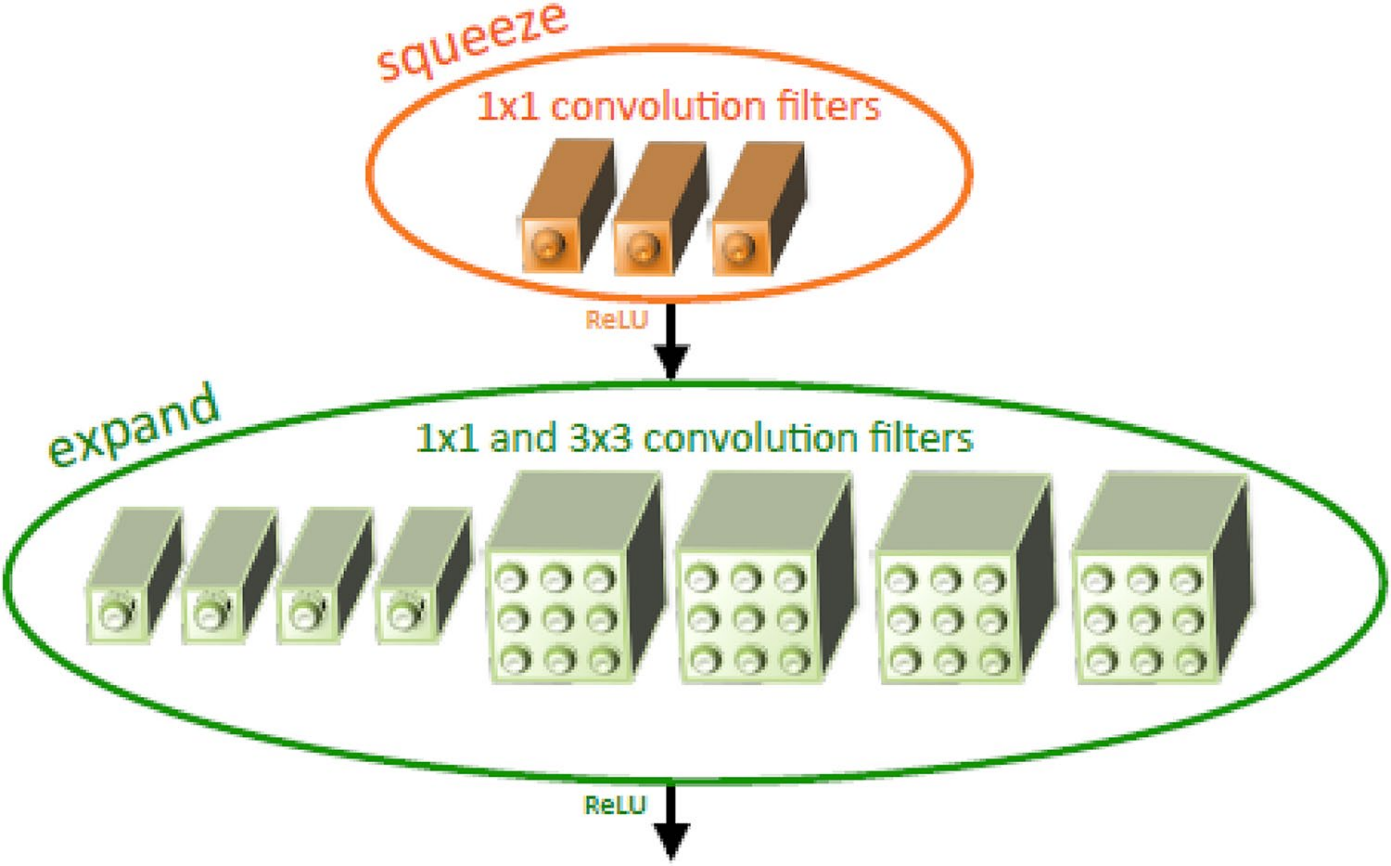
Fire module is comprised of squeeze convolutional layer feeding into an expand layer and that has a mix of 1 × 1 and 3 × 3 convolution filters [104]

**Fig. 19:**
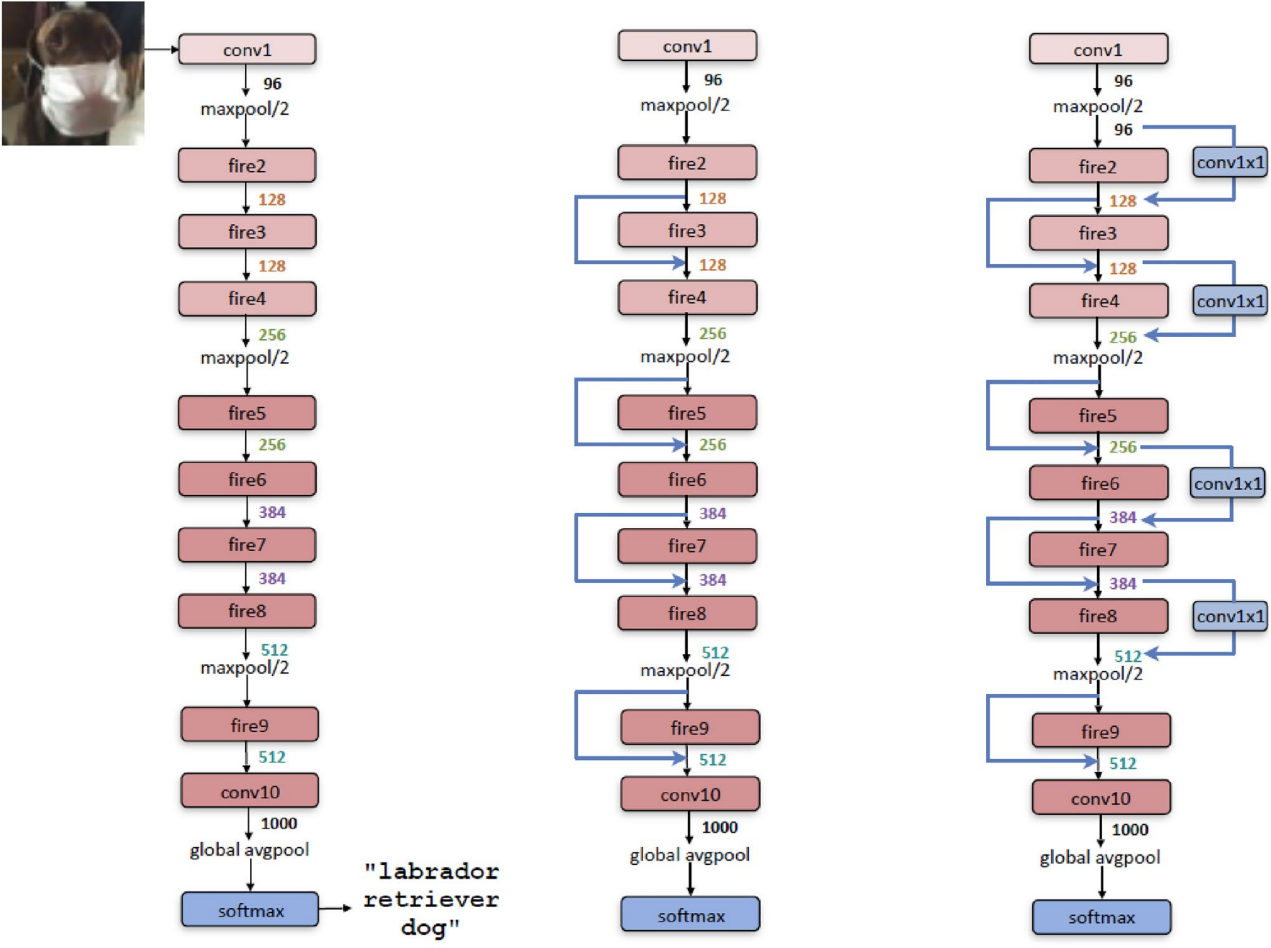
Squeeze Net is smaller network architecture that was designed as a more compact replacement for AlexNet and it perform 3 times faster and it is used in mage classification [104]

In left side begins with s single layer followed by 8 Fire module (fire 2–9) ending with final layer (Convolutional 10). Both complex and simple bypass connection yield an accuracy improvement over the vanilla Squeeze Net architecture. With using the fire module, we can reduce the size of model while maintaining the prediction the accuracy.

### U-Net

U-Net is a convolutional neural network that was introduced for image segmentation in the biomedical field. It was introduced by Olag Ronneberger. Its architecture consist upon encoder and decoder. The encoder in its architecture deals with covenant layers that are followed by the pooling operation. The Decoder part in U-Net architecture uses transport convolutions for localization [105].

**Fig. 20:**
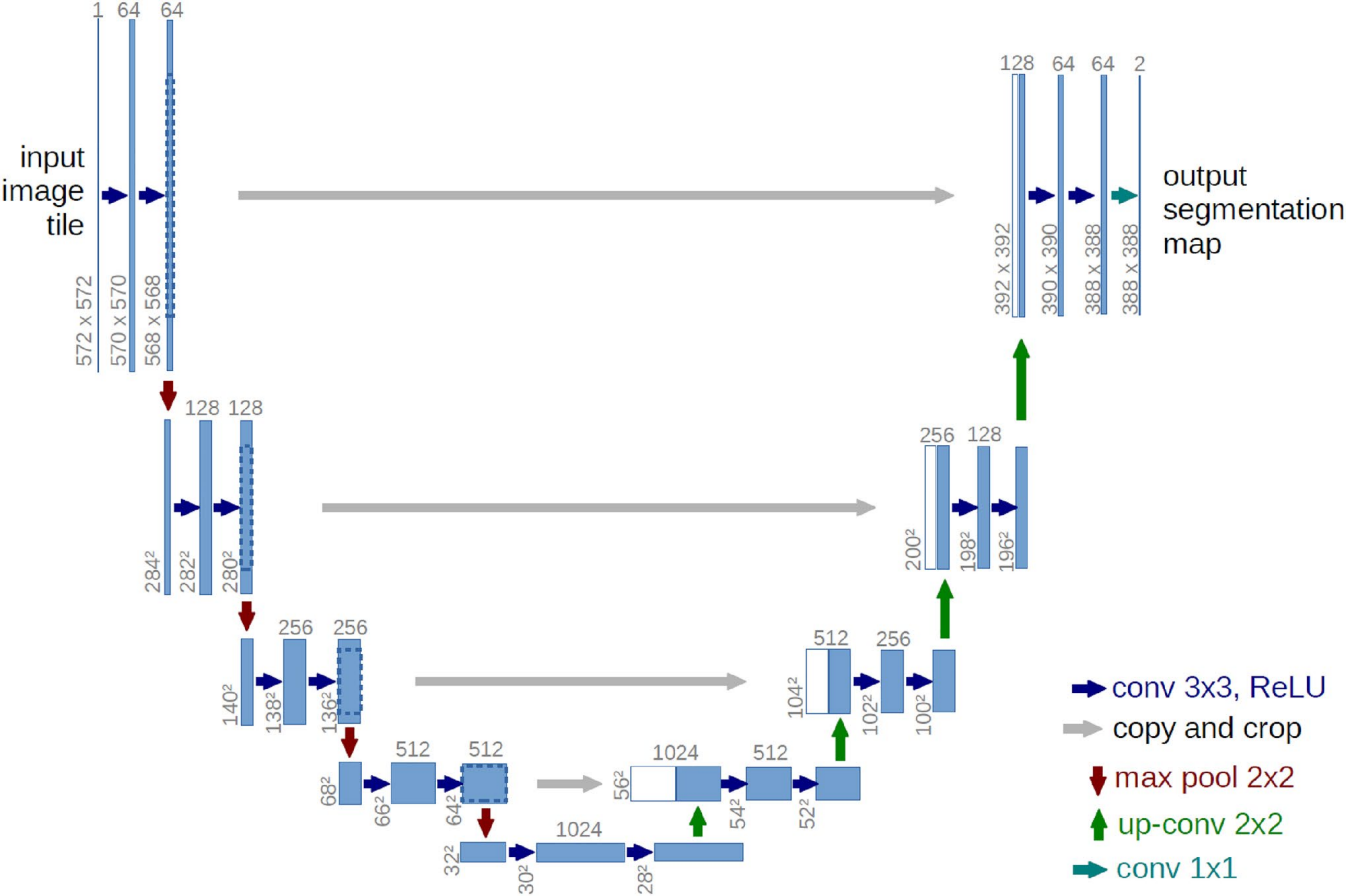
UNet is a CNN network architecture that expanded with few changes in CNN architecture. This model was invented to deal with biomedical field I this image can take input and this model target was not only classifying the image it can also identify the infection as well as infection location or area [105]

According to the image this model architecture looks like u and its justify the name. this model architecture consists of three section

- Contraction
- Bottleneck
- Expansion section

Contraction section is made of many blocks and each block takes input applies  ×  convolutional layers followed by a 2 × 2 max pooling. We can say that main part or heart of this architecture lies in expansion section this section was similar to the contraction like make block and each block several layers etc. But each block of feature mas used by convolutional layer get half to maintain the symmetry. Loss Calculation in UNet so it is the energy function that is computed by a pixel wise soft max over the final feature map combined with the cross entropy loss function. Image segmentation is important so UNet contributed significantly in such manner to help and solve the problem.

### V-Net

V-Net (Volumetric-Convolutional Network) is a Convolutional network that was introduced medical image segmentation of 3D images. Before Vnet most of the bio medical image segmentation was done using 2D images. Vnet CNN model is trained on 3D MRI scans to perform image segmentation [106].

**Fig. 21:**
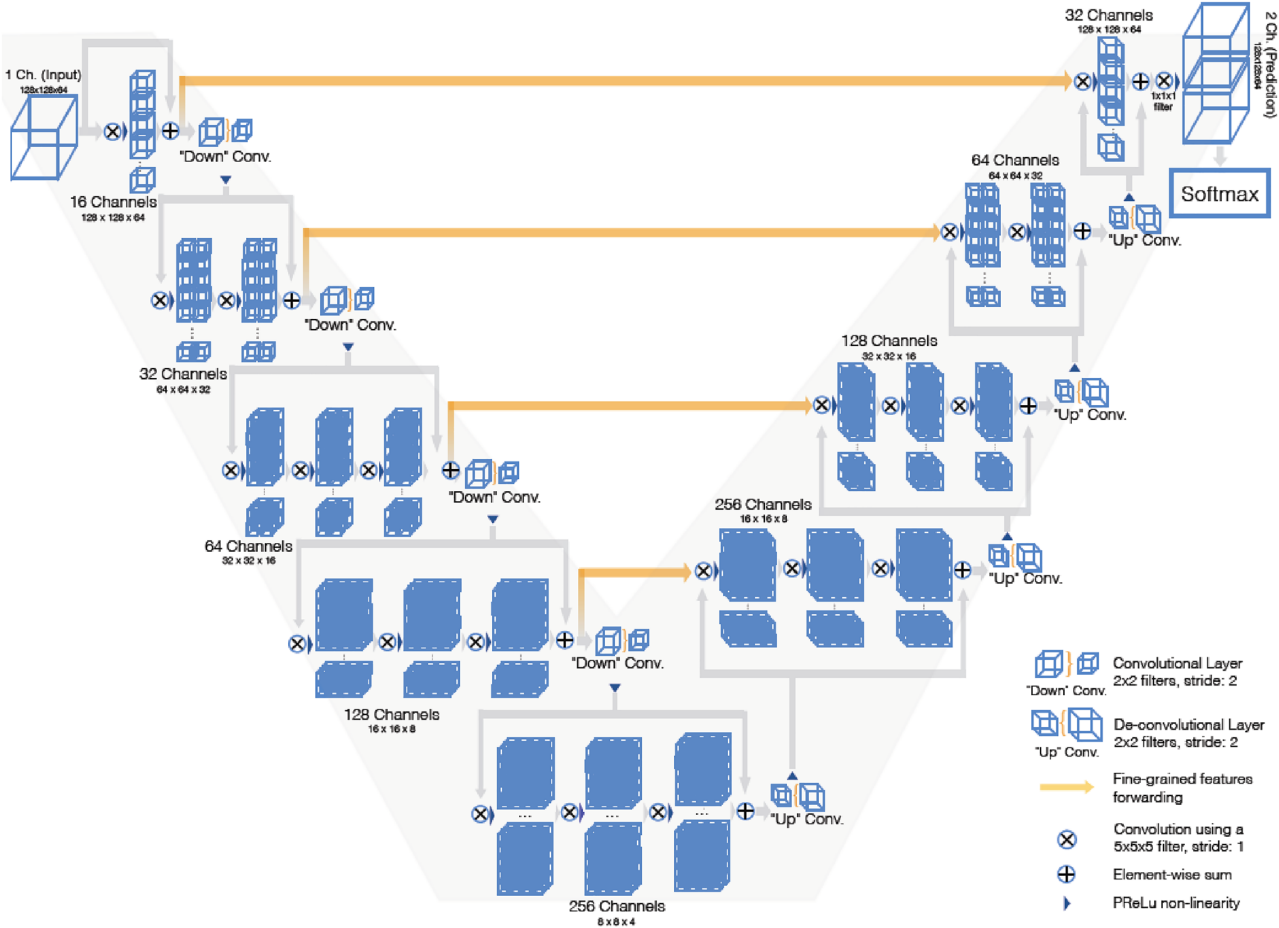
It is convolutional neural network architecture that can handle biomedical field and help out to perform the segmentation so it called volumetric network because detailed medical terms are not easy so experts required for detail which is high cost and automatic segmentation can help to reduce the cost in other words we can say that to achieve the high accuracy [106]

Left part of the architecture consists of the compression path and right part decompress the signal until its original size reached.

- *Left Side* This side divided into different stages and every stage comprise one to three convolutional layers. Each stage residual function is learnt all layers connected and added to the last output convolutional layer of that stage in order to enable learning a residual function. Convolutional performed in each stage use volumetric kernels having size 5 × 5 × 5 voxels. (Voxels represent the value on s regular grid in 3d space). Output of the pooling layers needed for back propagation. Down sampling also perform because it helps to increase the respective field. In this architecture PReLU is used as activation function.
- *Right Side* This side of network extract the features and expand the spatial of the lower resolution feature maps in degree to collect and generate the important information to output two channel V segmentation.

At each stage reverse of the convolutional layer perform deconvolution to increase the size of the input followed by one to three convolutional layers. Residual function learnt is similar to the left part. Last convolutional layer computed two feature maps having 1 × 1 × 1 kernel size and produce the outputs of the same size as input volume. These features maps are the probabilistic segmentation of foreground and background regions by applying soft max function voxel wise. Feature extracted from early stages of left part so that will have forwarded to the right part through this connection and this will help to provide location information to the right side so that’s cause improve or better the quality of the final contour prediction.

### SegNet

SegNet is a convolutional network that was introduced by the researchers of the University of Cambridge. SegNet architecture consist of encoder and decoder. SegNet uses encoder and decoder based architecture for multiclass pixel-wise segmentation. SegNet performs very well in indoor scene understanding [107].

**Fig. 22:**
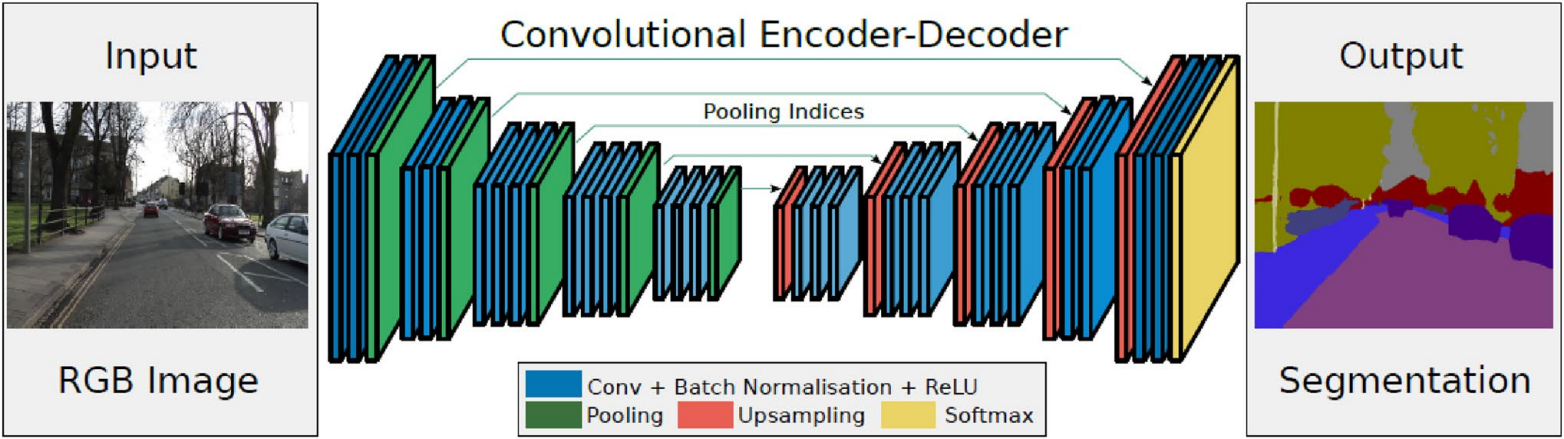
It is deep encoder and decoder architecture for multi class and in this we can segmentation pixel wise [107]

In encoder convolutional layers with batch normalization and a ReLU non-linearity followed by non-overlapping max pooling and subsampling in other words we can say that down sampling. In this network there are 13 convolutional layers from VGG-16. During the 2 × 2 max pooling corresponding max pooling locations can be stored. In the decoder convolutional as well as up sampling are performed in the end softmax classifier is present for each pixel. In above image show during the up sampling max poling indices the corresponding encoder layer then it is called up sampling. So ate end soft max classifier is present which can predict class for each pixel.

### Unet++

Unet++ is the modified version of Unet CNN. It provides more accuracy with a series of nested, dense and skip pathways. The changes that are made in Unet to modify it into a Unet++ are redesigned skip pathways, dense skip connections, deep supervision [108].

**Fig. 23:**
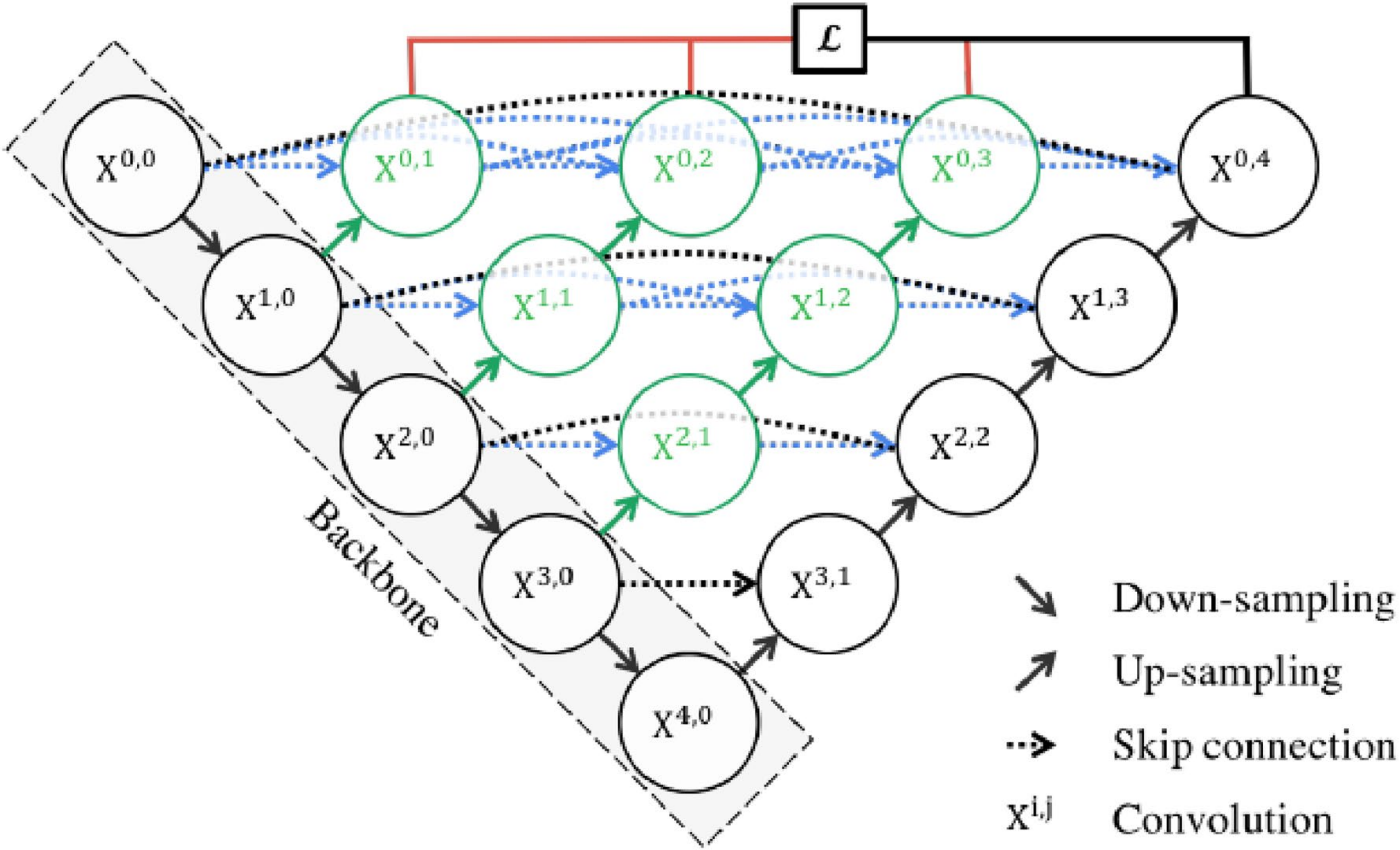
Unet is a CNN architecture that expanded with few changes in the CNN. This architecture was invented to segment the bio medical images like not only classify the image it can also identify the infection as well as infection area [108]

Unet++ is uses the dense block and its differ from Unet with three ways:

- CL (Convolutional layers) on skip pathways.
- Dense skip connections on skip pathways.
- Deep supervision which enables model pruning.

In first point or step which bridges the semantic gap between encoder and decoder and second point will tell improves the gradient flow third and last point tells achieve comparable performance to using only one loss layer. This network starts with encoder and decoder sub-network and backbone followed by a decoder sub network. There are re-designed skip pathways green and blue that connect the two sub networks and use of deep supervision (red). Above figure shows that how the feature map travel through top skip pathway of unet++. Each layer in convolutional is preceded by a concatenation layer that fuses the output from previous layer of the same dense block with corresponding up sample output.

### FCNN

Fully convolutional neural network model is a deep learning model based on simple and classic convolutional neural network. It can take input in any arbitrary size. In Fully convolutional neural network there are no fully connected convolutional layers. FCNN has learning filters everywhere. FCNN also has filters in decision making layers.

### Fast-RCNN

Fast-RCNN was introduced in 2015 by [109]. Fast-RCNN is used for faster object detection. Its architecture is pretty much similar to the RCNN but in Fast-RCNN we direct give input image to convolutional neural network to generate a convolutional feature map. The reason that the fast-RCNN is faster than the RCNN is that there is no need to give input a lot of region proposals to CNN every time.

### Mask R-CNN

Mask R-CNN is used for instance segmentation. Mask R-CNN is formed on the base of Faster R-CNN. Mask R-CNN uses anchor boxes to detect multiple objects and overlapping objects in an image. This method increases its speed [110].

### DeepLab

DeepLab was introduced in 2016 by Google. It is one of the best semantic segmentation models present. DeepLab has been modified and improved over the years and now there are many promising versions of DeepLab present [111]. The following are the versions of DeepLab:

1. *DeepLab-v2* DeepLabv2 was built on DeepLab it contained an atrous spatial pooling scheme. It consist of parallel dilated convolutions with different rates applied in the input feature map.
2. *DeepLabv3* DeepLabv3 was an improvement made over DeepLabv2, in this version the problem of segmenting objects at multiple scale was solved by designing and employing atrous in cascade to capture multiscale context by adopting multiple atrous rates.
3. *DeepLabv3+* Deeplabv3+ is an improvement over DeepLabv3+. In this version semantic labels are assigned to every pixel in the given input image.

### RetinaNet

RetinaNet is used for one stage object detection models. RetinaNet provides good results in dense and small objects detection. RetinaNet is mostly used with aerial and satellite object imagery. RetinaNet is formed by making improvements in existing object detecting models which are Feature Pyramid networks and Focal Loss [112].

### YOLO

YOLO (You only look once) is a network that is used for object detection in deep learning. YOLO is faster than its competition and is mostly used in real time object detection. YOLO does object detection by classifying different objects in the given frame [113]. Yolo has been modified year by year and has been converted into different versions:

1. *YOLOv4* YOLOv4 is an object detection network that can be operated on single GPU with a smaller mini batch size. YOLOv4 increases the speed of object detection for the systems with a single GPU.
2. *PP-YOLO* PP-YOLO (Paddle Paddle-YOLO) is an improvement over YOLOv4 it has improved inference speed and mAP score.

Channel (Input) exploitation based CNNs The lack of variance and the absence of distinct information within the input might have an effect on CNN’s performance as an individual that’s why the concept of channel boosting was proposed to boost the representation of the networks.

Attention is the method that is used to copy cognitive attentions. This effect will magnify the important parts of input data and will fade out the all-other data.

## Deep Learning for Medical Image Analysis and CAD

CNN’s beginnings may be traced back to the nonrecognition idea put out by [114] in the beginning 1980 s. In early 1990, [115] training a CNN using backpropagation to categories sequences of character recognition. In the initial nineties, CNN was employed in several domains including image identification, character segmentation and facial detection. In 1993, [116] trained a CNN to recognize pulmonary nodules in chest radiographs, which was the first time CNN had been used to analyze healthcare images [117]. Chan et al. [118–121] used CNN for lung nodules and microcalcification recognition [122, 123] on radiography within the identical year. In 1994, [124] used a comparable shift-invariant neural network to find regions of microcalcifications. Despite the fact that these initial CNNs seemed not really sophisticated, they were able to recognize patterns in healthcare images. Profound CNN was made possible by a number of crucial neural network learning methods that have been evolved over time, such as layer-wise unsupervised representation learning accompanied by closely monitored fine [125–127], the use of rectified linear unit (ReLU) [128, 129] as an activation function in place of sigmoid operations, pooling to enhance functionality normalization and minimize granularity [130], dropout to control under- and over-fitting problem [131] and batch normalization [132]. Such methods enable the training of neural networks with increasing layers and parameters. In 2012, [133] presented a CNN with five convolution layer and three fully connected layers (named “AlexNet”) that contained more than 60 million weights. This CNN outperformed previous models in the ImageNet Large Scale Visual Recognition Challenge (ILSVRC) [134], which categorized more than 1000 categories of common items on images. AlexNet exhibited the hierarchical structure’s several layers’ capacity for pattern classification. Since AlexNet, DCNNs have been built with higher density.

The 2015 ILSVRC was won by [94], who presented residual training and demonstrated that a residual network (ResNet) having 110 to 152 hidden layers might beat a number of previous DCNNs. According to [135], a DCNN’s learning ability grew with complexity, but it was only used if the training set were adequately substantial.

Unexpected hopes for deep learning in healthcare have been elevated by the achievement of AI and learning techniques in driving-less cars, social networks, board and GO games. Numerous medical image analysis tasks for CAD have used deep learning [136–138]. The categorization of illness and healthy trends, the classification of cancerous and mild tumors and the forecasting of high- and low-risk trends of acquiring tumors in the long term are among the major popular applications of deep learning in CAD. The division and categorization of various parts of the body and tumor classifications, as well as the categorization of alterations in tumor volume or appearance for the evaluation of medication reaction or the forecast of outcome or recurrence, were further uses. Numerous research for lung illnesses and prostate tumors were carried out utilizing the public data sets since there exist sizable public data sets for Computed Tomography (CT Scan), thoracic CT and mammography. The diagnosis of eye disorders using retina images or optically Computed Tomography (CT Scan), as well as the categorization of cell types using histopathological images, have all been accomplished using deep learning-based imaging techniques [139, 140]. The majority of the experiments presented extremely encouraging findings, adding to the excitement around deep-learning-based CAD. Even though this latest range of CAD technologies is referred described as AI, they are nowhere near being ‘intelligent’, instead behaving like an extremely complicated numerical method that memorizes data in its billions of weights.

One of the earliest applications of deep learning to medical image processing is image or assessment categorization. Generally, one or more image graphs (an assessment) are used as the source and just one medical parameter is used as the outcome in exam categorization (e.g.,disease present or not). Every screening test serves as a trial in this situation, and dataset volumes are often lower than in machine vision (e.g., hundreds or even thousands vs. millions of observations). Hence, it is not unexpected that ensemble learning is attractive for these purposes.

Attempt to circumvent the (considered) need for massive amounts of data for deep CNN training, ensemble learning basically involves using pre-trained networks (usually on real imagery). Using a pre-trained CNN model as a feature representation and fine-tuning a pre-trained CNN model on health data were other transfer learning methodologies that were discovered. The suggested technique also has the advantage of not having any deep CNN training, making it simple to integrate the derived features into current image processing workflows. These methods are well-liked and often used. Many writers, though, actually provide a detailed analysis of which approach produces the greatest results. Kim et al. [141] and [142], two studies that attempt, present inconsistent findings. In the study by [142], fine-tuning achieved 57.6% performance in inter class evaluation of knee osteoarthritis against 53.4% performance for extracting features. However, [141] demonstrated that the efficiency of cytopathology image categorization was improved by utilizing CNN as a feature representation (70.5% vs 69.1%).Several current articles, both issued in reputable journals, that optimized a pre-trained CNN variation of Google’s Inception v3 framework on healthcare records and attained (nearly) human expert efficiency are cited if any insight can be offered as to which approach could be the most effective [143, 144]. Such findings have not yet been obtained utilizing only pre-trained CNN models as feature representation, as far as the researchers are concerned.

A time-frame resembling machine vision is evident for the neural architectures that are frequently utilized in test categorization. Traditionally, the medical imaging industry concentrated on network designs like SAEs and RBMs as well as unsupervised pre-training CNN models. The very earliest articles utilizing these methods for test categorization concentrated on neuroimaging and were published in 2013. Based on brain MRI, [145–148] used DBNs and SAEs to categorize individuals as suffering Alzheimer ’s syndrome. There has been a noticeable trend favoring CNNs presently. Among the 397 publications on test categorization that were released in 2018, 2019, 2020, 2021 and 2022, 313 use CNNs, 51 use AEs and 33 use RBMs. These techniques have a wide range of applications, including pulmonary CT Scans, retinal scanning, brain MRI, and computational pathology.

Instead of utilizing pre-trained CNN models, researchers of relatively latest researches using CNNs frequently develop their customized network designs from beginning. In tests evaluating fine-tuning of pre-trained CNN networks versus training from beginning, [149] shown that fine-tuning scored significantly given a limited collected data of roughly 1000 images of skin disease. But the size of these studies makes it impossible to extrapolate any broad generalizations.

Three studies had to utilize a layout that took advantage of the particular characteristics of healthcare records: two was using 3D convolutions rather than 2D to identify Alzheimer’s patients [150–152] implemented a CNN-like structure to a brain network diagram from MRI Diffusion Tensor Imaging (DTI). In effort to accomplish this, they created three additional layers, known as node-to-graph, edge-to-edge and edge-to-node layers, which served as the framework for their structure. They demonstrated that their structure surpassed current approaches in evaluating intellectual and physical scores by using their system to anticipate brain growth.

Mahapatra et al.  [153] present deep learning algorithms’ dependability and integrity are highly coveted qualities in the healthcare profession. In this study, they present an interpret-ability-guided inductive bias method that requires learned features to produce saliency maps that are more distinguishable and regionally stable for various classifiers of classification model, resulting in enhanced simulation results. By including a category cost and a regularization cost term for spatial coherence, they are able to accomplish our goals. The suggested methodology surpasses existing approaches, according to practical findings for healthcare image categorization and differentiation challenges, and produces feature map that are more in line with the opinions of healthcare staff. They also demonstrate the usage of unmarked image data to further improve efficiency. In conclusion, the suggested method produces better acquisition rates, model resilience, and model understand-ability. It is also flexible, adaptable to current infrastructure designs utilized for medical imaging purposes [153].

Lee and Chung [154] proposed a Deep Neural Networks (DNNs) classify medical images using transfer learning from image features to enable computer-assisted medical assessment. Contrarian threats are anticipated to be confined even though learning datasets (healthcare illustrations), which are frequently needed for malicious examples, are typically unreachable in contexts of privacy and safety conservation and protection, despite the fact that the adversarial weakness of CNN architectures impedes real world applications due to the elevated shareholdings of prognosis. However, in this paper, they proved that, despite the absence of such clinical images, antagonistic assaults are still feasible using visual features for healthcare neural network models with domain adaptation. In specifically, they established that Universal Adversarial Perturbations (UAPs) may be produced from image features. Both non-targeted and targeted assaults can benefit from UAPs derived from real-world images. UAPs generated from real images performed noticeably better than randomized counterparts. Transfer learning usage creates a privacy gap, which reduces the accuracy and trustworthiness of machine illness identification. Although UAP effectiveness from real images was lowered by model development from random selection, UAP susceptibility was still present.

Liu et al. [155] highlighting two issues that must be addressed for Semi-Supervised Learning (SSL) in medical image analysis (MIA) to be effective. Both inter- and cross-label issues should be successfully handled by it. Anti-curriculum pseudo-labeling is a novel SSL technique that they suggest in this research. It presents brand-new methods for choosing insightful unlabeled samples. In this study, they recommend an innovative SSL method called Anti-Curriculum Pseudo-Labeling (ACPL), that also incorporates innovative strategies to can choose insightful unmarked samples, working to improve learning harmony and enabling the prototype to collaborate for both inter- and cross-label issues, as well as to approximate pseudo labels by a precise ensemble of classification models, in contrast to conventional methodologies that can choose self-assured pseudo label by criterion.

Xue et al. [156] proposed a novel training methodology for effective clinical image categorization from chaotic class labels using domestic and global learning algorithm. To effectively choose cleaner and chaotic data, they use the self-ensemble approach with a messy labeling filter. The proposed approach succeeds better than existing training from chaotic label approaches.

Apostolidis and Papakostas [157] proposed a technique for adversarial assaults have established themselves as a serious danger to machine vision. This study explores the hypothetical black-box challenges and respond aspect of computerized watermarking, which is a separate aspect of the technology. The widespread usage of watermarks for safety purposes appears to offer major hazards to machine vision, which is why the they emphasized a critical issue in this respect in addition to introducing a new class of adversarial assaults called watermarking attacks. The methodology with the greatest decrease was CT scans, while the framework with the greatest vulnerability was MobileNetV2.

Jin et al. [158] proposed a theoretical paradigm for combining the unrivalled advantages of superior human understanding (native intellectual ability) and machine intelligence to create a reliable, precise, and all-encompassing item detection technique for clinical image processing. The Automatic Anatomy Recognition (AAR-DL) method integrates deep learning image recognition systems, model-based object classification and advanced anatomy modeling technique. In each of the four major components that make up AAR-DL, past information is carefully considered. High precision and resistance to image distortions and aberrations have been shown with AAR-DL. AAR-DL exhibits extraordinary resilience and positioning precision within 1-2 grid cells, performing like a skilled human observer in object identification.

In this study, [159] efficiently utilize the entire clinical dataset for semi-supervised clinical image categorization, they present a new mutual consistency network (MC-Net+) in this research. One common encoder and a number of marginally unique decoders are present in the framework. Consequently, our suggested MC-Net+ architecture comprises of two fresh layouts. First, the architecture has a single common encoder and a number of marginally unique decoders (i.e. using various up-sampling approaches). The unidentified difficult sections are indicated by the statistically significant difference of the outcomes from numerous decoders, which is estimated to signify the architecture’s ambiguity. Second, they add a unique reciprocal consistency requirement between the smooth pseudo labels of one parser and the probabilistic outcome of another parser. On three open datasets, they evaluated the categorization outcomes of our MC-Net+ system with five cutting-edge methods.

Wang et al. [160] proposed technique for Diabetic Retinopathy (DR). One of the most serious consequences of hyperglycemia is Diabetic Retinopathy. For an earlier detection of DR, precise separation of DR symptoms is crucial. Technically speaking, concurrent categorization of several DR tumors is difficult due to 1) the dearth of pixel-level labels and 2) the wide variety of DR tumors. They provide a brand-new Poisson-Blending Data Augmentation (PBDA) approach to create fake images that can simply be used to increase the training examples already available for tumor separation. The approach underwent significant validation using comparative and elimination tests on two open clinical datasets. The outcomes showed that the suggested approach significantly improved than the most recent techniques.

Zhao et al. [161] proposed a scientific studies and healthcare diagnostics both heavily rely on Magnetic Resonance (MR) scanning. The location of the layer cluster has a significant impact on the utility of the recovery since MR scanning has a strong in-slice accuracy and a poor through-slice resolution. Conventional medical workflows rely on laborious hand adjustments that are difficult to duplicate. In comparison to our earlier research, a localization network was included, concluding the automated slice grouping positioning operation. To improve the categorization efficiency when processing full resolution images, they develop a multi-resolution architecture. Using variational Weighted Principal Component Analysis (WPCA) regularization they enhance the plane separation technique. They created the Performance Measurement Index, or PMI, that is utilized to show the client how confidence.

Wang et al. [162] proposed a novel methodology for dermoscopic images containing skin lesions. Professionals must put in a lot of research to better understand the features from medical and dermoscopic images in order to diagnose skin lesions accurately. When contrasted to solitary modality-based approaches, deep heterogeneous learning-based techniques can lower intra- and inter-reader heterogeneity and increase classification output. In order to accomplish heterogeneous skin lesion detection, this paper introduces a unique technique called Adversarial Multi-modal Fusion with Attention Mechanism (AMFAM). To be more precise, they utilize an adversarial learning-based discriminator to drive the feature extraction technique to deliberately acquire the linked data. By understanding the associated and supportive data, an unique heterogeneous fusion approach is suggested to conduct automatic skin lesion categorization utilizing medical and dermoscopic images. To direct the feature extraction technique in learning the connected data, a modality classifier is created. An image reconstruction method built on self-attention that autonomously directs the extracted features to focus on tumor regions.

Tilborghs et al. [163] explored the most advanced method for several brain tumor segmentation challenges, such as the separation of the myocardium in cardiac magnetic resonance imaging (MR) images, is semantic segmentation utilizing convolutional neural networks (CNNs). However, the anticipated division maps produced by such a typical CNN would not permit precise comparison of local form characteristics such local layer strength. They offer a CNN for immediate myocardial geometry and posture factor estimation. An appropriate mathematical geometry model relying on landmarks is connected to the parameters. To help with accurate feature estimation and form adaptation, semantic segmentation is utilized. The error function enforces uniformity between estimated attributes and integrated segmentation. The adapted geometry model may be used to instantly determine regional myocardial characteristics.

Yang et al. [164] highlights a basic issue in computational histopathology is the retrieval of pictorial representations. Self-supervised training has evolved as an important method to derive useful pictorial representations from unprocessed histopathology images in light of deep learning’s strong representational capabilities and the dearth of labels. While a few self-supervised techniques have been presented expressly for histopathology images, the majority of them include flaws which may limit their adaptability or capability for depiction. A brand-new multi-modal self-supervised training technique for pictorial imagery designed specifically for H &E stained histopathology images. Self-supervised methodologies that are discriminatory and creative can improve one another. using histopathology-specific area information to make wise choices. Excellent adaptability for various computational histopathology problems.

In this study, [165] discuss and analyze the tissue level semantic segmentation. In computational pathology, tissue-level semantic analysis is a key step. With extensive pixel-level labels, fully-supervised algorithms have indeed demonstrated remarkable achievement. Unfortunately, it is quite costly and time-consuming to create such markers on the giga-pixel entire slide images. With only texture categorization markers as input, they describe a cell semantic segmentation technique for histopathological images that significantly reduces the amount of time pathologists must spend annotating the scans. To close the knowledge gap across patch-level and pixel-level tags, multi-layer pseudo-supervision with continuous dropout attention is suggested. Additionally, a categorization gate function is included to lower the frequency of false positives. On two datasets, our design plan outperforms weakly-supervised semantic segmentation algorithms in terms of effectiveness while also performing on pace with the fully-supervised background. The first LUAD dataset for weakly-supervised tissue semantic segmentation has been made available.

Zhang et al. [166] proposed a novel methodology for Nuclear Cataract (NC) using Optical Coherence Tomography. A major source of vision loss and visual impairment worldwide is nuclear cataract (NC). Strategy for medically early treatment and cataract surgery requires precise and trustworthy NC assessment. The core area may be seen easily in Anterior Segment Optical Coherence Tomography (AS-OCT) images, which can also be used to quantify the transparency of NC. Recent medical studies have shown that there is a strong inter- and intra-class association and recurrence between the averaged nuclei volume on AS-OCT scans and the NC intensity of injury. The medical approaches serve as the inspiration for this article’s basic yet successful Region-based Integration-and-Re-calibration attention (RIR), which combines several Convolutional feature area depictions and softmax attention iteratively readjusts the values of individual part. The network may concentrate on area depictions with high contributions and inhibit those that are less valuable thanks to this region re-calibration technique. To dynamically forecast the threat standard of NC, they integrate the RIR block with the Residual convolution layer to create a Residual-RIR module. A series of Residual-RIR modules are then layered to build the region-based integration-and-re-calibration network (RIR-Net).

Huang et al. [167] proposed concept for breast cancer detection using Ultrasound (US). In particular for women with thick mammary, US is essential for breast cancer prevention. Prior to making the assessment, it is standard procedure for a sonographer to identify the main clinical characteristics of a tumor and capture one or more sample images throughout the active imaging. They put forth a reinforcement learning-based approach that can autonomously retrieve distinguish frame from breast ultrasound movies of variable period in order to tackle these issues. It has a detection-based tumor screening component and a cutting-edge incentives that enable keyframe searches to include topographical and clinical aspects of the tumors.

Yi et al.[168] proposed methodology for nerve segmentation using ultrasonography for anesthesiologists. When administering anesthetic during peripheral nerve blockade, anesthesiologists frequently employ ultrasound-guided injection. Even for seasoned anesthesiologists, it can be a challenge to correctly detect nerve anatomy in ultrasonography. The proper implementation of PNB operations depends on the precise recognition of nerve in ultrasonography. In addition to being a clinical issue, precisely segmenting the nerve anatomy from ultrasonography is a difficult work for machine vision. To enhance the categorization efficiency of nerve anatomy by concurrently segmenting multiple structures in ultrasonography, an unique brachial plexus segmentation model (called MallesNet) combined with past healthcare expertise of nerve recognition is presented.

Huang et al. [169] explored concept of Graph Convolutional Network for tumor segmentation using ultrasound. Breast ultrasound (BUS) has shown to be a reliable method for finding breast malignancy in its initial stages. A tumor categorization is an essential first stage in making an adequate assessment since it identifies the target’s boundaries, form, and position. An expert separation technique that can handle hazy or obscured boundaries in ultrasonography. The framework for discrete edge choosing that can concentrate on the borderline area autonomously. The edge rendering architecture that uses the Graph Convolutional Network (GCN) and can use global contour data. a comprehensive architecture capable of concurrently segmenting and classifying data.

Li et al. [170] discovered a novel concept for Daibetics patients using Vision Transformer and Tongue Diagnosis Analysis System (TDAS). Diabetes is a long-term illness that is prevalent and highly prevalent Globally, especially in China. A serious global healthcare issue is diabetes. However, it is challenging to manage the progression of diabetes with the present diagnostic and therapeutic approaches. Due to its inexpensive price, potent therapeutic impact, and easy availability, traditional Chinese medicine has emerged as a viable choice for the prescription of diabetes. This work introduces a brand-new algorithm for automatically categorizing images of diabetic tongues. The individuals’ tongue images are taken using the TFDA-1 tongue diagnostic tool. The TDAS characteristics of the tongue images are extracted using the Tongue Diagnosis Analysis System. Images of the tongue are used to collect distinguish characteristics using the Vector Quantized Variational Autoencoder (VQ-VAE). K-means clustering of tongue images using VQ-VAE characteristics. The disparities across regions are described using TDAS characteristics. The categorization findings are validated and positional monitoring feedback is calculated using Grad-weighted Class Activation Mapping (Grad-CAM) and Vision Transformer (ViT).

Oyelade et al. [171] proposed a novel concept for histopathological images using Whole-image based CNN (WCNN) and Region-based CNN (RNN). Interpretation and localization of anomalies in clinical images is thought to be a highly difficult process. To mitigate this problem, several computer-aided techniques have been used, and the popularity of deep learning network topologies is evidence of the remarkable contribution that has been documented in the research. In this study, they propose a dual subsidiary deep learning framework that makes use of two alternative Convolutional neural network architectural topologies to handle problems associated with the coupling of categorization with image localization and recognition. To categorize and locate anomalies in collections, whole-image based CNN (WCNN) and region-based CNN (RCNN) models are rigorously mixed. The technique does not need images that are reliant on labeling to classify anomalies into many categories or to pinpoint their location. Additionally, a flawless assurance and reasoning method is offered, allowing the results from WCNN and RCNN to be combined for additional study.

Sadik et al. [172] propose a new paradigm for COVID-19 detection using customized U-Net. Computer-aided diagnostic technologies are becoming increasingly necessary in the Coronavirus disease-2019 (COVID-19) outbreak for the quick and reliable detection of a significant amount of individuals in addition to conventional approaches. In this study, an efficient, precise COVID-19 detection method based on deep convolutional neural networks (CNNs) is suggested using pulmonary computed tomography (CT) scans images. Initially, by adding more skip linkages to the U-Net structure to compensate for the information loss caused by dimension shifting, a customized CNN architecture called SKICU-Net is developed for the detection and segmentation of pulmonary areas in a chest CT scan. Following that, an agglomerative hierarchical algorithm is used to exclude the CT segments with insufficient data. Finally, a customised DenseNet structure called P-DenseCOVNet is configured for efficient feature mining and treatment of COVID-19 and pneumonia from fragmented chest slices. This structure adds concurrent convolution layer pathway on highest part of the standard DenseNet framework to improve effectiveness by conquering the cost of strategical reasoning.

Sun et al. [173] propose early detection mechanism for retinal disorder using Hybrid graph convolution. For the purpose of preventing permanent vision damage, timely identification and management of retinal problems are essential. The creation of multi-label fundus illness recognition systems that can check for numerous illnesses is better in accordance with practical demands considering that individuals in the healthcare environment may have several forms of visual sickness. This study provided a synthesis framework for cross-label fundus ailment diagnosis at the individual level relying on hybrid graph convolution. A foundation component, a hybrid graph convolution subsystem, and a classification subsystem made up the composite model. This study used graph convolution to construct the relationships across the categories, and it then used a self-attention method to create a composite graph convolution architecture. While the classification component produced cross-label data using LightGBM, the foundation component used EfficientNet-B4 for feature extraction. The input data of binocular images and the effect of tag association on the model’s recognition rate were also examined in this study. On the readily viewable ODIR dataset, the design plan MCGL-Net surpassed all other cutting-edge techniques, with F1 achieving 91.60% on the testing dataset.

Liu et al. [174] advise a novel approach for neuroblastoma categorization using histopathological whole-slide images. The most frequent extra-cranial malignant tumor in young children is neuroblastoma. Physicians can utilize the International Neuroblastoma Pathology Categorization (INPC), a widely employed classification approach, as a resource for therapy stratification. To categorize individuals having neuroblastoma into several prognosis categories, an autonomous, extensive, and accurate categorization approach is required. In this research, 107 participants having neuroblastoma who had surgical excision provided 563 Hematoxylin and Eosin-stained (H &E) histopathological whole-slide images. For nuclear separation, cell-level visual feature collection, and individual-level feature accumulation, they suggested an unique computational strategy. To categorize individuals with Favorable Histology (FH) and those with Unfavorable Histology (UH), a logistic regression classifier was created.

Saini and Susan [175] propose a unique model for mulit-class imbalance dataset of Diabetic Retinopathy (DR). Diagnosing and monitoring for diabetic retinopathy is a well notable topic in the biomedical field. A component of Computer-Aided Diagnosis (CAD) that has advanced significantly over the previous several decades as a result of the development and effectiveness of deep learning is the application of medical imaging from a patient’s vision to identify the injury inflicted to blood vessels. Comprehensive comparison of three standard datasets of varying dimensions, each containing images of diabetic retinopathy for DR scoring (identification), separation, tumor recognition, and optical disc, using several state-of-the-art transfer learning approaches. For optimum efficiency when dealing with various unbalanced circumstances of various dataset dimensions, several pre-trained CNN models used to fundus images together with rejected interpolation (randomized under-sampling at mini-batch level) approach.

Xin et al. [176] explore Vision Transformer (VIT) and apply on skin lesion classification using multi-scale and overlapping sliding windows. Current decades have seen significant advancements in the initial detection and medication of melanoma, whose prevalence is rising annually throughout the world and pose potential a serious risk to human health. These advancements have been made possible by the use of machine learning to recognize dermoscopic images. Three stage process is used to confirm its efficacy. In order to confirm the efficiency of SkinTrans in the categorization of melanoma, a VIT system is first constructed. The image is then serialized using cross-scale and overlapping sliding windows, and cross-scale patch implantation is performed with a focus on cross-scale characteristics. In order to create the encoding outcomes of diverse input source as distinct as feasible, contrastive learning is utilized to create the comparable facts of melanoma encode uniquely.

Shabani et al. [177] propose a novel strategy for COVID-19 segmentation using self-supervised learning. Segmenting medical images is an important first phase in many medical strategies. We provide an unique and new labeled-free process to do categorization of COVID-19 Computed tomography. Whereas the majority of autonomous categorization approaches are supervised and need a easily-labeled paired dataset. They suggested a self-supervised approach that may not necessitate any human categorization labeling at the cell level. We created wholesome CT images from COVID-19 Computed tomography using GAN. By doing region-aware categorization, we were able to improve the findings by using a contrastive cost. Our approach behaves reasonably better than the state-of-the-art approaches for segmenting COVID-19 Tomography at this time.

Hayat et al. [178] discover Malaria infection using Genetic Algorithm (GA) and Computational Linguistic (CL) to classify the Plasmodium falciparum. Malaria is a contagious and lethal infection that is brought on by Plasmodium falciparum. Initially, a microscope was used to identify cells that were contaminated with malaria. Due to the sheer volume of data to analyse and the complexity of the timing, it could result in false diagnosis. Increasing time requirements and inaccurate diagnosis have led to a significant need for autonomous parasite diagnosis methods. For the purpose of identifying the malaria parasite, a smart mathematical framework is developed. As extracting features strategies, biochemical, physiochemical, and Computational Linguistic (CL) approaches are employed. The ensemble method is GA-based. The analysis of several identification methods. Support Vector Machine incorporates Bose Chaudhuri Hocquenghem (BCH) loss correcting algorithm to lower loss.

Hussain et al. [179] propose a novel concept for Breast tumor segmentation using embedded U-Net model. For the purposes of evaluating, recognizing, and detecting cancers, breast tumor separation in B-mode ultrasound imaging is crucial. The degree setup strategy is one that is quite frequently employed for breast separation, and it is continually being refined. In order to utilize thematically augmented characteristics for breast tumor separation, this research suggests a brand-new deep-feature integrated level set block. A UNet-based model is initially trained to retrieve various characteristics at various phases. Every level depicts distinct aspects differently. Furthermore, at the conclusion of every phase, an unique level-set mechanism is added to acquire better detailed and realistic feature maps. In the performance component of the level-set technique, a novel feature-discriminator is developed to improve the low confidence values at the borders. Finally, to strengthen the separation procedure even more, the outcomes of the level-set approach at multiple phases are merged into the resulting feature maps.

Sendra-Balcells et al. [180] adopt domain generalization concept for MRI scans. For non-contrast scanning throughout the past few decades, the domain generalization issue has been extensively researched in machine learning, while for contrast-enhanced scanning, it has gotten less interest. In this article, they give a thorough analysis of deep learning methods for contrast-enhanced image separation that may be generalized to unknown healthcare situations. In order to accomplish this, a number of techniques including data preprocessing, domain combining, transfer learning, and domain adaptation are researched, optimized, and rigorously assessed. The techniques are assessed for ventricular separation in contrast-enhanced cardiac MRI scans to show the possibility of domain generalization for contrast-enhanced scanning.

Yalçın and Vural [181] explore a new concept for brain injury using customized U-Net CNN model. For doctors to concentrate on certain parts of the brain and provide individuals the appropriate therapy, precise brain injury assessment, categorization, and separation are crucial. Numerous artificial intelligence technologies have successfully included encoder-decoder deep learning techniques.The Computed Tomography (CT) scans from the medical dataset employed to detect if a brain injury has occurred are examined by the suggested approach. Once an injury has happened, it is possible to ascertain whether it was triggered by ischemia or bleeding. The suggested approach can also perfectly separate the current stroke and highlight the area that the physician has overlay.

Qin et al. [182] proposed a novel concept for Airway Tree Segmentation using convolutional neural network (CNN). It is essential for bronchial illness detection and endobronchial navigation that the airways are segmented on CT images. The challenging construction and varied morphology of the airway need laborious hand-crafted extraction attempts. Convolutional neural network (CNN) oriented techniques have lately emerged as the most advanced technique for autonomous airway segmentation. Nevertheless, it is still difficult for convolutional neural networks to understand the inter-connectedness of the airways and recognize the tree-like layout. In order to solve this problem, we provide AirwayNet, a voxel-connectivity informed technique for precise airway identification.

Lian et al. [183] proposed a novel concept for Computer Tomography (CT) scans for lungs tumor classification using Tumor-CNN. Appropriate patient lifespan forecasting can help with medication management and may even boost performance because pulmonary disease is the most common cancer-related reason for death. In this research, utilizing Computed Tomography (CT) scans from individuals with Non-Small Cell Lung Cancer (NSCLC), we developed an autonomous method competent of pulmonary separation and mortality forecasting. In this retrospective investigation, we constructed unique pulmonary graphs from the 10 chest Computed Tomography scans segments and trained a GCN algorithm to estimate 5-year mortality risk. The present Tumor Nodes Metastases (TNM) staging system was compared to a series of machine learning algorithms, a convolutional neural network based on tumors (Tumor-CNN), and a Cox proportional-hazard predictor.

Zheng et al. [184] propose a novel concept for pre-operative diagnostic and intra-operative positioning for lung treatment, autonomous airway separation is a requirement. This is hindered by a significant imbalanced data across backgrounds and foreground parts, which finds it difficult for CNN-based algorithms to interpret distal minor airways due to the tiny dimension and dispersed spatial patterns of peripheral bronchi. In this study, we show how the neighborhood voxels’ gradient degradation and elongation cause this issue. The salient gradients may be degraded by their neighborhoods throughout back-propagation if the frontal slope to overlay slope proportion is low and the imbalanced data is regional.

Zhang et al. [185] use 3D CNN for airway segmentation. For airway segmentation, 3D convolutional neural networks (CNNs) are frequently used. The medical datasets has a significant impact on how well 3D CNNs work, however the available airway datasets are primarily pristine Computed Tomography (CT) images with limited labeling, making it challenging to generalize to noisier COVID-19 CT scans. In this study, they suggest a novel dual-stream system that employs pristine Medical images and a tiny handful of labeled noisy Computed tomography for airway segmentation to solve the heterogeneity between the clean region and noisy region. To differentiate the distinctive noisy characteristics from the shareable clean characteristics, they create two distinguish encoders, accompanied by two distinct decoders.

In conclusion, CNNs are the prevailing industry norm for testing categorization. CNNs in particular have produced remarkably good outcomes after being trained on genuine images, surpassing the competence of human specialists in several applications. The researchers have also demonstrated how CNNs may be modified to take advantage of the inherent organization of medical images.

## Anatomical Domains of Medical Images

An summary of the achievements made by deep learning towards the different healthcare imaging technology fields is provided in this section. We analyse machine efficiency on big data sets and on challenge data sets, and we emphasize certain valuable discoveries. On the website http://www.grand-challenge.org, these problems are all mentioned.

### Lungs or Chest

The most frequently discussed topic in pulmonary image processing from ct imaging and radiography is the identification, description, and categorization of tumors. Numerous studies evaluate CNNs with traditional machine learning techniques employing handmade features or supplement current feature sets with deep network-derived features. With an unified technique, numerous organizations may identify various illnesses with a lung X-ray. Another well-liked study area in CT is the identification of topographical features suggestive of interstitial pulmonary disorders [186–188].

The most frequent radiological examination is a chest X-ray, and various studies have used a huge collection of images and textual descriptions to build algorithms that integrate CNNs for visual interpretation and RNNs for textual processing. In the foreseeable future, we anticipate seeing additional study in this area [189–191].

All of the highest approaches in a current competition for tumor identification in CT, LUNA16, utilized CNN frameworks. In comparison, ANODE09’s chest tumor identification challenge included handmade characteristics to categorize potential nodules. The top systems in LUNA16 continue to employ nodule possibilities calculated by rule-based image analysis, while deep network candidacy identification techniques also delivered excellent results (e.g. U-net). The 2017 Kaggle Data Science Bowl, with $1 million in awards and also more than 1,000 competing groups, has as its goal predicting the likelihood that a person has lung disease from a Computed Tomography Scans [192–197]. COVID-19 Lung CT Lesion Segmentation Challenge - 2020 organized by MICCAI and more than 1976 teams are participating to predict the COVID-19. Airway Tree Modeling (ATM22) Challenge 2022 organized by MICCAI2202 and more than 288 teams are participating to predict pulmonary disease diagnosis and endobronchial navigation.

**Table 2:** Selected articles of brain MRI using deep learning

Author	Details
Ragab et al. [198]	Two-phased segmentation approaches are used for breast tumor segmentation
Mambou et al. [199]	Used Independent Component Analysis (ICA) with convolutional neural network to classify breast cancer
Selvathi and Poornila [200]	Used Sparse-autoencoder, Stacked Sparse-autoencoder with Convolutional Neural Network for mammogram classification of breast cancer
Mohamed et al. [201]	Used multi-fold based technique for breast cancer on biopsy dataset
Kavitha et al. [202]	For diagnosing digital mammogram of breast cancer, they used Optimal multi-level Thresholding based segmentation with capsule network
Chowdhury et al. [203]	Used transfer learning approach with customized CNN and ResNet101 for classification of breast cancer
Escorcia-Gutierrez e al. [204]	Used CNN model with ResNet34 with distinct preprocessing steps for classification of breast cancer
Jasti et al. [205]	Used various distinct approaches for feature extraction, selection, image processing and classification of mammograms
Jabeen et al. [206]	Used five-fold based deep learning approaches for classification of breast tumor classification from ultrasound
Naseem et al. [207]	Used various machine learning based ensemble algorithms to classify breast tumor
Singh et al. [208]	Proposed a hybrid approach comprises residual and inception block of CNN for breast cancer classification
Liu et al. [209]	Used a pre-trained CNN model AlexNet and fine-tuned on BreakHis, IDC and UCSB datasets
Wang et al. [210]	Developed a novel deep learning approach DeepGrad model comprises InceptionV3 blocks for Histopathological image classification
Reshma et al. [211]	Used Fourier Transform based Segmentation for Histopathological image (Biopsy) classification of breast cancer
Ragab et al. [212]	Proposed Ensembled based deep learning approach containing multi-level thresholding based segmentation for breast tumor
Ahmad et al. [213]	Used Gated Recurrent Unit with pretrained CNN model (AlexNet) for classification of Lymph Node of breast tumor
Maqsood et al. [214]	Used multi-phase approach comprises contrast enhancement, Transferable texture using pretrained CNN models
Ibrokhimov and Kang [215]	Proposed two-stage CNN network to extract local patches from breast cancer and locate Region of Interest (ROI)
Mohamed et al. [216]	Used two-step approach, comprises of U-Net (CNN) for extraction fo breast from the whole body and second step is to classify into binary classes

### Digital Pathology (Histopathological Image) and Microscopy

Deep learning algorithms are increasingly being used in the field of digital pathology and microscopy due to the increasing accessibility of massive gigapixel Whole Slide Imaging (WSI) of cell samples. The approaches that have been created and used in this arena concentrate on three primary problems: Segmenting large organs, identifying and defining the condition of relevance at the tumor- or WSI-level and finding malignant cells on those regions.

Images from histology have also been normalized using deep learning algorithms. An essential topic is Color Normalization in the research of histopathology image processing. A technique for stain normalization of Hematoxylin and Eosin (H &E) stained tissue samples utilizing deep sparse auto-encoders was proposed by [217]. Janowczyk and Madabhushi [140] briefly demonstrated the value of color normalization for CNN-based tumor categorization in H &E colored images.

Digital pathology has faced some significant obstacles, which has aided in the growth of computerized digital pathology methods. AMIDA 2013 Mitosis detection challenges sponsored by MICCAI Grand Challenge ICPR 2012 Contest on Mitosis Detection, EM segmentation challenge (ISBI - 2012) for the 2D segmentation of neuronal processes, GLAS for gland segmentation CAMELYON16 and TUPAC for processing breast cancer tissue samples are among the challenges that assessed both established and novel methods for analyzing digital pathology images [218–220].

In clinical specimens from colon tumor patients, the challenge of gland instance segmentation was handled by GLAS. The highest rank was attained by [221] using three CNN architectures. Pixels are categorized as gland or non-gland in the initial CNN. The comprehensively layered edge approach, which creates an edge map via side convolutions, is used to retrieve edge detection out of each feature space of the initial CNN. Lastly, the complete segmentation is created by merging edge and gland patterns in a final CNN architecture [222].

The IDSIA research group surpassed rival methods with a CNN-based strategy in both the ICPR 2012 and the AMIDA13 contests on mitosis recognition by [223]. For the 2-dimensional segmentation of neuronal networks at EM 2012, the very identical team’s approach [224] obtained the best performance. As part of their method, a CNN’s outcome was lightly smoothed and threshold-ed to accomplish the goal of dividing the membranes of cells.

The inaugural contest to deliver competitors WSIs was CAMELYON16. In contrast to certain other medical image processing, the vast amount of labeled data that was available in this competition permitted for the development of extremely deep models, such as the ResNet-101, VGG-Net-16, GoogLeNet-22 utilized by [91] and [94]. Each of these architectures was utilized by the top five devices. The CAMELYON16 challenge’s top-performing approach was described in [225] The approach is built on an ensemble of two GoogLeNet architecture, one learned using hard negative mining and the other without it. AUC of 99.35 was obtained for job 2 in the most recent response from this research group utilizing the WSI standardization technique by [226], outperforming the AUC of a clinician who individually evaluated the entire test set (AUC = 96.6).

The most current TUPAC competition focused on predicting tumor classification at the Whole Slide Imaging (WSI) level and detecting mitosis in breast malignant tissue. The best solution was developed by [227], and it excelled at every challenge. The technique consists of three basic parts: Identifying areas with high cell numbers, using a CNN to identify mitoses there, turning the mitosis identification findings into feature vectors for each Whole Slide Imaging (WSI), and employing a Support Vector Machine (SVM) classifier model to calculate malignant growth and molecular information ratings are the next steps.

**Table 3:** Selected articles of Diabetic Retinopathy (retinal fundus images) using deep learning

Author	Details
Li et al. [228]	Used deep ensemble algorithm for to detect Diabetic Retinopathy (DR) using retinal fundus images
Pinedo-Diaz et al. [229]	Proposed a unique lightweight Convolutional Neural Network for detection of diabetic retinopathy using retinal fundus images
Saranya et al. [230]	Used pretrained CNN model (VGG-16) for classification of neovascularization using color fundus images
Boreiko et al. [231]	Proposed an adversarially robust ensemble model containing visual counterfactual explanations, Saliency maps (Integrated Gradients (IG) and Guided Backprop (GBP)) using retinal fundus images
Gunasekaran et al. [232]	Identified a novel model using deep recurrent neural network for classification of diabetic retinopathy
Saranya et al. [233]	Used a pretrained CNN block (DenseNet) for classification of daibetic mellitus
Mikram et al. [234]	Discovered a hybrid approach using distributed and non-distributed technique deep feature extraction and classification
Gao et al. [235]	Used a pretrained CNN models such as VGG16, RestNet50, and DenseNet for screening fundus fluorescein angiography (FFA) images
Abbood et al. [236]	Used a novel two-stage approach comprises of cropping, removing noise (gaussian blur), contrast enhancement and ResNet50 for classification of diabetes mellitus
Nneji et al. [237]	Used Contrast-Limited Adaptive Histogram Equalization (CLAHE) and Contrast-Enhanced Canny Edge Detection (CECED), to enhance the low quality images into high quality
Dayana and Emmanuel [238]	Used various preprocessing techniques for removal of noise, contrast enhancement and deep attention based Fusion Network to classify DR stages
Zhang et al. [239]	Developed a novel CNN model containing InceptionV3 characteristics for diagnosing severe Diabetic retinopathy
Canayaz [240]	Acquired 512 features using EfficientNet and DenseNet models and classify those features via Support Vector Machine
Venkaiahppalaswamy et al. [241]	Used pretrained CNN models namely AlexNet and GoogleNet for classification of retinal fundus images
Gupta et al. [242]	Used guided and median filter in processing stage and employed U-Net for infection segmentation
Granty Regina Elwin et al. [243]	Proposed a novel Autoregressive-Henry Gas Sailfish Optimization (Ar-HGSO) based deep learning model for Macular Edema classification
Nderitu et al.[244]	For retinal and gradability classification using customized deep learning approach
Desika et al. [245]	Used Wild Geese Algorithm with customized Region-based Convolution Neural Network (R-CNN)
Islam et al. [246]	Used CLAHE and Xception based CNN model is used for detection of diabetic retinopathy
Babenko et al. [247]	Proposed a novel deep learning model for classification of diabetic macular oedema and poor blood glucose control using retinal fundus images

### Breast

Breast tomography was one of the initial areas where [121] applied DNN. Subsequently, research has resurfaced, leading to considerable advancements over the state-of-the-art and obtaining ROI effectiveness comparable to that of sentient readers [248]. Although typical breast imaging approaches are two directional, strategies that work well with real images can be simply applied to artificial imaging.

Including one exemption, there are only three objectives that are acknowledged: (1) the identification and categorization of mass-like tumors, (2) the identification and categorization of microcalcifications, and (3) the image-based breast tumor probability assessment. The modality that receives the maximum utilization and, as a result, the maximum interest is mammography. There is presently little research on tomosynthesis, US, and tidal pulse elastography, and there is just one publication that used deep learning to evaluate breast MRI. These additional modalities will certainly get increasing emphasis in the next decades. The literature’s key points are summarized in Table 2.

While several nations have breast tumor monitoring programs, there ought to be tonne of data accessible, particularly for mammography, and consequently sufficient room for deep CNN models to thrive. Outdated digitized screen-film data sets are still being used because huge public digitized libraries are regrettably inaccessible. Competition like the just-launched MItosis DOmain Generalization Challenge 2022 (MIDOG 2022), Tumor InfiltratinG lymphocytes in breast cancER (TIGER) and Breast Cancer Immunohistochemical Image Generation Challenge (BCI) challenge haven’t yet achieved the results expected.

Numerous research examined limited data sets as a response, which had varying results. This problem has been explored in a number of studies by investigating transfer learning [249, 250], semi-supervised learning [251–253] and weakly supervised learning [254–256]. Some other approach integrates customized features with cnn networks, which has been demonstrated to be complimentary even for extremely large amounts of data [257–259]. Modern methods for classifying mass-like tumors often use a multiple workflow with a candidate sensor; this architecture condenses the image to a list of possibly cancerous tumors that are given to a deep CNN [260–264].

To avoid the cascaded method by using a region-based proposal network (R-CNN) [265–267]. Excellent outcomes can be attained when there are massive data sets accessible. A research scientist from a top breast imaging CAD organization described to a crowded meeting hall at the SPIE Medical Imaging conference in 2016 how just a several times of experimental studies with a basic deep CNN architecture (AlexNet) prepared on the business’s patented technology database produced effectiveness that was better than what decades of designing manual feature structures had produced [268].

### Eye

Although ophthalmic scanning has advanced significantly in previous decade, deep learning techniques have just gradually been used to comprehend eye images. The majority of studies use straightforward CNNs to analyze Color Fundus Images (CFI), as seen in Table 3. Techniques for segmenting anatomical features, detecting and segmenting retinal anomalies, diagnosing eye disorders, and evaluating image resolution are all covered.

In order to create systems to determine the extent of the infection in 53,000 trial images, over 35,000 colour fundus scans were supplied in a diabetic retinopathy detection challenge conducted by Kaggle in 2015. The bulk of the 661 groups that participated in the challenge used deep learning, and four, all of which used end-to-end CNN model, outperformed individuals in terms of outcomes. A Google Inception v3 model just underwent a rigorous investigation by [144] in order to identify diabetes mellitus, and the results showed accuracy on par with a board of seven board-certified ophthalmologists.

The top-performing groups to date have all employed CNNs in the 2022 and 2021 challenges such as Diabetic Retinopathy Analysis Challenge (DRAC-2022) sponsored by MICCAI 2022, RAVIR: A Dataset and Methodology for the Semantic Segmentation and Quantitative Analysis of Retinal Arteries and Veins in Infrared Reflectance Imaging 2022 [269], AIROGS: Artificial Intelligence for RObust Glaucoma Screening Challenge sponsored by IEEE ISBI 2022 [270, 271].

**Table 4:** Selected articles of brain MRI using deep learning

Author	Details
Havaei et al. [272]	Tumor segmentation; two-path way CNN with different receptive fields
Moeskops et al. [273]	Tissue segmentation; CNN trained on multiple patch sizes
Ghafoorian et al. [274]	Lacune detection; FCN for candidate segmentation then a multi-scale 3D CNN with anatomical features as false positive reduction CNN
van der Burgh et al. [275]	Survival prediction; DBN on MRI and fusing it with clinical characteristics and structural connectivity data
Kamnitsas et al. [276]	Tumor segmentation; 3D multi-scale fully convolutional network with CRF for label consistency
Kleesiek et al. [277]	Brain extraction; 3D fully convolutional CNN on multi-modal input
Ghafoorian et al. [278]	Lesion segmentation; CNN trained on non-uniformly sampled patch to integrate a larger context with a foviation effect
Havaei et al. [279]	Tumor segmentation; CNN handling missing modalities with abstraction layer that transforms feature maps to their statistics
Dou et al. [280]	Microbleed detection; 3D FCN for candidate segmentation followed by a 3D CNN as false positive reduction
Shi et al. [281]	AD/MCI/HC classification; multi-modal stacked deep polynomial networks with an SVM classifier on top using MRI and PET
Bashir et al. [282]	Using Stationary wavelet transform (SWT) and Principal Components Analysis (PCA) for Brain image fusion
Panigrahy et al.[283]	Using a Weighted Parameter Adaptive Dual Channel PCNN
Yazdan et al. [284]	Using multi-scale CNN for robust classification of brain tumor and eliminate the affect of Rician noise
Wahlang[285]	Utilizing pretrained CNN model for feature extraction and use traditional classification algorithm SVM to classify Magnetic Resonance Imaging (MRI) for brain tumor classification
Alanazi[286]	Use 22-layer custom CNN with transfer learning for multi-classification of brain tumor
Bangare [287]	Use Modified Region Growing, Gabor filtering are used and Fuzzy Min-Max Neural Network is used for classification
Siddiqi et al. [288]	Use Stepwise Linear Discriminant Analysis (SLDA) with Support Vector Machine
Ouchicha et al. [289]	Use customized dense block with pretrained CNN model for Alzheimer’s Disease Classification Using Brain MRI
Haq et al. [290]	Three step methodology is proposed (1) initially extract pertinent features of brain MRI space. (2) for localizing tumor area use a faster region-based CNN (3) conventional and traditional CNN algorithm is used to categorize and segment the brain tumor
Saurav et al. [291]	Constitutes a novel lightweight attention-guided convolutional neural network (AG-CNN) with skip connection classification of brain tumor using MRI images
Nayak et al. [292]	Propose a parametric based metaheuristics methodology is used with CNN model to classify brain tumor using MRI

### Brain

Neuroscience image processing using DNNs has been widely employed in a variety of professional sectors Table 4. Numerous investigations categorise Alzheimer ’s dementia and divide up the brain’s cell and structural elements (e.g. the hippocampus). The identification and segmentation of diseases are additional crucial regions (e.g. tumors, white matter lesions, lacunes, micro-bleeds).

The majority of approaches train projections from regional portions to representations and then from abstractions to categories, with the exception of those that seek for a scan-level categorization (such as Alzheimer assessment). But for applications where anatomical knowledge is crucial, the regional portions could not have the contextual details needed (e.g. white matter lesion segmentation). Ghafoorian et al. [274] employed non-uniformly observed patches to address this issue by significantly reducing sampling frequency on patch ends to encompass a wider context. Multi-scale investigation and the synthesis of images in a fully-connected layer are alternate methods that are employed by numerous entities. Despite the fact that all of the investigated research used 3-dimensional volumes of brain imaging, the majority of approaches only analyze the 3-dimensional volumes slice-by-slice. The thin slices compared to in-plane sharpness in specific data collections or the decreased computational resources are frequently the driving forces behind this. Additionally, more published studies had used 3D networks.

Several issues in brain image processing have been totally replaced by Cnn architectures. The top-performing groups to date have all employed CNNs in the 2014 and 2015 tumor segmentation challenges (BRATS), 2015 longitudinal multiple sclerosis lesion segmentation challenge, 2022 Ischemic Stroke Lesion Segmentation Challenge (ISLES’22), CuRIOUS 2022 Segmentation Challenge (comprising two major task (1) brain tumor segmentation using intra-operative US (2) cavity segmentation using intra-operative US), automatic segmentation of Head and Neck (H &N) for primary and lymph nodes (HECKTOR challenge in 2020, 2021 and 2022), Surface Learning for Clinical Neuroimaging (SLCN 2022) sponsored by MICCAI2022 [293–295], Anatomical Tracings of Lesions After Stroke (ATLAS R2.0) [296] and 2013 MR brain image segmentation challenge (MRBrains). The majority of the proposed techniques focus on brain MRI imaging. We anticipate that deep learning evaluation will also be beneficial for other brain scans modalities like CT and US.

**Table 5:** Selected articles of Abdomen (CT/MRI/Colonoscopy) using deep learning

Author	Details
Lu et al. [297]	Competing on SILVER07 using 3D CNN and produce plausible outcomes
Ravishankar et al. [298]	Using transfer learning, tuning of existing model and hybrid approach to acquire credible findings
Roth et al. [299]	Using random forests and two CNNs, we can distinguish the pancreas’ inside and exterior
Näppi et al. [300]	In our approach, digital endoluminal imaging of the potential polyps were used to recognize polyps in response to a computer-aided detection (CADe) methodology. The pretrained CNN models are used with millions of natural images
Ren et al. [301]	The unique Computed Tomographic Colonography based CAD technique that is proposed in this study uses three- dimensional radiomic characteristics to provide excellent identification specificity at low FP rate. To provide prospects for polyps, we apply our prior shape-based techniques
Uemura et al. [302]	They built a 3-dimensional Convolutional Neural Network oriented on a flow-based generative model. Using our medical Computed Tomographic Colonography case data, the 3-dimensional Glow was programmed to generate artificial Volumes of Interest of polyps
Yasuda et al. [303]	A simulation experiment was conducted before this one in order to determine the best procedure for ultra-low-dose Computed Tomography Colonography (CTC). 206 individuals were enlisted who had a significant or medium risk of developing colorectal cancer. After completing complete bowel preparation, the individuals were examined in the upright and supine postures using an adaptive incremental reconstruction technique with the Computed Tomography settings set to 120kV, standard error 45 to 50
Younas et al. [304]	In contrast to transfer learning, this research examines six previous pretrained Convolutional neural networks models and chose the best outperforming architecture exclusively for traditional ensemble structures
Tanwar et al. [305]	The colonoscopy images are primarily enhanced and filtered using dynamic histogram equalization and directed image filtering techniques. Then, colorectal polyps in colonoscopy images are accurately detected and classified using Single Shot MultiBox Detector. Lastly, the polyp categories are categorized using fully linked layers with dropouts
Wesp et al. [306]	To estimate the polyp category, two deep learning networks, Segmentation (SEG) and noSEG, were learned using 3D CT colonography image sub-volumes. System SEG was also learned using polyp segmentation markers
Biffi et al. 307]	In order to perform in real-time using a traditional white light (WL) endoscopic video stream without the use of simulated chromoendoscopy, researchers created a revolutionary sophisticated medical equipment (blue light, BL). On a systematically obtained dataset of unmodified colonoscopy recordings, researchers investigated the stand-alone effectiveness of this computer-aided diagnosis tool (CADx) in this research
Sánchez-Peralta et al. [308]	The initial step is to describe the components needed for implementing supervised DL techniques for Colorectal Polyps in colonoscopy. The system design, collection, Error function, data enrichment, and indicators are highlighted. While covering techniques for polyp identification, positioning, separation, and categorization, the publically accessible datasets that may be beneficial for future research are discussed
Kusters et al. [309]	Confidence calibration directly mentions the trustworthiness of a Convolutional Neural Network (CNN) for identification of Colorectal Polyps in this research. Well-calibrated algorithms generate categorization ratings that accurately represent the model probability of being accurate, enabling accurate forecasts with dependable and illuminating confidence ratings
Byeon et al. [310]	Every diseased disc was digitally captured in order to fine-tune two Convolutional neural networks that had already been developed
Nisha et al. [311]	Using a DP-CNN to categorize polyp and non-polyp scan from the colonoscopy images
Cao et al. [312]	To extract polyps descriptors with the help of the gradient operator and Hessian operator

**Table 6:** Selected articles of Musculoskeletal (CT and MRI) using deep learning

Author	Details
Harini et al. [313]	Using pre-trained CNN models such as Xception, Inception, VGG-19, DenseNet, and MobileNet to analyze Musculoskeletal Radiographs (MURA) data set
Kamiya 314]	Analysis of Whole body Computed Tomography (CT) scans using distinct image features such as area, shape, volume and texture
Kijowski et al. [315]	Using MRI and CT scans to detect lesion and forecasting of musculoskeletal illness
Mall and Singh [316]	Proposed a customized CNN (ChampNet) with multi-phase training to classify musculoskeletal using X-ray images
Singh et al. [317]	Discovered a hybrid CNN ComDNet-512 containing three steps training for finger binary classification
Cheng et al. [318]	Identified a eminent resolution based Generative Adversarial Network to generate and enrich the network training of Musculoskeletal Ultrasound (MSUS)
Zhu et al. [319]	To segment inter- and intra-slice features of Cerebral Palsy (CP) using end to end attention based hybrid approach
Shin et al. [320]	To diagnosing musculoskeletal disorders such as meniscus using Convolutional Neural Network
He et al. [321]	Discovered Musculoskeletal model (MSM) to acquired from Electromyography (EMG) to predict joint angle of human body
Malik et al. [322]	Proposed a three-phase hybrid CNN model for classification of elbow fracture
Gitto et al. [323]	Atypical Cartilaginous tumour (ACT) and chondrosarcoma ($\hbox {CS}_{2}$) of long bones classification using MRI + Radiomics classification
Luong et al. [324]	Used transfer learning technique for binary classification of MUsculoskeletal RAdiographs (MURA) dataset
Gao et al. [325]	Used Modic Changes (MCs) for diagnosing MRI with $\hbox {T}_{1}$- and $\hbox {T}_{2}$-weighted using V-Net
Fabry et al. [326]	Used MRI for discriminating Facioscapulo-Humeral Dystrophy (FSHD1) and myositis for visual grading

### Musculoskeletal

Deep learning techniques have recently successfully employed to dissect and identify bone, ligament, and accompanying tendon anomalies in musculoskeletal images from different imaging techniques. Table 6 provides a summary of the publications. There are a remarkable number of finished implementations with encouraging outcomes; one that sticks out is [327] approach, which they learned with 12K discs and promised to execute nearly as well as humans on four separate radiological assessment challenges.

### Abdomen

The majority of abdominal publications focused on localizing and segmenting various parts, primarily the liver, kidneys, bladder and pancreas Table 5. Partition of liver tumors is covered in two studies. For prostate examination, MRI is the primary modality, while CT is used for all other parts. Only in the colon are different techniques discussed, but they were generally done simply: a CNN was employed to extract features, and these characteristics were then used to classify data [328].

It is important to highlight that relatively conventional image processing techniques predominated up to 2016 in two segmentation challenges, SLIVER07 for the liver and PROMISE12 for the prostate. The present second and third-place autonomous algorithms in PROMISE12 used active appearance algorithms. For approximately five years, IMorphics’ approach held the top spot (now ranked second). Meanwhile, [329] a 3D fCNN that is comparable to U-net just overtook the top spot. This study employs a novel method by combining the ResNet and U-net architectures by using a sum procedure in place of the composition process employed in U-net. Convolutional networks have also begun to compete at the top of the rankings in SLIVER07, a 10-year-old liver segmentation competition, replacing previously dominating approaches centered on structure and aspect modeling in 2016.

**Table 7:** Selected articles of Cardiac (CT/MRI/US) using deep learning

Author	Details
Lu et al. [330]	In this paper, SparseVoxNet, a brand-new and highly efficient U-Net-based 3-dimensional sparse convolutional model, is proposed. Any two layers that have the identical feature size have deep links in this network, which decreases the quantity of interconnections
Song et al. [331]	Utilizing different deep learning techniques such as Recurrent Neural Network, Long-Short Term Memory (LSTM), Boltzmann Machines and U-Net for cardiac MRI segmentation
Wang et al. [332]	A Multi-scale Multi-skip connection Network (MMNet) is suggested to completely outsource the left cardiac ventricular separation of cardiac magnetic resonance imaging (MRI) data, influenced by the potential of Convolutional Neural Network
Sandooghdar and Yaghmaee [333]	Using U-Net for segmentation process and random forest classifier for classification
Ahmad et al. [334]	Using ensemble method 2-dimensional residual network with Atrous Spatial Pyramid Pooling (ASPP) for cardiac MRI segmentation
Alabed et al. [335]	This research uses machine learning to evaluate the predictive cardiac magnetic resonance characteristics in Pulmonary arterial hypertension
Fernández-Llaneza et al. [336]	Using Customized U-Net model for evaluating systole and diastole phases (2MSA) and another model trained for all phases(1MSA)
Popescu et al. [337]	3-stage neural network is trained to identify and segment ROI of left ventricle (LV)
da Silva et al. [338]	This solution employs a feedforward strategy that combines FCNs and image analysis methods
Arian et al. [339]	using traditional machine learning and radiomics algorithms to predict myocardial function in cardiac MR (LGE-CMR)
Corrado et al. [340]	The separation of 4D flow velocity data was then applied using the bSSFP images that had been mapped to the appropriate 4D flow images
Lalande et al. [341]	Using inverted Residual Blocks with U-Net for assessment of myocardial infarction

### Cardiac

The research is compiled in Table 7 and shows how deep learning has been employed in several parts of cardiac image processing. Although left ventricle categorization represents the most frequently performed challenge in MRI and the methodology that has received the most investigations, there are many other implementations that can be used, including segmentation, monitoring, slice categorization, visual performance evaluation, automated calcium grading, coronary alignment monitoring, and super-resolution.

The majority of research employed straightforward 2-dimensional Convolutional neural and examined the 3-dimensional and frequently 4-dimensional data scan by scan;  [342] develop an exception framework using 3D CNNs. Four publications use Deep Belief Networks, however they all come from the exact same author team. The Deep Belief Networks are incorporated into complicated segmentation architectures and are only employed for extracting features. Due to their integration of Convolutional Neural Networks and Recurrent Neural Networks, the following two publications stand out: In order to separate the left ventricle scans by scans and retain what characteristics to recall from the earlier scans when separating the subsequent one,  [343] developed a recurrent connection inside the U-net framework.  Kong et al.[344] performed sequential regression to detect particular images and a cardiac pattern using an architectures containing a two - dimensional CNN and a Long short - term memory. Readily viewable data are used in numerous articles. The 2015 Kaggle Data Science Bowl was the biggest challenge in this area, with the objective of autonomously measuring end-systolic and end-diastolic contents in cardiac MRI. The top group in a competition with 192 groups for a winnings of $20,000 each used deep learning, specifically fCNN or U-net categorization techniques.

## Workflow

### Data Preparation

Several procedures must be followed prior healthcare data can be utilized to create an deep learning based algorithm or application. Before using healthcare data to construct a scientific or industrial artificial intelligence based system or application, the appropriate regional ethical committee permission is generally necessary. An organizational regulatory committee must assess the potential and client rewards of the trial. Available data are frequently utilised, necessitating a retrospective analysis. Formal written permission is typically omitted considering the individuals in this kind of research are not required to perform any further treatments. In medical studies, each lead researcher may be required to give consent before sharing patient data. When research data are collected continuously, as in a controlled trial, written permission is required. After receiving ethical permission, it is necessary to acquire, query, correctly de-identify, and safely archive pertinent data. Both the Digital Imaging and Communications in Medicine (DICOM) metadata and the images must be cleared of any sensitive healthcare facts [345, 346].

Particularly when building industrial methods, Intelligence scientists frequently do not even have immediate connection to healthcare imaging data via the Picture Archiving and Communication System (PACS) because they are not generally housed inside a radiology clinic or hospital. Only authorized experts such as medical practitioners, doctors, technologists, PACS managers, and medical scientists are allowed accessibility to PACS systems. Providing healthcare data available to deep learning scientists are complex and takes numerous stages, particularly de-identification of data. The optimal strategy is interaction between physicians and deep learning programmers or developers, either in-house or via collaborative academic agreements [347].

**Fig. 24:**
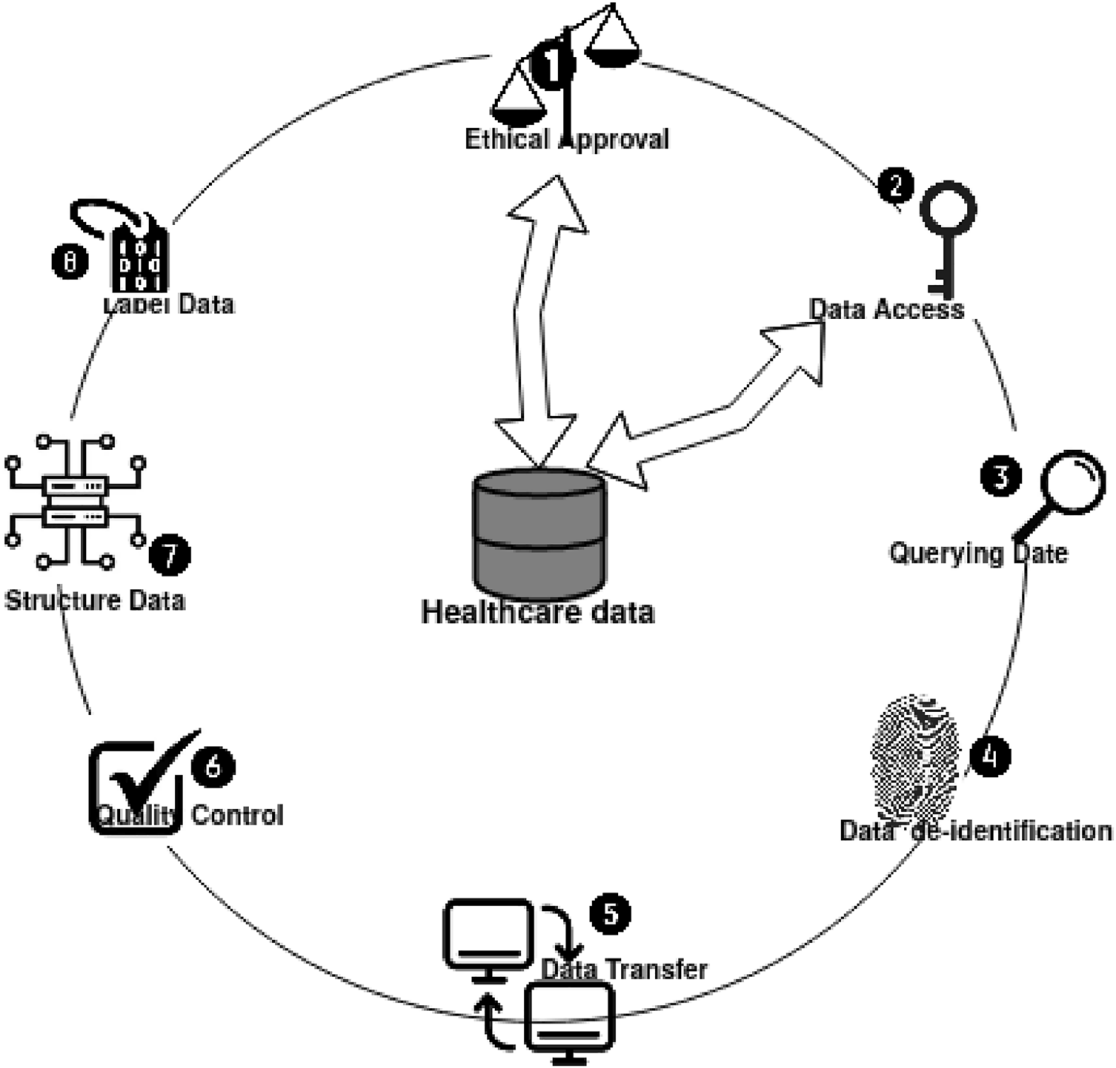
Management of health care data cycle

Even though the U.S. does not usually require individuals to provide formal documented permission, both prospectively and retroactively collected data must be properly de-identified in order to comply with the Health Insurance Portability and Accountability Act (HIPAA), or and the European General Data Protection Regulation. Among other things, confidential material may contain your name, your medical record number, and your birth date. Personal data is frequently contained in the DICOM metadata (header), and there are numerous methods accessible to effectively eliminate this material [348].

The last method for anonymizing health data is k-anonymity, which modifies an initial information source including private healthcare data in order to keep prospective hackers from discovering the identify of the individual. The DICOM information is frequently totally erased or transformed to some other standard, such as NIFTI (Neuroimaging Informatics Technology Initiative), which simply keeps track of voxel size and participant position, when sharing radiology images in open-source scientific projects. While eliminating all DICOM metadata from open-source scientific projects addresses privacy concerns, doing so lowers the quality of the information because metadata is essential for the creation of AI algorithms [349].

### Format

One of the most challenging aspects of processing healthcare images is understanding the image data format. The main file formats now employed in medical imaging are Analyze, Neuroimaging Informatics Technology Initiative (Nifti), Minc, and Digital Imaging and Communications in Medicine (DICOM) [350].

The amount of nibbles utilized to represent the data for every pixel is known as pixel depth. Each image is retained in a file and saved in a system memory as a collection of bytes. The minimal amount that can be saved in a system memory is a byte, which is a collection of 8 binary bits [351, 352].

A healthcare image is an organized collection of visual components called pixels or voxels that depicts the actual shape or activity of an anatomical area. It is a categorical formulation that links quantitative value to spatial positions as a consequence of a sampling/reconstruction operation. The amount of pixels employed to characterize a particular collecting modality’s field-of-view is an indication of the level of complexity that can be captured in the structure or operation [353, 354].

### Metadata

Metadata are details that the image uses to define itself. It may sound unusual, but in every storage format, the images has additional information beyond the image pixels. The file header containing the image dimensions with all coordinates, the depth of pixel, the spatial resolution and the photometric interpretation are contained in this data, which is known as metadata, which is normally put at the start of the document as a preamble [355, 356]. According to the characteristics of medical datasets, metadata play a larger significance in this situation. Images obtained from diagnostic modalities frequently contain details about the image’s creation. For instance, the pulse sequence used to create a magnetic resonance image will contain characteristics such as time accusation, flip point of view, amount of acquisitions, etc. The radio-pharmaceutical that was administered and the patient’s weight are both included in nuclear medicine images like PET images [357, 358].

### Model Building

**Fig. 25:**
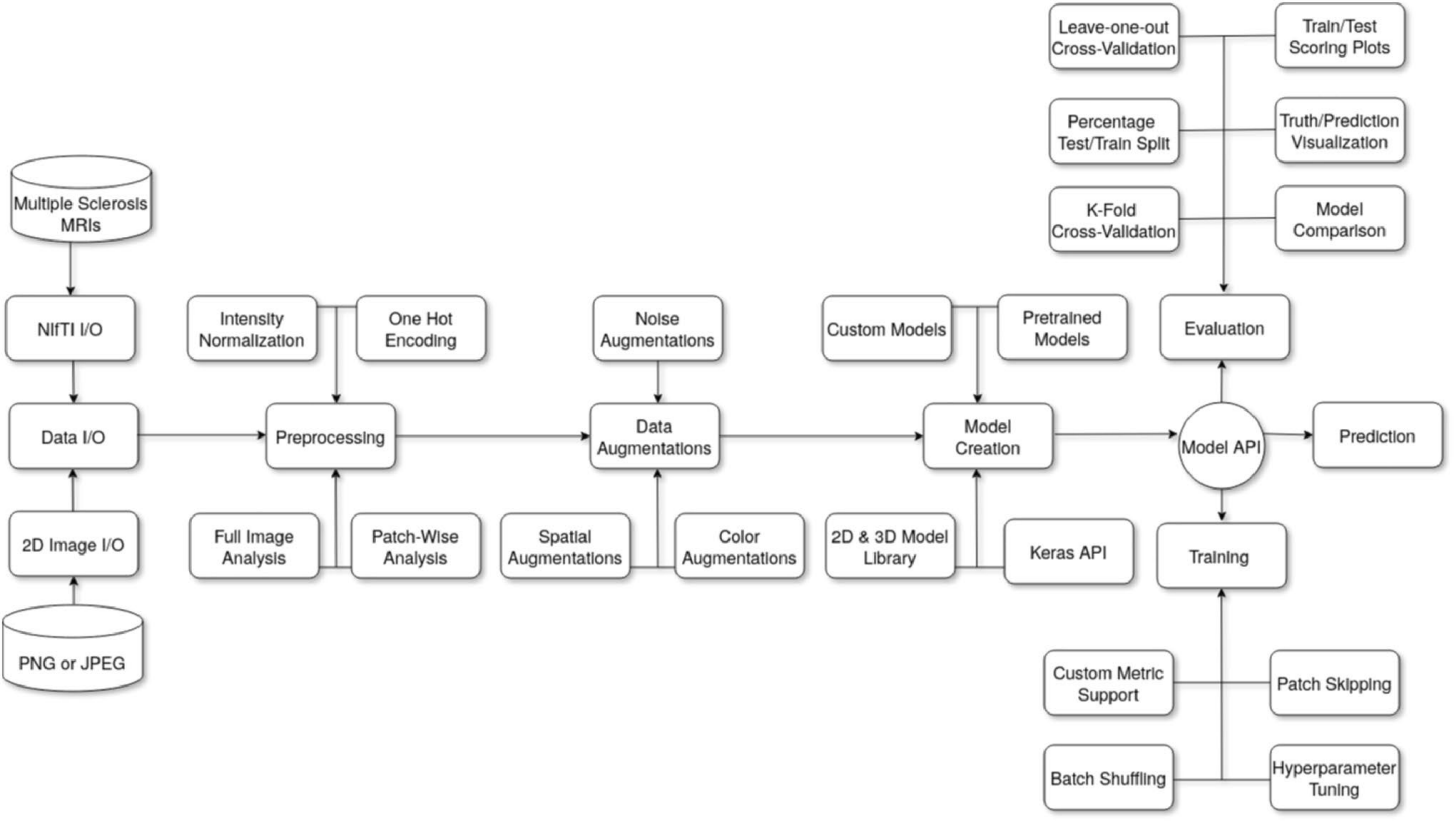
Flowchart showing the data inputs from different modalities at the beginning and a training pretrained or custom deep learning model at the conclusion

The construction of an acceptable deep learning based CNN model for a particular issue in medical image analysis is challenging and necessitates extensive analysis and testing. Before selecting a CNN model that best achieves their goals, professionals must become knowledgeable in a variety of models and evaluate their efficiency traits [31, 359]. Figure 25 depicts the building of a deep learning model comprises all necessary step that we follow.

For instance, there is currently no universal way to evaluate a predetermined the ideal configuration of neurons and layers for an CNN model provided a scenario statements. A popular strategy begins with an educated assumption based on past knowledge about networks used to solve associated issues. This assumption might be based on first-hand knowledge from a person or second or third-hand knowledge from a training deep learning program, weblog, or academic publication. While choosing a course of action, the analyst may then experiment with other options and thoroughly evaluate the classification results [360–362].Severa hyper-parameters communicate with the dimensions and height of deep learning neural networks, thus altering one hyper-parameter can have an impact on the others. A straightforward step-by-step strategy is to build a CNN network with multi-level hidden layers that are the same or variable size as the source:

- Try adjusting the CNN network’s height and breadth with multiple hyper-parameters [363].
- Try various methods (such as dropout, pooling, kernel/filter, regularization, momentum, decay, learning rate, activation functions, optimization algorithms, batch size and loss functions, etc.) as well as eliminating certain cells [364, 365].
- Choose a model that is more accurate altogether after making a few tweaks.

There will constantly be improved algorithms, thus users should really not waste their time fine-tuning deep learning models. Forming a realistic assumption of precision is aided by investigating the data. To set goals to beat, start with straightforward linear tactics. Various deep learning algorithms could be more useful, quick, and efficient than your initial choice [366–368].

### Training, Testing and Validation

For the purpose of developing reliable machine learning algorithms, the source data must be divided into training, testing, and validation sets. Beginning with a training dataset is necessary for algorithms for machine learning. In order to lessen this disparity, it measures the discrepancy between both the results that were anticipated and those that really occurred throughout each repetition. The generated technique is specially adapted to the training set of data. The finished method and its learned characteristics are evaluated on a different evaluation data set to see how generalisable they are. Training, validating, and evaluation samples are often required for deep learning based CNN models. In real-world situations, the entire population (dataset) is initially divided into a training and a testing datasets. A training and a validation datasets are created from the learning materials (training datasets). To adapt the model, learning samples are utilized. A authentic assessment of the algorithm during learning is provided via validation samples. Testing datasets allow for an immediate assessment of the finished product. The ideal distribution of the three different samples is not predetermined [369, 370] K-fold cross-validation is the process of repeating the division K time periods and averaging the correctness to make sure the model is impartial. The biggest problem that could occur is the system becoming over-fit to a particular training dataset. Whenever this happens, the system will be very efficient on the training data but extremely wrong on the testing set of data (low false negative rate among expected and real outcomes). Another distinct validation samples is applied to get around this.

### Sensitivity Analysis

[371] made the initial suggestion for sensitivity analysis in the context of misdiagnosis brought on by weight perturbations as a result of chaotic intake and system inaccuracy [371]. Since then, the phrase “sensitivity analysis” has become saturated with connotations that are tied to one another. The subject of neural network susceptibility to parameter error has indeed been extensively studied and documented [372]. Sensitivity analysis is defined here as the investigation of the impact of input modifications on system projections. The method that employs technique sensitivity analysis for cell segmentation is the one that is most similar to the one that is provided here [373]. The overall concept is the same in this study, but it varies in a number of ways, including the use of automated measurement discovery and the emphasis on computing efficiency [374]. Deep learning unpredictability is measured using a variety of techniques called Sensitivity assessment. It examines the significance of each source variable’s characteristic for every outcome. Comparing the system outcomes with all factors involved and the model with one element eliminated, or maintaining the quantities of all other factors and simply adjusting the strength of one input element, allows for the measurement of the effect of each input variable [375]. Sensitivity and Specificity analysis is essential for the actual uses of deep learning applications in the real life; it clearly and unambiguously demonstrates the extent to which CNN model output depends on each factor and gives medical practitioners and specialists more authority, particularly when the recent discoveries are catastrophic events that may represent additional anomalies that surpasses the predictive power of CNN models [376, 377]. Categorization method sensitivity assessment can be qualitative or quantitative. In the qualitative scenario, both the initial data and its altered (for example, flipped) variants are used in the categorization process. The researcher is shown the classification filters as overlays over the modified photos for review. A ground truth separation of the source image must be available for quantitative sensitivity evaluation. By computing a comparison measure between the predicted and reality, the categorization efficiency of the algorithm may then be assessed. According on the conversion (such as color and brightness modifications), the ground truth mask either stays unchanged or also has to be altered (e.g., flip, crop and zoom).

### Training Optimization

Optimization approaches, as a turbocharger in neural network techniques, perform a significant role in this subject. Due to their quick convergence, adaptive optimizers like ADAGrad [378] and ADAM [379] have revolutionized deep learning for the the past several years. The fundamental principle of these optimizer is to take gradient history into account while changing variables in the phase cycle.

The majority of optimization techniques have been developed, evaluated, and assessed in the research for classification techniques where the prediction range is generally constrained to a few dozen examples. Nevertheless, since each pixel is projected to have a tag in healthcare image classification issues, the forecast of state space is substantially greater; as a result, the usual optimizer of selection may not always be the best option [380].

For many newcomers, tweaking Artificial intelligence systems is a necessary but hard step. In order to reduce the objective functions, hyperparameters are adjusted. Optimization techniques are programs that modify characteristics like batch size, epcohs, weights and learning rates to reduce losses. Adaptive Moment Estimation, Nesterov’s Accelerated Gradient (NAG), Gradient Descent (GD), AdaDelta, and other frequently implemented optimizers are examples [381]. The majority of minimum locations discovered by gradient-based optimizers are local minima, which presents a frequent difficulty. Since the gradient gets less as training progresses and the learning rate is too high to approach the correct response, it is challenging to find the global minima. The genetic algorithm, which integrates the idea of development to ML, seems to be another method. Only the finest models emerge at the conclusion of the procedure, which is performed several occasions [382]. Every optimization technique has weaknesses. A one-size-fits-all approach cannot adjust to every dataset and accelerate learning to arrive to minima more quickly. The learning must swiftly converge to the goal with the least amount of loss in the fewest possible repetitions or epochs. It is important to properly solve the gradient disappearing concern (the gradient is too tiny to adjust the weight in the following loop) [383, 384].

#### Parallel Computing

Parallel computing or multitasking is a useful technique in medical image analysis since it increases the effectiveness of deep learning based medical image segmentation, classification, registration and other operations. The first concerns the continuously growing size of healthcare data especially images that is now available as a result of improvements in both research and development methods and mathematical simulation or visualization of healthcare data [380, 385].

The second argument is that AI algorithm intricacy is rising. The complexity of ML models, particularly DL models, is increasing as predictive performance is sought for. As an illustration, the Microsoft Turing natural language generation model comprises 17 billion parameters. Due to these two factors, training a sophisticated AI algorithm without concurrently computation may take many days or even months [386, 387].

Numerous initiatives have been made to investigate ways to enable concurrent ML from various angles [388, 389]. The following is a summary of three concurrent ML potential, the first of which is applicable to all ML jobs and the second and third of which are exclusive to medical image analysis using deep learning. The first potential is the need to create a unified system that combines simultaneous deep learning algorithm with parallel hyper-parameter adjustment [390, 391].

Nowadays, these two activities are frequently carried out using various technologies, such as Spark for concurrent hyper-parameter tweaking and Tensorflow for simultaneous deep learning. Still required is a framework or method that supports both effectively but is more integrated. The capability for concurrent training on top of datasets for the system based on medical image analysis using deep learning. Current collaborative initiatives on quickly acquiring and analyzing DICOM and Nifti datasets. However, it is currently unclear how to incorporate these methods with machine learning. The final chance is concurrent machine learning capability for Histopathological, MRI, CT and X-ray datasets containing images with clinical details, which are typical of the medical image analysis. When splitting clinical data and medical images, as opposed to conventional independent and identically distributed (IID) datasets, the clinical/medical dependency and correlation would be broken. Therefore, parallel deep learning using clinical data and medical images has to be given specific consideration [392–394].

The training issue, the throughput constraints, and the accessible budget all play a role in selecting the best infrastructure for DL. The growth of Graphics Processing Units (GPUs) and advances in parallel computing have considerably boosted the performance of Deep Neural Networks for clinical applications. Due to their efficient massively parallel processing architecture for both training and interpretation techniques, Graphic Processing Units are crucial in deep learning. An integrated GPU chip on the computer system is needed for the usual operation. There are hundreds of Arithmetic Logic Units (ALUs) for each GPU core. The very identical directions will be applied to a significant number of neurons in CNN through each layer [395]. It’s fairly usual to parallelize processing operations over numerous GPUs because a single GPU can’t handle large-scale deep learning delegates. By utilizing additional dispersed resources, distributed computing is an effective concurrent method to improve DL efficiency [396, 397].

#### Explainable AI

The term smart healthcare describes the application of techniques like cloud computing, the Internet of Things (IoT) and artificial intelligence AI to create a healthcare system that is effective, accessible, and individualized [1]. These platform enables real-time medical surveillance via medical apps on smartphones or wearables, enabling people to take charge of their own healthcare. In addition to being communicated with doctors for additional assessment, user-level health records may also be utilised in conjunction with AI for illness monitoring, early diagnosis of diseases, and specific medication assessment. There is a demand for Algorithms that can be described in the medical field due to the ethical dilemma of openness around AI and the absence of confidence in the black-box functioning of AI systems [398]. Explainable AI (XAI) approaches are the AI approaches that are used to illustrate AI models and their outcomes [399].

Complicated deep learning models (e.g., U-NET, DenseNet, ResNet, Inception and other GAN based models) cannot offer a self-explainable explanation for their outcomes in comparison to basic or tree-structured Machine learning (e.g., linear regression, DT, Bayesian, RF). Machine learning techniques should include description, according to several medical image analysis scientists, to make them easier to grasp and increase user confidence. The initial “black-box” model may be examined using explainable AI (XAI) tools, which offer “explanations” that give a subjective knowledge of the interactions among model elements and projections.

This procedure provides solutions to inquiries concerning the model,, including which aspects are most crucial and the reasons why some aspects influence outcomes more than others. It also offers information that enables the algorithms to be changed in a significant way. Molnar et al. [400] provide an assessment of frequent comprehensible procedures. The necessity for model-agnostic or model-specific procedures, the level of description necessary, and spatiotemporal or computational limits can all be key considerations when choosing XAI approaches.

The inability of existing XAI techniques to identify issues in the training sample as well as their emphasis on RGB images and user-friendliness for higher dimensional space visuals are among its drawbacks [401]. Given the issues, XAI offers possibilities for enhancing healthcare based CNN models. XAI might identify artifacts that lead to inaccuracies in numerical algorithms.

#### Generalization

The traditional objective of generalization is to improve the performance of learned AI models on testing samples. It gets challenging, though, because the training sample is a tiny fraction of the enormous medical images sample. Maintaining the ideal balance among over-fitting and under-fitting is no more sufficient for deep learning based medical image analysis since algorithms developed in one location at one time could not be applicable in another location at a later date. Conventional statistically learning algorithms, meanwhile, do not generalize effectively on fresh data with various distributions, which is a major contributor to typical AI errors. A significant barrier to using AI in medical image analysis research is identifying a sound generalization approach to make model performed outside of the training samples [402, 403].

Generalization philosophy, which is still in its infancy, could be able to offer solutions to these issues. The generalization of AI has been extensively researched. The Ockham’s Razor principle described by Ariew [404], it demonstrates that a positive estimated results is more likely to be due to factors other than the idiosyncrasies of the selected examples the simpler the theory is. The boundaries between inadequate and excessive training sample learning are hazy. The division of data into a training sub-samples and an assessment subset is one of the traditional techniques for identifying under-fitting or over-fitting [405]. The software will execute the classification classifier on the testing subset at each iteration process in order to determine the accuracy rate on data that are not in the initial training pool. The algorithm is overfitting if the precision of testing samples begins to steadily decline. On the other hand, it indicates that the system is still under-fitting if the evaluate the efficiency hasn’t achieved its optimum [210].

Finding a balance among bias (under-fitting) and variance (over-fitting) requires a generalize and proper technique. Cross-validation is a popular method for ensuring that there is no accidental learning bias. Another method used to improve how well a generalize learning method is regularization. It concentrates on minimizing the effects of noise data, which are arbitrary mistakes and coincidences rather than the true properties of the collection [406, 407].

In order to lower the likelihood of inaccurate generalization, it avoids training increasingly complex models. A recently suggested technique for neural networks called “dropout” involves arbitrarily removing units to make the following levels rely entirely on the connections to the different layers above them. However, no technique can escape continual, extensive modification to improve the CNN model’s generalization [408, 409].

An appealing aspect of artificial intelligence is that as a machine is supplied with greater samples, productivity will increase. It will ultimately, nonetheless, come up against some restrictions imposed by the training ability of the system. Many DL algorithms have excessive parameters and are susceptible to bias as they learn additional noise data. In a long-term operational run, solving the generalization issue will enable AI systems of the medical image analysis’s system far more robust and noise-proof. An automated method that self-adjusts in absorbing data by evaluating their reliability would be a strategy and deliver. Data that might potentially cause the network to become unstable should immediately be assigned less weight in the propagation, and their effects on subsequent updates must be minimized [410, 411].

#### Uncertainties in CNN Based Medical Models

Primarily, deep learning frameworks are procedures made up of a collection of instructions that use optimization and random number synthesis to establish parameters of the model. As a result, deep learning models created using the same information are practically never identical. Data and knowledge-based ambiguities combine to form the uncertainty of machine learning applications. Aleatory uncertainty, which is not created by the framework but is irreducible, is the term for the uncertainty connected to the intrinsic disturbance of the actual figures. Epistemic uncertainty, which is frequently the outcome of the discrepancy among the facts in system training and forecasting, is the ambiguity brought on by insufficient information and data [412].

We must evaluate the level of uncertainty in each data source used as input to ML models and comprehend how uncertainty spreads inside the model in order to be able to measure aleatory uncertainty. The standardized production intricacy can make this difficult for deep learning models. A deep learning model’s ultimate outcomes can be significantly altered by a tiny variation in the raw data. The problem of generalization is connected to the epistemic uncertainty. Since the majority of machine learning models are built using a particular set of facts, it may be difficult to generalize the algorithm to situations that are not included in the entire sample.

It can be exceedingly difficult to precisely measure the uncertainty associated with generalization due to the lack of information in the initial data collection. For deep learning models to be more user-friendly and to build trustworthiness, precise uncertainty assessment is crucial. There are several mathematical and statistical approaches that have been suggested to deal with uncertainty quantification.

The best often used techniques may be divided into two categories: ensemble uncertainty quantification and Bayesian uncertainty quantification. In Bayesian uncertainty quantification strategies, the learning sample is used to approximate the posterior probability distribution [412]. Ensemble uncertainty quantification entails training many models, computing the synthesized estimate (for example, mean), and computing the variance to represent uncertainty. Subsequently, many Monte Carlo model [413] modifications for uncertainty quantification, as Monte Carlo Dropout [414] have been developed to more effectively quantify forecasting uncertainty.

### Model Development

Massive hyper-parameter ranges and lengthy training duration are characteristics of contemporary deep learning. These characteristics drive the requirement for the development of sophisticated hyperparameter optimization capability in computing environments environments, along with the growth of distributed programming and the increasing desire to productionize deep learning applications [415, 416]. The procedure of selecting an appropriate framework or tailoring a linked framework for one or more training samples is known as model development. Candidates for off-the-shelf models comprise ensemble models like Random Forest, XGBoost, Support Vector Machine, Decision Tree and the majority of Deep learning as well as individual models like AlexNet, Inception with different flavors, DenseNet, U-Net, V-Net, U-$\hbox {Net}^{++}$ and SegNet [417].

There is a high need for Auto Machine Learning (AutoML) that does not involve specialized expertise or human tweaking since discovering ideal models or connecting new models takes time and may never be sufficient. For instance, considering that the efficiency of an deep learning and machine learning methods are data-dependent such as Optuna, OptiML, AutoScikit-learn, and AutoWeka employ Bayesian optimization techniques (Exploration and Exploitation), Evolutionary method (NSGAIISampler (Non-dominated sorting genetic algorithm [418])) to forecast the model ’s effectiveness on a particular dataset [415, 419].

For example, to minimize resources, OptiML can develop a regression model to forecast the efficiency of more models that haven’t yet been evaluated. Bayesian optimization, meta-learning, and ensemble building are also utilized in Auto-Sklearn’s hyperparameter tweaking. Significant issues, however, stay unanswered. Initially, distinct models should be chosen based on distinct use cases using distinct optimal measurement parameters. Furthermore, huge data training does not benefit from the cross-validation approach. Third, consistency, dependability, computational cost, and generalizability are all highly essential and sometimes ignored when looking for remedies. Achievement on precision shouldn’t be the sole consideration [420].

In addition to predictive efficiency, a strong AutoML system should autonomously generate a model that addresses all issues with huge data, scenario adaptation, and exhaustive measurements. Although there is a recognized need for more deep learning specialists in business and academia, these professionals are challenging to by and difficult to teach. AutoML may fill that vacuum and lead to several new career possibilities in the deep learning field, including those in medical image analysis. Model selection would be rapid and simple with AutoML and the difficulty of comparing Ml algorithms would be significantly lower. Deep learning-powered value-added models and applications wouldn’t just be available to the biggest technology and software companies [421, 422].

Small clusters will also be capable of rapidly construct reliable programs to imitate the actual world, collect useful information,, and direct the development of climate change and environmental policies. For the upcoming version of deep learning based medical analysis applications, new avenues will be made available [423, 424].

### Provenance, Reproducibility, Replicability, & Reusability

The foundation of the scientific methodology is reproducibility. Its broadest and most typical interpretation relates to the capacity to replicate the results of a certain exploratory studies. This is a prerequisite (but not the only one) for a research claim to be acknowledged as incoming knowledge [425, 426]. The phrase “producing and creating and then executing novel software based on the description of a computational model or procedure supplied in the original release, and producing outcomes that are close enough” is used to complete the definition of “replicating a documented finding” [427, 428].

For deep learning based medical image analysis research, there are four major issues that are connected:

- **Provenance:** What changes had the dataset go through before the results were published? Where did the training set, AI algorithm, software, and hardware come from?
- **Reproducibility:** Utilizing the identical facts and methodologies, can a third party independently verify the accurate AI procedure and outcomes identified?
- **Replicability:** How could an alternative party achieve the equivalent findings using equivalent (but not exact) machine learning (ML) analysis on similar (but not always the very similar) statistics?
- **Re-usability:** refers to how readily previously trained AI models may be deployed to fresh data or to different circumstances.

### Workflow Automation of Healthcare Application

Deep Learning designing is a broad field that encompasses numerous technologies, methodologies, algorithms, techniques and libraries. Its product procedures are made up of a number of connections between hardware and software such as a raw data provider (source) and the sharing of useful information, online services (Web services), APIs and other utility software. It would be impractical to manage every piece manually [429]. Making deep learning based healthcare application usable in real-world circumstances requires optimizing every procedure. Workflow automation for deep learning based healthcare application is still being developed. The Computer Aided Diagnosis society requires a more efficient method for managing the lifespan of established deep learning models in order to sustain artificial intelligence acceptance and scalability [430, 431]. The technique of integrating an investigational machine learning model into a live online system is known as MLOps (MLDevOps). Production-level ML models are deployed, monitored, managed, and governed by it. There are numerous chances for open-source software programmers to engage on this project in the future [432].

Implementing effective and profitable deep learning based healthcare models needs the cooperation of many different organizations and assets, as well as a variety of different technologies, algorithms, scripts, libraries, and tools for automating data preprocessing, digestion, training, validation, and production. To finish a challenging task, a workflow, or logically connected stream of several procedures, is needed [433–435].

Process coordination can be done in a variety of methods, such as by scripting a Shell script, a Python notebook, or by utilizing workflow management software like Cylc. Processes involves fundamental elements are identical. Atomic operations and linkages exist in every workflow. Process automation is the automated execution of all atomic operations without user intervention after a process has been initiated [436]. Workflow management software (WfMS) such as Pegasus-WMS, Apache Airflow, Galaxy, Cylc and Geoweaver [437] and others are being developed to enable automation. These Workflow management software can increase the replicability and reproducibility of CAD discoveries by not only automating the procedure but also documenting the provenance [438].

### AI Ethics

With an unforeseen tremendous potential of predicting the healthcare future and handling biological risks and materials in preparation to protect lives and safeguard the humanity, healthcare AI is built to defend humankind. The ability has a limitation, though, and it can’t save everybody in an incident like a COVID-19 or catastrophic one. What about if healthcare AI makes a mistake, overlooks a region or population, underestimates the impact, and causes more deaths or even more widespread injury? Healthcare AI is a sophisticated but still non-existent system that lacks legal status. However, it acts with a certain amount of self-will and its choices have an influence on society [439, 440].

The ethical issues raised by artificial intelligence as it is deployed are the subject of a multitude of study [441]. Researchers have looked at the connection between the oppression of racial minorities by computer aided diagnosis systems and how it reinforces preexisting prejudice, as well as the impact that cultural attitude performs in computational unfairness. Ultimately, there will likely be several rules and laws governing artificial intelligence morality on healthcare systems [442, 443].

In this article, we briefly discuss a few of the various routes approaching more moral AI in the computer aided diagnosis and healthcare sciences, including more accessible datasets and impartial algorithms. By collaborating with social scientists, medical practitioners and academics who are currently researching the societal consequences of deep learning in the fields of enforcement, legislation, economics and technologists should build computer aided diagnosis ethics-related logic. This involves creating guidelines for deep learning researchers to interact with ethics as both a theoretical and practical endeavor, as the gathering of data and the selection of one theory over another has an immediate influence on healthcare system and people [444–446].

Especially over the past but not least, we think that for there to be an ethical and equitable progression in artificial intelligence in healthcare, one must communicate their implementation of any machine learning or deep learning to the larger society it influences (for instance, if an automated method for developing COVID-19 detection will immediately affect on representations of healthcare system) [447, 448].

## Tooling and Services

Strong computing is required due to the massive data structure of medical image analysis using deep learning and the great sensitivity of artificial intelligence algorithms. The common hardware and software for computer aided diagnosis are described in this section.

### Computing Device

Graphics Processing Units (GPU), Central Pciterocessing Units (CPU), Field-Programmable Gate Arrays (FPGA) and specialized processors (such as TPU - Tensor Processing Unit) are often utilized deep learning devices. Due to their ability to accelerate matrix operations and convolution calculations, GPUs are dominating. In deep learning, the weights are changed after each cycle and kept in memory or a temporary cache so they may be used in subsequent iterations. Compared to Central Processing Units, Graphics Processing Units offer larger memory bandwidths and are better suited for demanding tasks and streaming memory models.

Additionally, researchers are currently looking at the subsequent AI intelligence revolution. Quantum computing, which was first hypothesized by [449], is thought to be the forthcoming major advancement because it can generate statistical formations that are computationally challenging for conventional computers to build [450–452]. Using the Internet of Things (such as edge devices, access points, wearable technology, mobile phones, sensors, etc.) with implanted artificial intelligence methodologies to analyze information locally without transferring much records is yet another option known as edge computing. This can decrease dependency on network systems and boost the artificial intelligence’s adaptability and usefulness [453, 454]

### Software

Operating systems developed from Linux that have ongoing, ongoing engineering assistance are advised. The artificial intelligence bellwether at the moment is Ubuntu, which has a lot of built-in components. Users may download and run GPU drivers like CUDA (a program that enables programming for NVIDIA GPUs) with ease, and Python package managers like Conda and Pip can make it easier to download and run packages. Jupyter server [455] (either of notebook, Lab, or Hub) is strongly advised for virtual machine interaction. It enables deep learning based healthcare scientists to perform their tasks, improve efficiency and facilitate cooperation by allowing them to generate and exchange their experimental studies scripts to complete result findings single document.

Python’s vibrant, freely available, and supportive library ecosystem is primarily responsible for the language’s prominence in the field of artificial intelligence. Several frequently used open-source libraries are Tensorflow, Pytorch, Keras, Theano, Channer, Caffe and Mxnet, OpenCV, AutoML, Scikit-Learn, Pandas, Matplotlib, Seaborn, Plotly, Numpy and etc.

## Summary

This article provides a summary of the state-of-the-art technology and the advancement of deep learning research with a concentrate on application areas of healthcare analysis especially medical image analysis. Innovations in computer aided diagnosis frameworks and theory will advance healthcare into its next stage. The healthcare community needs to keep up with the explosion in empirical datasets and construct usable deep learning models fast, accurately and affordably. Healthcare AI study and development remain in their adolescence and all the major challenges—from data to models to operations—can lead to a wide range of opportunities across all fields, including industry, academia, Research & Development organizations and government.
